# Widespread Fosfomycin Resistance in Gram-Negative Bacteria Attributable to the Chromosomal *fosA* Gene

**DOI:** 10.1128/mBio.00749-17

**Published:** 2017-08-29

**Authors:** Ryota Ito, Mustapha M. Mustapha, Adam D. Tomich, Jake D. Callaghan, Christi L. McElheny, Roberta T. Mettus, Robert M. Q. Shanks, Nicolas Sluis-Cremer, Yohei Doi

**Affiliations:** a Division of Infectious Diseases, University of Pittsburgh School of Medicine, Pittsburgh, Pennsylvania, USA; b Center for Innovative Antimicrobial Therapy, University of Pittsburgh School of Medicine, Pittsburgh, Pennsylvania, USA; c Department of Ophthalmology, University of Pittsburgh School of Medicine, Pittsburgh, Pennsylvania, USA; d Department of Microbiology, Fujita Health University, Aichi, Japan; Indiana University Bloomington

**Keywords:** Gram negative, fosfomycin resistance, genomics, glutathione *S*-transferase, phylogenetics

## Abstract

Fosfomycin is a decades-old antibiotic which is being revisited because of its perceived activity against many extensively drug-resistant Gram-negative pathogens. FosA proteins are Mn^2+^ and K^+^-dependent glutathione *S*-transferases which confer fosfomycin resistance in Gram-negative bacteria by conjugation of glutathione to the antibiotic. Plasmid-borne *fosA* variants have been reported in fosfomycin-resistant *Escherichia coli* strains. However, the prevalence and distribution of *fosA* in other Gram-negative bacteria are not known. We systematically surveyed the presence of *fosA* in Gram-negative bacteria in over 18,000 published genomes from 18 Gram-negative species and investigated their contribution to fosfomycin resistance. We show that FosA homologues are present in the majority of genomes in some species (e.g., *Klebsiella* spp., *Enterobacter* spp., *Serratia marcescens*, and *Pseudomonas aeruginosa*), whereas they are largely absent in others (e.g., *E. coli*, *Acinetobacter baumannii*, and *Burkholderia cepacia*). FosA proteins in different bacterial pathogens are highly divergent, but key amino acid residues in the active site are conserved. Chromosomal *fosA* genes conferred high-level fosfomycin resistance when expressed in *E. coli*, and deletion of chromosomal *fosA* in *S. marcescens* eliminated fosfomycin resistance. Our results indicate that FosA is encoded by clinically relevant Gram-negative species and contributes to intrinsic fosfomycin resistance.

## IMPORTANCE

There is a critical need to identify alternate approaches to treat infections caused by extensively drug-resistant (XDR) Gram-negative bacteria. Fosfomycin is an old antibiotic which is routinely used for the treatment of urinary tract infections, although there is substantial interest in expanding its use to systemic infections caused by XDR Gram-negative bacteria. In this study, we show that *fosA* genes, which encode dimeric Mn^2+^- and K^+^-dependent glutathione *S*-transferase, are widely distributed in the genomes of Gram-negative bacteria—particularly those belonging to the family *Enterobacteriaceae*—and confer fosfomycin resistance. This finding suggests that chromosomally located *fosA* genes represent a vast reservoir of fosfomycin resistance determinants that may be transferred to *E. coli*. Furthermore, they suggest that inhibition of FosA activity may provide a viable strategy to potentiate the activity of fosfomycin against XDR Gram-negative bacteria.

## INTRODUCTION

Fosfomycin is a broad-spectrum cell wall synthesis inhibitor produced by some strains of *Streptomyces* spp. and *Pseudomonas syringae* (1). It exerts antibacterial activity by inactivating the cytosolic *N*-acetylglucosamine enolpyruvyl transferase (MurA), which prevents the formation of *N*-acetylmuramic acid, an essential component of peptidoglycan (2). It maintains excellent activity against the majority of *Escherichia coli* clinical isolates and is now one of the first-line agents endorsed for the empirical treatment of uncomplicated urinary tract infection (3). However, other Gram-negative species exhibit lower susceptibility to fosfomycin (4). For example, the MIC_50_ values for *Klebsiella pneumoniae* range between 16 and 32 μg/ml, compared to 0.5 to 1 μg/ml for *E. coli* (5, 6). In Gram-negative bacteria, fosfomycin resistance can be conferred by (i) defects in the transporters across the cytoplasmic membrane, (ii) amino acid substitution in the MurA active site which decreases fosfomycin binding affinity, and (iii) production of the fosfomycin-inactivating enzyme FosA (7). FosA is an Mn^2+^- and K^+^-dependent dimeric glutathione *S*-transferase that catalyzes the nucleophilic addition of glutathione to the epoxide ring of fosfomycin (8). FosA can be encoded on a bacterial chromosome or a plasmid. For instance, the first *fosA* gene, described in *Serratia marcescens*, is carried on transposon Tn*2921* located on a conjugative plasmid (9, 10), but it has high identity with chromosomal *fosA* of *Enterobacter cloacae*, where it likely originated. Similarly, *fosA5* and *fosA6* located on *E. coli* plasmids likely originated on the chromosome of *K. pneumoniae* (11, 12). The most commonly reported plasmid-mediated *fosA* gene is *fosA3*, which is widely distributed in *E. coli* and other *Enterobacteriaceae* species in East Asia but whose chromosomal progenitor is unknown (13–15). The goals of this study were to systematically survey for the presence and distribution of the *fosA* genes in Gram-negative bacteria in published genome sequences, to confirm their contribution to fosfomycin resistance, and to catalog their genetic diversity. Insights into FosA-mediated intrinsic fosfomycin resistance in Gram-negative bacteria would inform approaches to potentiate the activity of fosfomycin against extensively drug-resistant (XDR) Gram-negative bacteria.

## RESULTS

### FosA is widely distributed among Gram-negative pathogenic species.

A total of 18,130 published genomes from 18 clinically relevant Gram-negative species were downloaded. They were queried for FosA-like sequences at a cutoff of 40% similarity to a collection of diverse FosA sequences by BLAST. FosA was frequently identified in the genomes of *Providencia stuartii* (100%), *K. pneumoniae* (99.7%), *S. marcescens* (99.7%), *Pseudomonas aeruginosa* (98.8%), *Enterobacter aerogenes* (98.4%), *Klebsiella oxytoca* (96.6%), *Morganella morganii* (90.5%), *Providencia rettgeri* (85.7%), and *Enterobacter cloacae* (82.4%), which were likely to be on the chromosomes based on the high prevalence (Table 1). FosA was also intermittently found in *Proteus mirabilis* (16.7%), *Salmonella enterica* (9.8%), and *Acinetobacter pittii* (7.8%). In contrast, it was rarely identified in *E. coli* (4.6%), *Citrobacter freundii* (3.8%), *Acinetobacter baumannii* (2.0%), *Achromobacter xylosoxidans* (0%), *Burkholderia cepacia* (0%), and *Stenotrophomonas maltophilia* (0%). Table S1 in the supplemental material shows the numbers of genomes evaluated and the rates of FosA homologues by time periods.

10.1128/mBio.00749-17.2 TABLE S1  Genome data submitted by year groups. Download TABLE S1, PDF file, 0.4 MB. Copyright © 2017 Ito et al. 2017 Ito et al. This content is distributed under the terms of the Creative Commons Attribution 4.0 International license.

**TABLE 1:** Distribution of FosA in 18 Gram-negative species

Species	Total no. of genomes	No. of genomes containing *fosA* homologue (%)	No. of FosA alleles^a^
*Providencia stuartii*	10	10 (100)	5
*Klebsiella pneumoniae*	1,631	1,626 (99.7)	104
*Serratia marcescens*	311	310 (99.7)	37
*Pseudomonas aeruginosa*	2,257	2,231 (98.8)	68
*Enterobacter aerogenes*	122	120 (98.4)	30
*Klebsiella oxytoca*	89	86 (96.6)	30
*Morganella morganii*	21	19 (90.5)	8
*Providencia rettgeri*	7	6 (85.7)	5
*Enterobacter cloacae*	489	403 (82.4)	144
*Proteus mirabilis*	60	10 (16.7)	3
*Salmonella enterica*	5,416	533 (9.8)	17
*Acinetobacter pittii*	102	8 (7.8)	6
*Escherichia coli*	5,363	246 (4.6)	22
*Citrobacter freundii*	78	3 (3.8)	3
*Acinetobacter baumannii*	1,915	39 (2.0)	9
*Achromobacter xylosoxidans*	35	0	
*Burkholderia cepacia*	94	0	
*Stenotrophomonas maltophilia*	130	0	

a Several of the FosA alleles were found in more than one species.

### Phylogenetic analysis reveals significant diversity and interspecies acquisition of *fosA*.

**FIG 1:**
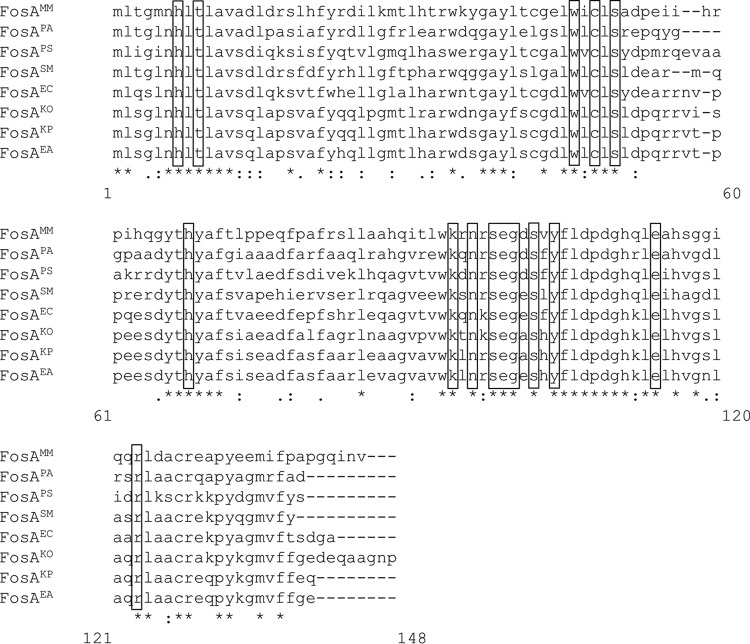
Amino acid alignment of representative chromosomal FosA. Amino acids in boxes represent active site residues. MM, *M. morganii*; PA, *P. aeruginosa*; PS, *P. stuartii*; SM, *S. marcescens*; EC, *E. cloacae*; KO, *K. oxytoca*; KP, *K. pneumoniae*; EA, *E. aerogenes*.

The amino acid sequences of FosA in *K. pneumoniae*, *K. oxytoca*, *E. cloacae*, *E. aerogenes*, *S. marcescens*, *P. aeruginosa*, *M. morganii*, and *P. stuartii* shared 80%, 76%, 71%, 80%, 68%, 60%, 55% and 62% identity, respectively, to that of FosA3, the most common plasmid-mediated FosA found in *E. coli*. The identity between FosA in *K. pneumoniae* and *E. aerogenes* was notably high (97%), which likely reflects the close genomic relationship of the two species (Fig. 1) (16).

**FIG 2:**
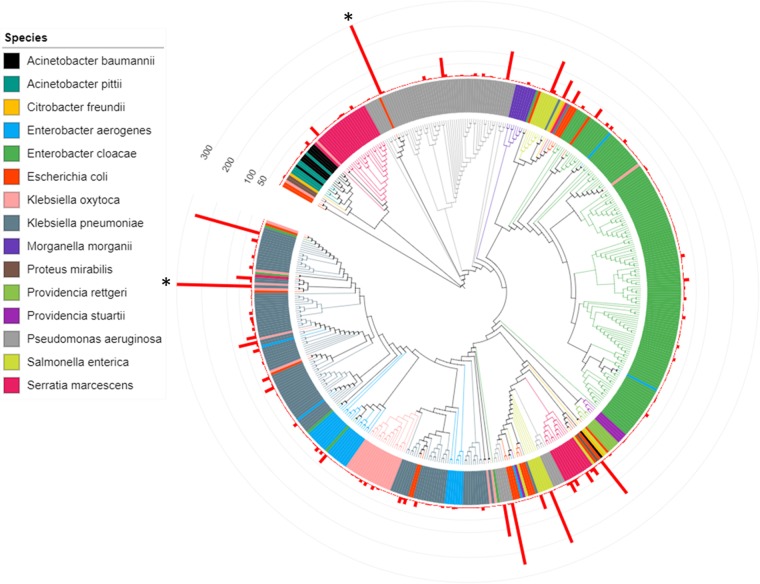
Phylogeny of 473 FosA amino acid sequences identified across 15 Gram-negative species. The red bars represent number of genomes with a given FosA sequence in a given species. Bars with frequencies of >300 (*) are truncated for clarity.

A total of 473 distinct FosA protein sequences were identified across the species investigated in this work (Fig. 2 and Data Set S1). Phylogenetic analyses revealed extensive FosA sequence diversity both within and between species (Fig. 2). The crystal structure of FosA^PA^ in complex with fosfomycin previously revealed key residues in the enzyme’s active site responsible for Mn^2+^, K^+^, and fosfomycin binding (Fig. S1) (17). Of note, all of these residues (H7, T9, W46, C48, S50, H64, K90, N92, S94, E95, G96, S98, Y100, E110, and R119) are highly conserved across the different FosA proteins. These residues are equivalent to H7, T9, W46, C48, S50, H68, K94, N96, S98, E99, G100, S102, Y104, E114, and R123 in the present study (Fig. 1). Shared FosA sequences between species and high diversity of FosA sequences within several species suggest the likely occurrence of lateral acquisition of *fosA*, presumably through acquisition of plasmids.

10.1128/mBio.00749-17.1 FIG S1  Structural representation of the FosA^PA^ active site illustrating key residues involved in K^+^, Mn^2+^, and fosfomycin binding. The figure was generated using PDB 1LQP (C. L. Rife, R. E. Pharris, M. E. Newcomer, and R. N. Armstrong, J Am Chem Soc 124:11001–11003, 2002). Download FIG S1, TIF file, 1.9 MB. Copyright © 2017 Ito et al. 2017 Ito et al. This content is distributed under the terms of the Creative Commons Attribution 4.0 International license.

10.1128/mBio.00749-17.3 DATA SET S1  Distinct FosA protein sequences. Download DATA SET S1, PDF file, 0.1 MB. Copyright © 2017 Ito et al. 2017 Ito et al. This content is distributed under the terms of the Creative Commons Attribution 4.0 International license.

### Chromosomal *fosA* genes confer high-level fosfomycin resistance.

*E. coli* TOP10 was transformed with recombinant plasmids pFosA^KP^, pFosA^KO^, pFosA^EC^, pFosA^EA^, pFosA^SM^, pFosA^PA^, pFosA^MM^, and pFosA^PS^, carrying *fosA* from *K. pneumoniae*, *K. oxytoca*, *E. cloacae*, *E. aerogenes*, *S. marcescens*, *P. aeruginosa*, *M. morganii*, and *P. stuartii*, respectively. Most of these transformants of *fosA* were highly resistant to fosfomycin with a MIC of 1,024 μg/ml (Table 2). pUC57 is a high-copy-number plasmid, which may have caused some of the MICs to be very high. The MIC of fosfomycin in the transformant of *fosA*^PA^ was 16 μg/ml, which is considered susceptible though still representing a 16-fold increase from the baseline MIC of 1 μg/ml (18). The FosA activity of the transformants was inhibited by sodium phosphonoformate, as suggested by significant enlargements of the zones of inhibition upon its addition to the fosfomycin disk in most of species (Table 2). One exception was FosA of *P. aeruginosa*, for which the inhibition zone enlarged only by 6 mm, likely due to the modest baseline activity of FosA^PA^.

**TABLE 2:** Susceptibility of *E. coli* TOP10 transformants harboring chromosomal *fosA* from various species

Transformant	MIC (μg/ml)^a^	Zone diam (mm)	Zone diam with PPF (mm)	Original species	Accession number
*E. coli* TOP10 carrying plasmid					
pFosA^KP^	>1,024	6	18	*K. pneumoniae*	YP_005224903.1
pFosA^EC^	>1,024	6	20	*E. cloacae*	AIX57742.1
pFosA^EA^	>1,024	6	16	*E. aerogenes*	YP_004592226.1
pFosA^SM^	1,024	6	14	*S. marcescens*	WP_025303168.1
pFosA^PA^	16	22	28	*P. aeruginosa*	NP_249820.1
pFosA^MM^	1,024	6	16	*M. morganii*	WP_004238530.1
pFosA^KO^	1,024	6	20	*K. oxytoca*	WP_047724618.1
pFosA^PS^	>1,024	6	18	*P. stuartii*	WP_014658192.1
*E. coli* TOP10 alone	1	38	38		

a MICs were determined by the agar dilution method supplemented with 25 μg/ml glucose-6-phosphate. For disk testing, 1 mg of sodium phosphonoformate (PPF) was added to fosfomycin disks. The *fosA* genes were cloned and constitutively expressed on vector pUC57.

Fosfomycin MIC of *S. marcescens* K904 was reduced from 16 μg/ml to 0.5 μg/ml (32-fold decrease) when *fosA* was deleted in frame, confirming the role played by chromosomally encoded FosA in the reduced susceptibility of fosfomycin.

## DISCUSSION

In the present study, we revealed the distribution of FosA homologues in Gram-negative bacteria and their genetic diversity. Homologues of FosA were identified in most genomes of *K. pneumoniae*, *K. oxytoca*, *E. cloacae*, *E. aerogenes*, *S. marcescens*, *M. morganii*, *P. stuartii*, and *P. aeruginosa*, which represent species with intrinsic resistance or reduced susceptibility to fosfomycin (4, 6).

While resistance to fosfomycin can be caused by multiple mechanisms, including transporter defect, target modification, and FosA-mediated inactivation (2, 19, 20), it is notable that many Gram-negative species carry the *fosA* gene on the chromosome, whereas it is nearly absent on the *E. coli* chromosome. FosA was first reported on transposon Tn*2921* of a clinical strain of *S. marcescens* (10). However, this original FosA^Tn*2921*^ is distinct from FosA^SM^ encoded on the chromosome of *S. marcescens*. Instead, FosA^Tn*2921*^ is closely related to the chromosomal FosA of *E. cloacae*, suggesting that it was mobilized from the latter species by transposition. We recently reported that *fosA6* identified in a fosfomycin-resistant *E. coli* strain was likewise mobilized from the chromosome of *K. pneumoniae* based on its high-level similarity with *fosA*^KP^ and its location on a transposon (11). Our findings that *fosA* is widely distributed in Gram-negative bacterial species and confers resistance or reduced susceptibility to fosfomycin suggest that chromosomal *fosA* genes in Gram-negative bacteria may serve as a reservoir of fosfomycin resistance in species that lack *fosA*, such as *E. coli* (21). This is supported by phylogenetic evidence of frequent lateral exchange of *fosA* alleles among a majority of Gram-negative species in our study. Despite their genetic diversity, *fosA* genes from multiple species retain the capacity to confer fosfomycin resistance.

Despite the worldwide spread of extended-spectrum β-lactamases (ESBLs) in *E. coli*, fosfomycin remains active with a MIC_50/90_ of 0.5/2 μg/ml, respectively (19, 22, 23). However, other Gram-negative species are generally not as susceptible to fosfomycin as *E. coli*. For example, *K. pneumoniae* clinical strains producing KPC-type carbapenemase have a MIC_50/90_ of 16/64 μg/ml, respectively (5). The majority of *K. pneumoniae* strains are considered susceptible given the current susceptibility breakpoint of 64 μg/ml, which is applicable only to urinary tract infection, but a significant portion will be considered nonsusceptible by the lower susceptibility breakpoint (32 μg/ml) of the European Committee on Antimicrobial Susceptibility Testing (EUCAST). Therefore, clinical use of fosfomycin might be limited against these species. There is emergent interest in reevaluating fosfomycin for use in the treatment of infections caused by multidrug-resistant Gram-negative bacteria, orally for urinary tract infections and intravenously for systemic infections (24). Intravenous fosfomycin has been used in many countries outside the United States for years, and a phase 3 clinical trial of intravenous fosfomycin for the treatment of complicated urinary tract infection and acute pyelonephritis was recently completed (ClinicalTrials registration no. NCT02753946). However, the peak plasma concentration of fosfomycin after an oral dose is below the current breakpoint (20). While the plasma concentrations are much higher for intravenous fosfomycin, a wide variation between individuals has been noted (25). Therefore, whether fosfomycin can be utilized in the treatment of systemic infections caused by Gram-negative pathogens with reduced susceptibility to this agent due to intrinsic production of FosA remains uncertain.

Nomenclature of FosA has lacked consistency, likely due to the diversity of this family of enzymes, and also in part because of the ambiguity in regard to the origin and location of *fosA*, i.e., intrinsic versus acquired and chromosomal versus plasmid-mediated. From both clinical and One Health perspectives, FosA enzymes of concern are those which are acquired by species lacking intrinsic *fosA*. We therefore propose that chromosomal, or intrinsic, *fosA*/FosA be distinguished by adding the initials of the species, as we have done throughout this paper. For example, the *fosA* gene located on the chromosome of *S. marcescens* would be called *fosA*^SM^ (and its product would be called FosA^SM^). We acknowledge that there will be multiple intrinsic FosA sequences in many species, but they are likely to be functionally comparable, and the need to distinguish them would be minimal. In the case that a functionally significant allele is identified, it can be distinguished by adding the amino acid change of interest after the initials of the species.

There are currently 5 acquired FosA proteins described in the literature (Table 3). FosA^Tn*2921*^, as has been discussed, was the first such protein to be described as a component of Tn*2921* on plasmid pSU912 in *S. marcescens* (26). FosA^Tn*2921*^ is up to 100% identical to FosA^EC^. As FosA^Tn*2921*^ is the first plasmid-mediated FosA that was identified, and also in order to avoid its confusion with the chromosomal FosA inherent in this species (FosA^SM^), we propose designating FosA^Tn*2921*^ FosA1 (Table 3). FosA2 was reported as chromosomal FosA of *E. cloacae* (14) and therefore would be considered FosA^EC^ in our proposed nomenclature. FosA3 is the most commonly reported plasmid-mediated FosA whose origin remains unknown. FosA4 shares 93% amino acid identity with FosA3 and therefore likely has the same origin as the latter. FosA5 and FosA6 are 100% and 99% identical to FosA^KP^, respectively, and thus most likely originated in *K. pneumoniae*. FosA7 was recently reported as the chromosomal FosA of *Salmonella enterica* serovar Heidelberg of animal origin (27).

**TABLE 3:** List of plasmid-mediated FosA alleles

FosA allele^a^	Harboring species	Likely species of origin	Accession no.	Reference(s)
FosA1 (FosA^Tn*2921*^)	*S. marcescens*	*E. cloacae*	ACO52881.1	37
FosA2 (FosA^EC^)	*E. cloacae*	*E. cloacae*	ACC85616.1	21
FosA3	*E. coli*, *K. pneumoniae*, *E. cloacae*, *E. aerogenes*, *S. enterica*, *P. mirabilis*	Unknown	BAJ10054.1	13
FosA4	*E. coli*, *S. enterica*	Unknown	BAP18892.1	36
FosA5 (FosKP96)	*E. coli*	*K. pneumoniae*	AJE60855.1	12, 14
FosA6	*E. coli*	*K. pneumoniae*	AMQ12811.1	11
FosA7	*S. enterica* serovar Heidelberg	*S. enterica* serovar Heidelberg	KKE03230.1	27

a FosA2 and FosA7 were reported as chromosomal FosA of *E. cloacae* and *S. enterica* serovar Heidelberg but are included here for reference.

We propose that this numbering scheme (FosA followed by a number) be reserved for only acquired FosA. This would presumably include the following two scenarios: (i) a new *fosA* allele is conclusively identified on a plasmid or (ii) a new *fosA* allele is identified on the chromosome but is distinct from the intrinsic *fosA* carried by the species of interest. Given that FosA3 is by far the most common acquired FosA and yet only one variant (FosA4) has been identified, it seems reasonable to continue with the sequential numbers (e.g., FosA8, FosA9, and so forth) for acquired FosA proteins that are identified in the future. We hope that the phylogenetic analysis presented here will prove useful for the research community in determining whether a FosA enzyme of interest is intrinsic or acquired.

Our genomic survey is limited by the availability of genome sequences in public databases, which is limited for some species like *P. mirabilis* and *E. aerogenes* compared to *E. coli*. Therefore, the prevalence and diversity of FosA homologues are likely to change as additional genome sequences become available. For some species with low numbers of published genomes, the point estimate may not be accurate and prevalence may be underestimated. Also, isolation dates were not available from the NCBI genome database, making it difficult to assess temporal changes in the prevalence of FosA homologues in a given species. Finally, a vast majority of genomes were draft sequences where it was difficult to discriminate FosA homologues located on plasmids from chromosomal FosA sequences. Despite these limitations, the genomic survey provided a rapid, high-throughput assessment of the prevalence and diversity of FosA homologues in clinically relevant Gram-negative species.

In conclusion, *fosA* homologues are widely distributed among Gram-negative bacteria and encode functional FosA enzymes that inactivate fosfomycin. Given the ubiquity of glutathione in Gram-negative bacteria and the broad distribution of *fosA*, whether the primary function of FosA is fosfomycin resistance is yet to be determined. Nonetheless, the FosA homologues represent a vast reservoir of fosfomycin resistance determinants that may be mobilized to non-FosA-producing species such as *E. coli* as fosfomycin use increases in the clinic. The findings also suggest that inhibition of FosA activity may provide a viable strategy to expand the activity of fosfomycin beyond *E. coli* to include XDR Gram-negative bacteria such as *Klebsiella* and *Enterobacter* spp. producing KPC-type carbapenemase.

## MATERIALS AND METHODS

### Selection of species.

The following Gram-negative species were included in the bioinformatic analysis: *A. xylosoxidans*, *A. baumannii*, *A. pittii*, *B. cepacia*, *C. freundii*, *E. aerogenes*, *E. cloacae*, *E. coli*, *K. oxytoca*, *K. pneumoniae*, *M. morganii*, *P. mirabilis*, *P. rettgeri*, *P. stuartii*, *P. aeruginosa*, *S. marcescens*, *S. enterica*, and *S. maltophilia*. All genomes from these 18 species or species complexes that were available in the NCBI Genome database (https://www.ncbi.nlm.nih.gov/genome/) as of February 2017 were downloaded from the NCBI ftp web server using a custom shell script.

### Identification of FosA homologues.

The genomes from the above species were initially queried for FosA homologues using the most commonly observed plasmid-mediated *fosA* gene, *fosA3*, as the reference. Amino acid sequences of 71 representative FosA homologues downloaded from GenBank and from the ResFinder database (28) were used to query a database of 18,130 publicly available genomes in an iterative manner. In the first iteration, the 71 representative FosA amino acid sequences were used to query 18,130 publicly available genomes using tBLASTn with a cutoff of 40% amino acid sequence similarity and 40% query coverage and a minimum sequence length of 70 amino acids. FosA sequences identified from the first iteration were then used to query the entire genome collection a second time to identify FosA homologues that may have been missed by the first round. Identified FosA homologues were aligned and visually inspected to exclude putative homologues with very large alignment gaps. The vast majority of queried genomes represented draft sequences for which it could not be determined whether FosA homologues were chromosomal or located on a plasmid.

### Phylogenetic analysis.

One representative amino acid sequence was selected for each species using ClustalW. A global phylogeny of FosA homologues was generated by aligning all alleles found in each species using the sequence alignment tool MAFFT v7 (29), and an unrooted maximum likelihood phylogenetic tree was reconstructed using the WAG model of evolution with uniform rates of substitution on MEGA v7 (30, 31). Phylogenetic trees were visualized alongside bar graphs of allele frequencies using the interactive web platform iTOL (32). Pairwise similarity between sequences was generated using the p-distance algorithm on MEGA v7. All phylogenetic analyses and designations were based on amino acid sequences.

### Cloning of *fosA* from various species.

The *fosA* genes found in more than 80% of the genomes in a given species for which more than 10 genomes were publicly available were investigated for their functionality. These included *K. pneumoniae*, *K. oxytoca*, *E. cloacae*, *E. aerogenes*, *S. marcescens*, *M. morganii*, *P. stuartii*, and *P. aeruginosa*. The accession numbers of *fosA* genes representing each species were YP_005224903.1, WP_047724618.1, AIX57742.1, YP_004592226.1, WP_025303168.1, WP_004238530.1, WP_014658192.1, and NP_249820.1, respectively, and the genes were synthesized and cloned into vector pUC57 by GenScript (Piscataway, NJ, USA). Sequences were confirmed by Sanger sequencing. The recombinant plasmids were introduced into *E. coli* TOP10 (Thermo Scientific) via electroporation, and the transformants were selected on Mueller-Hinton agar plates containing 100 μg/ml of ampicillin.

### In-frame deletion of chromosomal *fosA*.

Directed deletion of the *fosA* open reading frame in *S. marcescens* strain K904 was achieved by two-step allelic replacement using allelic replacement vector pMQ460 (33). To target *fosA*, a 511-bp amplicon upstream of *fosA* and a 525-bp amplicon downstream of *fosA* were cloned using yeast homologous recombination (34) to generate pMQ656. Primers to generate these amplicons were as follows (5′ to 3′; lowercase letters signify DNA to target homologous recombination to pMQ460): cggccagtgccaagcttgcatgcctgcaggtcgactctGGAGAAACTCTTACCAATCACC and CGGCAGCGTCGCCGGGGCGTTTCACATGCGTGCGTTTCCTGGGCGCTAAACAGAGG, and CCTCTGTTTAGCGCCCAGGAAACGCACGCatgtgaAACGCCCCGGCGACGCTGCCG and agcggataacaatttcacacaggaaacagctatgaCTCGTGATAATGACGGCCGTCGCTG. The *fosA* deletion allele plasmid was verified by Sanger sequencing. The pMQ656 plasmid was introduced into *S. marcescens* K904 by conjugation, and *fosA* mutations were enriched for by expression of the I-SceI meganuclease from pMQ337 (35). Mutations were verified using PCR primers outside the cloned *fosA* region on pMQ656 (5′ to 3′; CAGCCTCCGCCAACGACAGCTCTG and GTGATAACATGCGCGATAGATTACC), and pMQ337 was lost from the resulting K904 Δ*fosA* strain by passage without antibiotic selection.

### Susceptibility testing.

MICs of fosfomycin were determined by the agar dilution method according to Clinical and Laboratory Standards Institute (CLSI) guidelines, with Mueller-Hinton agar supplemented with 25 μg/ml of glucose-6-phosphate (G6P) (18). *E. coli* ATCC 25922 was used as the quality control strain. Contribution of *fosA* to fosfomycin susceptibility was examined by disk diffusion testing using fosfomycin disks containing 200 μg of fosfomycin and 50 μg of G6P with or without the addition of 1 mg of sodium phosphonoformate, which is a known inhibitor of glutathione *S*-transferase (11, 36).
