# Supplementary material for: Widespread Fosfomycin Resistance in Gram-Negative Bacteria Attributable to the Chromosomal fosA Gene
Source: mBio. 2017 Aug 29;8(4):e00749-17. doi: 10.1128/mBio.00749-17 (PMC5574708; doi:10.1128/mBio.00749-17)
Supplement: TABLE S1 [file mbo004173458st1.pdf]

Supplementary Table. Genome data submitted by year groups.

Year submitted	Total genomes	*fosA*-containing genomes	%
**2002-2011**	398	77	19.3
**2012-2014**	5546	1489	26.8
**2015-2017**	12184	4084	33.5
**missing data**	2	-	
**Total**	18130	5650	31.2
