# Supplementary figures and images for: Widespread Fosfomycin Resistance in Gram-Negative Bacteria Attributable to the Chromosomal fosA Gene
Source: mBio. 2017 Aug 29;8(4):e00749-17. doi: 10.1128/mBio.00749-17 (PMC5574708; doi:10.1128/mBio.00749-17)

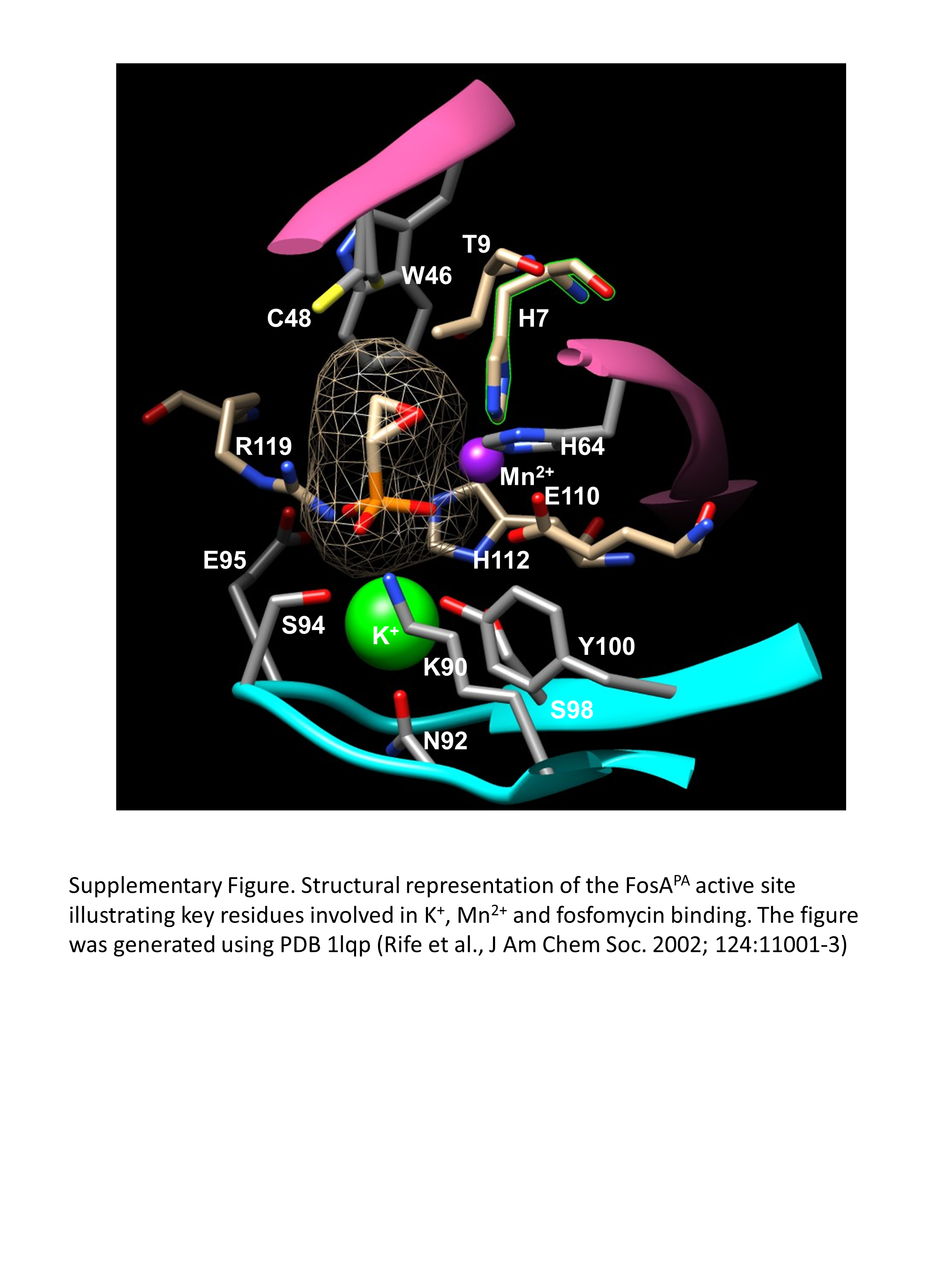

Supplement: FIG S1 [file mbo004173458sf1.tif]
