# Supplementary material for: Widespread Fosfomycin Resistance in Gram-Negative Bacteria Attributable to the Chromosomal fosA Gene
Source: mBio. 2017 Aug 29;8(4):e00749-17. doi: 10.1128/mBio.00749-17 (PMC5574708; doi:10.1128/mBio.00749-17)
Supplement: DATA SET S1 [file mbo004173458sd1.pdf]

>fosA_1_NZ_ACWU01000146
MLTGLNHLTLAVADLPASIAFYRDLLGFRLEARWDQGAYLELGSLWLCLSREPQYGGPAADYTHYAFGIAAAD
FARFAAQLRAHGVREWKQNRSEGDSFYFLDPDGHRLEAHVGDLRSRLAACRQAPYAGMRFAD
>fosA_2_AGDM01000012
MLSGLNHLTLAVSSLAPSVAFYHQLLGMTLHARWDGGAYLSCGDLWLCLSLDPQRRVTPPEESDYTHYAFSVA
EADFAGFSARLETAGVAVWKVNRSEGASYYFLDPDGHKLELHVGSLALRLAACREQPYKGMVFYDE
>fosA_3_NZ_ACWO01000079
MLSGLNHLTLAVSQLAPSVAFYQQLLGMTLHARWDSGAYLSCGDLWLCLSLDPQRRVTPPEESDYTHYAFSIS
EADFASFAARLEAAGVAIWKLNRSEGASHYFLDPDGHKLELHVGSLAQRLAACREQPYKGMVFFAE
>fosA_4_NZ_AFHW01000027
LKATGFNHVTIRVSDLSRSLLFYESLLGMKLVHRGRLDVYLEWGSAWICLIERSCESSEKPSYGVDHIAFSIT
EEDFHDAAVKLQGSGVPIVRGPLERGGGYSINFLDPDGTELELFTGSLAERMKGWS
>fosA_5_NZ_AEXB01000013
MLQSLNHLTLAVSDLQKSVTFWHELLGLALYARWNTGAYLTCGDLWVCLSYDEARQYVPPQESDYTHYAFTVA
EEDFEPFSQRLEQAGVTVWKQNKSEGASFYFLDPDGHKLELHVGSLAARLAACREKPYAGMVFTSDEA
>fosA_6_NZ_DS999363
VILGLNHITIAVSDLERSLKFYRETLGFTAHAKWDNGAYLSVGELWFCLSHDEPCPKTDYTHVAFDIEPKEFE
AFAKRVVSLGVEVWKQNKSEGQSLYILDPDGHKLEIHSGSLKSRLESLRTKLYSGLVWL
>fosA_7_NZ_AFBO01000747
MLSGLNHLTLAVSQLAPSVAFYQQLLGMTLHARWDSGAYLSCGDLWLCLSLDPQRRVTPPDESDYTHYAFSIS
EADFASFAVRLEAAGVAMWKLNRSEGASHYFLDPDGHKLELHVGSLAQRLAACREQPYKGMVFFDQ
>fosA_8_ACHE01000077
MEITSVNHICFSVSDLNTSIQFYKDILHGDLLVSGRTTAYLTIGHTWIALNQEKNIPRNEISHSYTHIAFSID
EEDFQQWIQWLKENQVNILKGRPRDIKDKKSIYFTDPDGHKIELHTGTLKDRMEYYKCENTHMQFYDEF
>fosA_9_NZ_ACZD01000244
MLSGLNHLTLAVSQLAPSVAFYQQLLGMTLHARWDSGAYLSCGDLWLCLSLDPQRRVTPPEESDYTHYAFSIS
EADFASFAARLEAAGVAIWKLNRSEGASHYFLDPDGHKLELHVGSLAQRLAACREQPYKGMVFFDQ
>fosA_10_EU195449
MLSGLNHLTLAVSQLAPSVAFYQQLLGMMLHARWDSGAYLSCGDLWLCLSLDPQRRVTPPEESDYTHYAFSIS
EADFASFAARLEAAGVAVWKLNRSEGASHYFLDPDGHKLELHVGSLAQRLAACREQPYKGMVFFAE
>fosA_11_DQ396803
MKYLLLVSHGDFSSGLKQTLGMFAGDDAIGSVIAVGLKPDEAASTFGTRFEALLKTLPEDASFVVLADIVGGS
PLTTVCNILNDHGKLQDTLVLGGMNFPMALTTLMSKDSLDNSALKEKAFSEATAAIKEFQTTSSEASDDDDI
>fosA_12_AY692231
MVRTYENAQELKEEISAAFRKYIAEFDDIPEALKDKRIDEVERTPAENLAYQVGWTTLLLQWEDRERRGLPVR
TPSDEFKWNQLGKLYRWFNDTYAHLSLRELEGMLTDNVDAIYMMIDAMSEDELFKPHMRQWADDATKTAVWEV
YRFIHVNTVAPFGSFRTKIRKWKRMAL
>fosA_13_EU487198
MLQSLNHLTLAVSDLQKSVTFWHELLGLTLHARWNTGAYLTCGDLWVCLSYDEARGYVPPQESDYTHYAFTVA
AEDFEPFSHKLEQAGVTVWKQNKSEGASFYFLDPDGHKLELHVGSLAARLAACREKPYAGMVFTSDEA
>fosA_14_AB522970
MLQGLNHLTLAVSDLASSLAFYQQLPGMRLHASWDSGAYLSCGALWLCLSLDEQRRKTPPQESDYTHYAFSVA
EEEFAGVVALLAQAGAEVWKDNRSEGASYYFLDPDGHKLELHVGNLAQRLAACRERPYKGMVFFD
>fosA_15_CP002889
VKAAFRRLSENGENYSVPPLRRQPPAVYGEHGVVYVHEVTVPRLAQKTKGHVMLQSLNHLTLAVSDLQKSVTF
WHELLGLTLHARWNTGAYLTCGDLWVCLSYDEARGYVPPQESDYTHYAFTVAAEDFEPFSHKLEQAGVTVWKQ
NKSEGASFYFLDPDGHKLELHVGSLAARLAACREKPYAGMVFTSDEA
>fosA_16_M85195
MLQSLNHLTLAVSDLQKSVTFWHELLGLTLHARWNTGAYLTCGDLWVCLSYDEARQYVPPQESDYTHYAFTVA
EEDFEPLSQRLEQAGVTIWKQNKSEGASFYFLDPDGHKLELHVGSLAARLAACREKPYAGMVFTSDEA
>FosAMM
MLTGMNHLTLAVADLDRSLHFYRDILKMTLHTRWKYGAYLTCGELWICLSADPEIIHRPIHQGYTHYAFTLPP
EQFPAFRSLLAAHQITLWKRNRSEGDSVYFLDPDGHQLEAHSGGIQQRLDACREAPYEEMIFPAPGQINV
>FosASR

MLVGINHLTIAVTDVEKSIFFYQSLLGMKLHASWKNGAYISCGDLWLCLSLDKTRLSFSHTETDYTHYAFTVS
EADFPICVAKLKQANVIVWKENKSEGKSFYFLDPDGHKLELHVGGLLQRLKSCQKAPYEGMKFY
>FosAPS
MLIGINHLTLAVSDIQKSISFYQTVLGMQLHASWERGAYLTCGALWVCLSYDPMRQEVAAAKRRDYTHYAFTV
LAEDFSDIVEKLHQAGVTVWKDNRSEGDSFYFLDPDGHQLEIHVGSLIDRLKSCRKKPYDGMVFYS
>FosASM
MLTGLNHLTLAVSDLDRSFDFYRHLLGFTPHARWQGGAYLSLGALWLCLSLDEARMQPRERDYTHYAFSVAPE
HIERVSERLRQAGVEEWKSNRSEGESLYFLDPDGHQLEIHAGDLASRLAACREKPYQGMVFY
>FosAEC
MLQSLNHLTLAVSDLQKSVTFWHELLGLALHARWNTGAYLTCGDLWVCLSYDEARQYVPPQESDYTHYAFTVA
EEDFEPFSHRLEQAGVTVWKQNKSEGASFYFLDPDGHKLELHVGSLAARLAACREKPYAGMVFTSDGA
>FosAKO
MLSGLNHLTLAVSQLAPSVAFYQQLPGMTLRARWDNGAYFSCGDLWLCLSLDPQRRVISPEESDYTHYAFSIA
EADFALFAGRLNAAGVPVWKTNKSEGASHYFLDPDGHKLELHVGSLAQRLAACRAKPYKGMVFFGEDEQAAGN
P
>FosAKP
MLSGLNHLTLAVSQLAPSVAFYQQLLGMTLHARWDSGAYLSCGDLWLCLSLDPQRRVTPPEESDYTHYAFSIS
EADFASFAARLEAAGVAVWKLNRSEGASHYFLDPDGHKLELHVGSLAQRLAACREQPYKGMVFFEQ
>FosAEA
MLSGLNHLTLAVSQLAPSVAFYHQLLGMTLHARWDSGAYLSCGDLWLCLSLDPQRRVTPPEESDYTHYAFSIS
EADFASFAARLEVAGVAVWKLNRSEGESHYFLDPDGHKLELHVGNLAQRLAACREQPYKGMVFFGE
>GCA_001039365
VKAAFRRLSENGENYSVPPLRRQPPAVYGEHGVVYVHLFTVPRPARKTKGHVMLQSLNHLTLAVSDLQKSVTF
WHELLGLALHARWNTGAYLTCGDLWVCLSYDEARQYVPPQESDYTHYAFTVAEADFEPFSQRLEQAGVTVWKQ
NKSEGASFYFLDPDGHKLELHVGSLAARLAACREKPYAGMVFSSDEA
>GCA_001375695
VKAAFRRLSENGENYSVPPLRRQPPAVYGEHGVVYVHLFTVPRPARKTKGHVMLQSLNHLTLAVSDLQKSVTF
WHELLGLTLHARWNTGAYLTCGDLWVCLSYDEARRYVPPQESDYTHYAFTVAEADFEPFSHRLEQAGVTVWKQ
NKSEGASFYFLDPDGHKLELHVGSLAARLAACREKPYSGMVFASDEA
>GCA_900077785
VKAAFRRLSENEENNSVPPLRRQPPAVYGEHGVVYVHLFTVPCPARKTKGHVMLQSLNHLTLAVSDLQKSVTF
WHELLGLTLHARWNTGAYLTCGDLWVCLSYDEARRNVPPQESDYTHYAFTVAEADFEPFSQRLEQAGVTVWKQ
NKSEGASFYFLDPDGHKLELHVGSLAARLAACREKPYAGMVFTSDGA
>GCA_900075815
VKAAFRRLSENGENYSVPPLRRLPPAVYGEDGVVYVHLFTVPRPARKTKGHVMLQSLNHLTLAVSDLEKSISF
WHELLGLTLHARWNTGAYLTCGDLWVCLSYDEARRYVPPQESDYTHYAFTVAEADFEPFSQRLEQAGVTVWKQ
NKSEGASFYFLDPDGHKLELHVGSLAARLAACREKPYAGMVFTSDEA
>GCA_900075805
VKAAFRRLSENGENYSVPPLRRQPPAVYGEHGVVYVHLFTVPRPARKTKGHVMLQSLNHLTLAVSDLQKSVTF
WHELLGLTLHARWNTGAYLTCGDLWVCLSYDEARQYVPPQESDYTHYAFTVAEEDFEPLSQRLEQAGVTVWKQ
NKSEGASFYFLDPDGHKLELHVGSLAARLAACREKPYAGMVFTSDGA
>GCA_900075305
VKAAFRRLSENGENYSVPPLRRQPPAVYGEHGVVYVHLFTVPRPARKTKGHVMLQSLNHLTLAVSDLQKSVTF
WHELLGLALHARWNTGAYLTCGDLWVCLSYVEARQYVPPQESDYTHYAFTVAEADFEPLSQRLEQAGVTVWKQ
NKSEGASFYFLDPDGHKLELHVGSLAARLAACREKPYAGMVFTSDGA
>GCA_000534055
VKAAFRRLSENGENYSEPPLRRQPPAVYGEHGVVYVHAFTVSRPARKTKGHVMLQSLNHLTLAVSDLQKSVTF
WHELLGLALHARWNTGAYLTCGDLWVCLSYDEARQYVPPQESDYTHYAFTVAEEDFEPFSQRLEQAGVTVWKQ
NKSEGASFYFLDPDGHKLELHVGSLAARLAACREKPYAGMVFTSDEA
>GCA_900077675
VKAAFRRLSENGENYSVPPLRRQPPAVYGEHGVVYVHLFTVSRPARKTKGLIMLQSLNHLTLAVSDLQKSVTF
WHELLGLTLHARWNTGAYLTCGDLWVCLSYDEARQYVPPQESDYTHYAFTVAEAEFEPFSNKLEQAGVIVWKQ
NKSEGASFYFLDPDGHKLELHVGSLAARLAACREKPYAGMVFTSDEA
>GCA_001054625

VKAAFRRLSENGENYSVPPLRRQPPAVYGEHGVVYVHLFTVPRPARKTKGHVMLQSLNHLTLAVSDLQKSVTF
WHELLGLTLHARWNTGAYLTCGDLWVCLSYDEARRYVPPQESDYTHYAFTVAEADFEPFSQKLEQAGVTVWKQ
NKSEGASFYFLDPDGHKLELHVGSLAARLAACREKPYAGMVFTSNEA
>GCA_900075035
VKAAFRRLSENGENYSVPPLRRQPPAVYGEHGVVYVHLFTVPRPARKTKGHVMLQSLNHLTLAVSDLQKSVTF
WHELLGLALHARWNTGAYLTCGDLWVCLSYDEARQYVPPQESDYTHYAFTVAEADFEPLSQRLEQAGVTVWKQ
NKSEGASFYFLDPDGHKLELHVGSLAARLAACREKPYAGMVFTSDGA
>GCA_000235765
VKAAFRRLSENGENYSVPPLRRQPPAVYGEHGVVYVHLFTVPRPAKTKGHVMLQSLNHLTLAVSDLQKSVTFW
HELLGLALHARWNTGAYLTCGDLWVCLSYDEARQYVPPQESDYTHYAFTVSEEDFEPFSQRLEQAGVTVWKQN
KSEGASFYFLDPDGHKLELHVGSLAARLAACREKPYAGMVFTSDEA
>GCA_000210775
VKAAFRRLSENEENYSVPPLRRQPPAVYGEHGVVYVHLFTVPCPARKTKGHVMLQSLNHLTLAVSDLQKSVTF
WHELLGLALHARWNTGAYLTCGDLWVCLSYDEARQYVPPQESDYTHYAFTVAEEDFEPFSQRLKQAGVTVWKQ
NKSEGASFYFLDPDGHKLELHVGSLAARLAACREKPYAGMVFTSDEA
>GCA_001052195
VKAAFRRLSENGENYSVPPLRRQPPAVYGEDGVVYVHAFTVPRPARKTKGHVMLQSLNHLTLAVSDLQKSVTF
WHELLGLALHARWNTGAYLTCGDLWVCLSYDEARRYVPPQESDYTHYAFTVAEEDFESFSQRLEQAGVTVWKQ
NKSEGASFYFLDPDGHKLELHVGSLAARLAACHEKPYAGMVFTSDEA
>GCA_900076085
VKAAFRRLSENGENYSVPPLRRQPPAVYGEHGVVYVHAFTVPRPARKTKGHVMLQSLNHLTLAVSDLQKSVTF
WHELLGLTLHARWNTGAYLTCGDLWVCLSYDEARQYVPPQESDYTHYAFTVAEADFEPFSHRLEQAGVTVWKQ
NKSEGASFYFLDPDGHKLELHVGSLAARLAACREKPYAGMVFTSDEA
>GCA_900076575
VKAAFRRLSENGENYSVPPLRRQPPAVYGEHGVVYVHEVTVPRLAQKTKGHVMLQSLNHLTLAVSDLQKSVTF
WHELLGLTLHARWNTGAYLTCGDLWVCLSYDEARQYVPPQESDYTHYAFTVAEEDFEPFSHKLEQAGVTVWKQ
NKSEGASFYFLDPDGHKLELHVGSLAARLAACREKPYAGMVFTSDEA
>GCA_001029645
VKAAFRRLSENGENYSVPPLRRQPPAVYGEHGVVYVHVTVPRLAQKTKGHVMLQSLNHLTLAVSDLQKSVTFW
HELLGLTLHARWNTGAYLTCGDLWVCLSYDEARQYVPPQESDYTHYAFTVAAEDFEPFSHKLEQAGVTVWKQN
KSEGASFYFLDPDGHKLELHVGSLAARLAACREKPYAGMVFTSDEA
>GCA_000958885
VKAAFRRLSENGENYSVPPLRRLPPAVYGEHGVVYVHLFTVSRPARKTKGHVMLQSFNHLTLAVSDLQKSVTF
WHELLGLTLHARWNTGAYLTCGDLWVCLSYDEARRYVPPQESDYTHYAFTVAEEDFEPFSQRLEQAGVTVWKQ
NKSEGASFYFLDPDGHKLELHVGSLAARLAACREKPYAGMVFTSDEA
>GCA_900075695
VKAAFRRLSENGENYSVPPLRRQPPAVYGEHGVVYVHLFTVSRPARKTKGHVMLQSLNHLTLAVSDLQKSVTF
WHELLGLELHARWNTGAYLTCGELWVCLSYDEARRYVPPQESDYTHYAFTVAEEDFEPFSHRLEQAGVTVWKQ
NKSEGASFYFLDPDGHKLELHVGSLAARLAACREKPYSGMVFASDEA
>GCA_900075045
VKAAFRRLSENGENYSVPPLRRQPPAVYGEHGVVYVHLFTVPRPARKTKGHVMLQSLNHLTLAVSDLQKSVTF
WHELLGLTLHARWNTGAYLTCGDLWVCLSYDEARQYVPPQKSDYTHYAFTVAEEDFEPFSHRLEQAGVTVWKQ
NKSEGASFYFLDPDGHKLELHVGSLAARLAACREKPYAGMVFTSDEA
>GCA_900075395
VKAAFRRLSENGENYSVPPLRRQPPAVYGEHGVVYVHLFTVSRPARKTKGHVMLQSLNHLTLAVSDLQKSVSF
WHELLGLTLHARWNTGAYLTCGDLWVCLSYDEARQYVPPQESDYTHYAFTVAEEDFEPFSHRLEQAGVTVWKQ
NKSEGASFYFLDPDGHKLELHVGSLAARLAACREKPYAGMVFTSDGA
>GCA_000952375
VKAAFRRLSENGENYSEPPLRRQPPAVYGEHGVVYVHLFTVSRPARKTKGLIMLQSLNHLTLAVSDLQKSITF
WHELLGLTLHARWNTGAYLTCGDLWVCLSYDEARQYVPPQESDYTHYAFTVAEADFEPFSHRLEQAGVTVWKQ
NKSEGASFYFLDPDGHKLELHVGSLAARLAACREKPYAGMVFTSDEA
>GCA_001190095

VKAAFRRLSENGENYSVPPLRRQPPAVYGEHGVVYVHLFTVSRPARKTKGHVMLQSLNHLTLAVSDLQKSVTF
WHELLGLALHARWNTGAYLTCGDLWVCLSYDEARRYVPPQESDYTHYAFTVSEADFEPFSQRLEQAGVTVWKQ
NKSEGASFYFLDPDGHKLELHVGSLAARLAACREKPYAGMVFTSDEA
>GCA_001052225
VKAAFRRLSENGENYSVPPLRRQPPAVYGEDGVVYVHLFTVPRPARKTKGHVMLQSLNHLTLAVSDLQKSVTF
WHELLGLALHARWNTGAYLTCGELWVCLSYDEARRYVPPQESDYTHYAFTVAEEDFEPFSHRLEQAGVTVWKQ
NKSEGASFYFLDPDGHKLELHVGSLAARLAACREKPYSGMVFASDEA
>GCA_900077845
VKAAFRRLSENGENYSVPPLRRQPPAVYGEDGVVYVHLFTVSRPARKTKGHVMLQSLNHLTLAVSDLQKSVTF
WHELLGLTLHARWNTGAYLTCGDLWVCLSYDEARRYVPPQESDYTHYAFTVTEEDFEPFSHRLEQAGVTVWKQ
NKSEGASYYFLDPDGHKLELHVGSLAARLAACREKPYAGMVFTSDEA
>GCA_000783675
VKAAFRRLSENGENYSVPPLRRQPPAVYGEHGVVYVHLFTVPRPARKTKGHVMLQSLNHLTLAVSDLQKSVTF
WHELLGLALHARWNTGAYLTCGDLWVCLSYDEARQYVPPQESDYTHYAFTVAEADFEPFSQRLEQAGVTVWKQ
NKSEGASFYFLDPDGHKLELHVGSLAARLAACREKPYAGMVFASDEA
>GCA_000812505
VKAAFRRLSENGENYSVPPLRRQPPAVYGEHGVVYVHAFTVSRPAQKTKGHVMLQSLNHLTLAVSDLQKSVTF
WHELLGLALHARWNTGAYLTCGDLWVCLSYDEARRNVPPQESDYTHYAFTVAEEDFEPFSHRLEQAGVTVWKQ
NKSEGASFYFLDPDGHKLELHVGSLAARLAACREKPYAGMVFTSDGA
>GCA_900076485
VKAAFRRLSENGENYSVPPLRRQPPAVYGEHGVVYVHLFTVSRPARKTKGHVMLQSLNHLTLAVSDLQKSVTF
WHALLGLTLHARWNTGAYLTCGELWVCLSYDEARRYVPPQESDYTHYAFTVAEEDFEPFSQRLEQAGVTVWKQ
NKSEGASFYFLDPDGHKLELHVGSLAARLAACREKPYAGMVFTSDEA
>GCA_900078045
VKAAFRRLSENGENYSVPPLRRQPPAVYGEHGVIYVHAFTVPRPARKTKGHVMLQSLNHLTLAVSDLQKSVTF
WHELLGLTLHARWNTGAYLTCGDLWVCLSYDEARQYVPPQESDYTHYAFTVAEADFEPFSHRLEQAGVTVWKQ
NKSEGASFYFLDPDGHKLELHVGSLAARLAACREKPYAGMVFTSDEA
>GCA_900075595
VKAAFRRLSENGENYSVPPLRRQPPAVYGEHGVVYVHLFTVPRPARKTKGHVMLQSLNHLTLAVSDLQKSVTF
WHELLGLALHARWNTGAYLTCGDLWVCLSYDEARQYVPPQESDYTHYAFTVAEEDFEPLSQRLEQAGVTVWKQ
NKSEGASFYFLDPDGHKLELHVGSLAARLAACREKPYAGMVFTSDGA
>GCA_000534215
VKAAFRRLSENEENYSVPPLRRQPPAVYGEHGVVYVHLFTVPCPARKTKGHVMLQSLNHLTLAVSDLQKSVTF
WHELLGLALHARWNTGAYLTCGDLWVCLSYDEARGNVPPQESDYTHYAFTVAEADFEPFSQRLEQAGVTVWKQ
NKSEGASFYFLDPDGHKLELHVGSLAARLAACREKPYAGMVFTSDEA
>GCA_001653545
VKAAFRRLSENEENNSGPPLRRQPPAVYGEHGVVYVHLFTVPCPARKTKGHVMLQSLNHLTLAVSDLQKSVTF
WHELLGLALHARWNTGAYLTCGDLWVCLSYDEARQYVPPQESDYTHYAFTVAEEDFEPFSQRLKQAGVTVWKQ
NKSEGASFYFLDPDGHKLELHVGSLAARLAACREKPYAGMVFTSDEA
>GCA_900075935
VKAAFRRLSENGENYSVPPLRRQPPAVYGEHGVVYVHEVTVPRLAQKTKGHVMLQSLNHLTLAVSDLQKSVTF
WHELLGLTLHARWNTGAYLTCGDLWVCLSYDEARQYVPPQESDYTHYAFTVAAEDFEPFSHKLEQAGVTVWKQ
NKSEGASFYFLDPDGHKLELHVGSLAARLAACREKPYAGMVFTSDEA
>GCA_001055075
VKAAFRRLSENGENYSVPPLRRQPPAVYGEHGVVYVHLFTVPRPARKTKGHVMLQSLNHLTLAVSDLQKSVTF
WHELLGLALHARWNTGAYLTCRDLWVCLSYDEARQYVPPQESDYTHYAFTVAEEDFDAFSRRLEQAGVTVWKQ
NKSEGASFYFLDPDGHKLELHVGSLAARLAACREKPYAGMVFTSDEA
>GCA_900076725
VKAAFRRLSENGENYSVPPLRRQPPAVYGEDGVVYVHAFTVPRPARKTKGHVMLQSLNHLTIAVSDLQKSVTF
WHALLGLTLHARWNTGAYLTCGELWVCLSYDEARRYVPPQESDYTHYAFTVAEEDFEPFSHRLEQAGVTVWKQ
NKSEGASFYFLDPDGHKLELHVGSLAARLAACREKPYAGMVFTSDEA
>GCA_000747015

```
VKAAFRRLSENEENYSVPPLRRQPPAVYGEHGVVYVHLFTVPCPARKTKGHVMLQSLNHLTLAVSDLQKSVTF
WHELLGLALHARWNTGAYLTCGDLWVCLSYDEARRYVPPQESDYTHYAFTVAEADFEPFSQRLEQAGVTVWKQ
NKSEGASFYFLDPDGHKLELHVGSLAARLAACREKPYAGMVFTSDKA
>GCA_000422225
VKAAFRRLSENGENYSVPPLRRQPPAVYGEHGVVYVHEVTVPRLAQKTKGHVMLQSLNHLTLAVSDLQKSVTF
WHELLGLTLHSRWNTGAYLTCGDLWVCLSYDEARGYVPPQESDYTHYAFTVAAEDFEPFSHKLEQAGVTVWKQ
NKSEGASFYFLDPDGHKLELHVGSLAARLAACREKPYAGMVFTSDEA
>GCA_900075095
VKAAFRRLSENEENNSGPPLRRQPPAVYGEDGVVYVHLFTVPCPARKTKGHVMLQSLNHLTLAVSDLQKSVTF
WHELLGLTLHARWNTGAYLTCGDLWVCLSYDEARQYVPPQESDYTHYAFTVAEEDFEPFSQRLEQAGVTVWKQ
NKSEGASFYFLDPDGHKLELHVGSLAARLAACREKPYAGMVFTSDEA
>GCA_900077535
VKAAFRRLSENGENYSVPPLRRQPPAVYGEHGVVYVHLFTVSRPARKTKGLIMLQSLNHLTLAVSDLQKSVTF
WHELLGLTLHARWNTGAYLTCGDLWVCLSYDEARQYVPPQEIDYTHYAFTVAEADFEPFSNKLEQAGVTVWKQ
NKSEGASFYFLDPDGHKLELHVGSLAARLAACREKPYAGMVFTSDEA
>GCA_001054505
VKAAFRRLSENGKNYSVPPLRRQPPAVYGEHGVVYVHLFTVSRPARKTKGHVMLLSLNHLTLAVSDLQKSVTF
WHELLGLTLHARWNTGAYLTCGDLWVCLSYDEARRYAPPQESDYTHYAFTVAEADFEPFSQRLEQAGVTVWKQ
NKSEGASFYFLDPDGHKLELHVGSLAARLAACREKPYAGMVFTSDEA
>GCA_900075075
VKAAFRRLSENGENYSVPPLRRQPPAVYGEHGVVYVHLFTVSRPARKTKGHVMLQSLNHLTLAVSDLQKSVTF
WHELLGLTLHARWNSGAYLTCGDLWVCLSYDEARRYVPPQESDYTHYAFTVAEADFEPFSNKLEQAGVTVWKQ
NKSEGASFYFLDPDGHKLELHVGNLAARLAACREKPYAGMVFTSDEA
>GCA_001653625
VKAAFRRLSENGENYSVPPLRRQPPAVYGEHGVVYVHAFTVSRPARKTKGHVMLQSLNHLTLAVSDLQKSVTF
WHELLGLTLHARWNTGAYLTCGDLWVCLSYDEARQYVPSQESDYTHYAFTVAEEDFEPFSQRLEQAGVTVWKQ
NKSEGASFYFLDPDGHKLELHVGSLAARLAACREKPYAGMVFTSDKA
>GCA_900077545
VKAAFRRLSENGENYSVPPLRRQPPAVYGEHGVVYVHLFTVSRPARKTKGHVMLQSLNHLTLAVSDLQKSVTF
WHELLGLALHARWNTGAYLTCGDLWVCLSYDEARQYVPPQESDYTHYAFTVAEEDFEPFSQRLEQAGVTVWKQ
NKSEGASFYFLDPDGHKLELHVGSLAARLAACREKPYAGMVFTSDEA
>GCA_900075315
VKAAFRRLSENGENYSVPPLRRQPPAVYGEHGVVYVHLFTVPRPARKTKGHVMLQSLNHLTLAVSDLQKSVTF
WHELLGLALHARWNTGAYLTCGDLWVCLSYDEARRYVPPQESDYTHYAFTVAEADFEPFSQRLEQAGVTVWKQ
NKSEGASFYFLDPDGHKLELHVGSLAARLAACREKPYAGMVFTSDKA
>GCA_900075065
VKAAFRRLSENGENYSVPPLRRQPPAVYGEHGVVYVHLFTVSRPARKTKGHVMLQSLNHLTLAVSDLQKSVTF
WHELLGLTLHARWNTGAYLTCGDLWVCLSYDEARQYVPPQESDYTHYAFTVAEEDFEPFSQRLEQAGVTVWKQ
NKSEGASFYFLDPDGHKLELHVGSLAARLAACREKPYAGMVFTSDGA
>GCA_000968725
VKAAFRRLSENGENYSVPPLRRQPPAVYGEHGVVYVHLFTVPRPARKTKGHVMLQSLNHLTLAVSDLQKSVTF
WHALLGLTLHARWNTGAYLTCGDLWVCLSYDEARQYVPPQESDYTHYAFTIAEADFEPFSQRLKQAGVTVWKQ
NKSEGASYYFLDPDGHKLELHVGSLAARLAACREKPYAGMVFTSDEA
>GCA_000953895
VKAAFRRLSENGENYSVPPLRRQPPAVYGEYGVVYVHLFTVPRPARKTKGHVMLQSLNHLTLAVSDLQKSVTF
WHELLGLTLHARWNTGAYLTCGDLWVCLSYDEARRYVPPQESDYTHYAFTLAEADFEPFSQKLEQAGVTVWKQ
NKSEGASFYFLDPDGHKLELHVGSLAARLAACREKPYAGMVFTSDEA
>GCA_000952575
VKAAFRRLSENGENYSVPPLRRQPPAVYGEHGVVYVHLFTVPRPARKTKGHVMLQSLNHLTLAVSDLQKSVTF
WHELLGLTLHARWNTGAYLTCGDLWVCLSYDEARQYVPPQESDYTHYAFTVAEADFEPFSHRLEQAGVTVWKQ
NKSEGASFYFLDPDGHKLELHVGSLAARLAACREKPYAGMVFTSDEA
>GCA_000938355
```

VKAAFRRLSENGENYSVPPLRRQPPAVYGEDGVVYVHAFTVPRPARKTKGHVMLQSLNHLTIAVSDLQKSVTF
WHALLGLTLHARWNTGAYLTCGELWVCLSYDEARRYVPPQESDYTHYAFTVAEEDFEPFSQRLEQAGVTVWKQ
NKSEGASFYFLDPDGHKLELHVGSLAARLAACREKPYAGMVFTSDGA
>GCA_001055515
VKAAFRRLSENGKNYSVPPLRRQPPAVYGEDGVVYVHLFTVPRPARKTKGHVMLQSLNHLTLAVSDLQKSITF
WHALLGLTLHARWKTGAYLTCGDLWVCLSYDEARRYVPPQESDYSHYAFTVAEEDFEPFSQRLEQAGVTVWKQ
NKSEGASFYFLDPDGHKLELHVGSLAARLAACREKPYAGMVFTSDEA
>GCA_900075895
VKAAFRRLSENGKNYSVPPLRRQPPAVYGEHGVVYVHLFTVPRPARKTKGHVMLQSLNHLTLAVSDLQKSVTF
WHELLGLALHARWNTGAYLTCGDLWVCLSYDEARQYVPPQESDYTHYAFTVAEEDFEPLSERLEQAGVTVWKQ
NKSEGASFYFLDPDGHKLELHVGSLAARLAACREKPYAGMVFTSDGA
>GCA_900076205
VKAAFRRLSENEENNSGPPLRRQPPAVYGEDGVVYVHAFTVSRPARKTKGHVMLQSLNHLTLAVSDLQKSVTF
WHELLGLALHARWNTGAYLTCGDLWVCLSYDEARRYVPPQESDYTHYAFTVAEADFEPFSQRLEQAGVTVWKQ
NKSEGASFYFLDPDGHKLELHVGSLAARLAACREKPYAGMVFTSDKA
>GCA_001562175
VKAAFRRLSENEENYSVPPLRRKPPAVYGEHGVVYVHLFTVPRPARKTKGHVMLQSLNHLTLAVSDLQKSVTF
WHELLGLALHARWNTGAYLTCGDLWICLSYDEARQYVPPQESDYTHYAFTVAEEDFEPFSQRLNQAGVTVWKQ
NKSEGASFYFLDPDGHKLELHVGSLAARLAACREKPYAGMVFTSDKA
>GCA_900075995
VKAAFRRLSENEENYSVPPLRRQPPAVYGEDGVVYVHAFTVSRPARKTKGHVMLQSLNHLTLAVSDLQKSVTF
WHELLGLALHARWNTGAYLTCGDLWVCLSYDEARQYVPPQESDYTHYAFTVAEEDFEPFSQRLEQAGVTVWKQ
NKSEGASFYFLDPDGHKLELHVGSLAARLAACREKPYAGMVFTSDEA
>GCA_001054435
VKAAFRRLSENGENYSVPPLRRQPPAVYGEHGVVYVHLFTVPRPARKTKGHVMLQSLNHLTLAVSDLQKSISF
WHELLGLTLHARWNTGAYLTCGDLWICLSYDEARRYVPPQESDYTHYAFTVAEADFEPFSQRLEQAGVTVWKQ
NKSEGASFYFLDPDGHKLELHVGSLAARLAACREKPYAGMVFTSDEA
>GCA_000564975
VKAAFRRLSENGENYSVPPLRRQPPAVYGEHGVVYVHLFTVPRPARKTKGHVMLQSLNHLTLAVSDLQKSVTF
WHELLGLTLHARWNTGAYLTCGDLWVCLSYDEARQYVPPQESDYTHYAFTVAEEDFEPFSHRLEQAGVTVWKQ
NKSEGASFYFLDPDGHKLELHVGSLAARLAACREKPYAGMVFTSDEA
>GCA_001473135
VKAAFRRLSENGENYSVPPLRRQPPAVYGEHGVVYVHAFTVSRPAQKTKGHVMLQSLNHLTLAVSDLQKSVTF
WHELLGLALHARWNTGAYLTCGDLWVCLSYDEARQYVPPQESDYTHYAFTVAEEDFEPFSHRLEQAGVTVWKQ
NKSEGASFYFLDPDGHKLELHVGSLAARLAACREKPYAGMVFTSDGA
>GCA_900077855
VKAAFRRLSENGENYSVPPLRRQPPAVYGEHGVVYVHAFTVSRPAQKTKGHVMLQSLNHLTLAVSDLQKSVTF
WHELLGLALHARWNTGAYLTCGELWVCLSYDEARRYVPPQESDYTHYAFTVAEEDFEPFSHRLEQAGVTVWKQ
NKSEGASFYFLDPDGHKLELHVGSLAARLAACREKPYAGMVFTSDEA
>GCA_900078025
KAAFRRLSENGENYSVPPLRRQPPAVYGEDGVVYVHLFTVPRPARKTKGHVMLQSLNHLTLAVSDLQKSVTFW
HELLGLTLHARWNTGAYLTCGDLWVCLSYDEARRYVPPQESDYTHYAFTVAEADFEPFSQRLEQAGVTVWKQN
KSEGASFYFLDPDGHKLELHVGSLAARLAACREKPYAGMVFTSDEA
>GCA_001057545
KAAFRRLSENGENNSEPPLRRQPPAVYGEHGVVYVHGFTVSRPARKTKGLIMLQSLNHLTLAVSDLQKSITFW
HELLGLTLHARWNTGAYLTCGDLWVCLSYDEARQYVPPQESDYTHYAFTVAEADFEPFSNKLEQEGVTVWKQN
KSEGASFYFLDPDGHKLELHVGSLAARLAACREKPYAGMVFTSDEA
>GCA_000492455
KAAFRRLSENGENYSVPPLRRQPPAVYGEHGVVYVHLFTVPCPARKTKGHVMLQSLNHLTLAVSDLQKSVTFW
HELLGLTLHARWNTGAYLTCGDLWVCLSYDEARQYVPPQKSDYTHYAFTVAEADFEPFSQRLEQAGVTVWKQN
KSEGASFYFLDPDGHKLELHVGSLAARLAACREKPYAGMVFTSDEA
>GCA_000692255

KAAFRRLSENGENNSEPPLRRQPPAVYGEHDVVYVHAFTVSRPARKTKGLIMLQSLNHLTLAVSDLQKSITFW
HELLGLTLHARWNTGAYLTCGDLWVCLSYDEARQYVPPQESDYTHYAFTVAEADFEPFSNKLEQAGVTVWKQN
KSEGASFYFLDPDGHKLELHVGSLAARLAACREKPYAGMVFTSDEA
>GCA_900078075
KAAFRRLSENGENYSVPPLRRQPPAVYGEHGVVYVHLFTVPRPARKTKGHVMLQSLNHLTLAVSDLQKSVTFW
HELLGLPLHARWNTGAYLTCGDLWVCLSYDEARQYVPPQESDYTHYAFTVAEEDFEPFSHRLEQAGVTVWKQN
KSEGASFYFLDPDGHKLELHVGSLAARLAACREKPYAGMVFTSDGA
>GCA_900075575
KAAFRRLSENGKNYSVPPLRRQPPAVYGEDGVVYVHAFTVSRPARKTKGHVMLQSLNHLTLAVSDLQKSINFW
HELMGLTLHARWNTGAYLTCGDLWVCLSYDEARRYVPPQESDYTHYAFTVAEEDFEPFSQRLKQAGVTVWKQN
KSEGASFYFLDPDGHKLELHVGSLAARLAACREKPYAGMLFTSDEA
>GCA_001472955
KAAFRRLSENGENYSVPPLRRQPPAVYGEHGVVYVHAFTVSRPAQKTKGHVMLQSLNHLTLAVSDLQKSVTFW
HELLGLALHARWNTGAYLTCGDLWVCLSYDEARQYVPPQESDYTHYAFTVAEEDFEPFSHRLDQAGVTVWKQN
KSEGASFYFLDPDGHKLELHVGSLAARLAACREKPYAGMVFTSDGA
>GCA_001053395
KAAFRRLSENGENNSEPPLRRQPPAVYGEHGVVYVHLFTVSRPARKTKGLIMMQSFNHLTLAVSDLQKSITFW
HELLGLTLHARWNTGAYLTCGDLWVCLSYDEARQYVPPQESDYTHYAFTVAEADFEPFSNKLEQAGVTVWKQN
KSEGASFYFLDPDGHKLELHVGSLAARLAACREKPYAGMVFTSDEA
>GCA_000390425
KAAFRRLSENGENYSVPPLRRQPPAVYGEHGVVYVHLFNVPRPARKTKGHVMLQSLNHLTLAVSDLQKSVTFW
HELLGLALHARWNTGAYLTCGDLWVCLSYDEARRNVPPQESDYTHYAFTVAEEDFKPFSQRLEQAGVTVWKQN
KSEGASFYFLDPDGHKLELHVGSLAARLAACREKPYAGMVFTSDGA
>GCA_000818625
KAAFRRLSENGENYSVPPLRRQPPAVYGEHGVVYVHAFTVSRPAQKTKGHVMLQSLNHLTLAVSDLQKSVTFW
HELLGLALHARWNTGAYLTCGDLWVCLSYDEARQYVPPQESDYTHYAFTVAEEDFEPFSQRLEQAGVTVWKQN
KSEGASFYFLDPDGHKLELHVGSLAARLAACREKPYAGMVFTSDGA
>GCA_000958735
KAAFRRLSENGENYSVPPLRRQPPAVYGEHGVVYFHAFTVSRPARKTKGHVMLQSLNHLTLAVSDLQKSVTFW
HELLGLALHARWNTGAYLTCGDLWVCLSYDEARRYVPPQESDYTHYAFTVAEADFEPFSQRLEQAGVTVWKQN
KSEGASFYFLDPDGHKLELHVGSLAARLAACREKPYAGMVFTSDEA
>GCA_001022575
KAAFRRLSEYGENYSVPPLRRQPPAVYGEHGVVYVHLFTVPRPARKTKGHVMLQSLNHLTLAVSDLQKSVTFW
HELLGLALHARWNTGAYLTCGDLWVCLSYDDARQYVPPQESDYTHYAFTVAEADFEPFSQRLEQAGVTVWKQN
KSEGASFYFLDPDGHKLELHVGSLAARLAACREKPYAGMVFTSDEA
>GCA_000515295
KAAFRRLSENGENYSVPPLRRQPPAVYGEHGVVYVHLFTVPRPARKTKGHVMLQSLNHLTLAVSDLQKSVTFW
HELLGLALHARWNTGAYLTCGDLWVCLSYDEARQYVPPQESDYTHYAFTVAEEDFEPFSHRLEQAGVTVWKQN
KSEGASFYFLDPDGHKLELHVGSLAARLAACREKPYAGMVFTSDEA
>GCA_000692275
KAAFRRLSENEENYSVPPLRRQPPAVYGEHGVVYVHLFTVPRPARKTKGHVMLQSLNHLTLAVSDLQKSVTFW
HELLGLTLHARWNTGAYLTCGDLWVCLSYDEARQVVPPQESDYTHYAFTVTEEDFEPFSNKLEQAGATVWKQN
KSEGASFYFLDPDGHKLELHVGSLAARLAACREKPYAGMVFTSDEA
>GCA_900075115
KAAFRRLSENGENYSVPPLRRQPPAVYGEHGVVYVHLFTVPCPARKTKGHVMLQSLNHLTLAVSDLQKSVTFW
HELLGLALHARWYTGAYLTCGDLWVCLSYDEARQYVPPQESDYTHYAFTVAEEDFEPFSQRLEQAGVTVWKQN
KSEGASFYFLDPDGHKLELQVGSLAARLAACREKPYAGMVFTSDEA
>GCA_900075645
KAAFRRLSENGENYSVPPLRRQPPAVYGEHGVVYVHLFTVPCPARKTKGHVMLQSLNHLTLAVSDLQKSVTFW
HELLGLTLHARWNTGAYLTCGDLWVCLSYDEARQYVPPQESDYTHYAFTVAEEDFEPFSQRLEQAGVTVWKQN
KSEGASFYFLDPDGHKLELHVGSLAARLAACREKPYAGMVFTSDEA
>GCA_000770155

```
KAAFRRLSENGENYSVPPLRRQPPAVYGEHGVVYVHAFTVSRPARKTKGHVMLQSLNHLTLAVSDLQKSVTFW
HELLGLALHARWNTGAYLTCGDLWVCLSYDEARQYVPPQESDYTHYAFTVAEEDFEPFSHRLEQAGVIVWKQN
KSEGASFYFLDPDGHKLELHVGSLAARLAACREKPYAGMVFTSDGA
>GCA_900076025
KAAFRRLSENGENNSEPPLRRQPPAVYGEHGVVYVHLFTVSRPARKTKGLIMLQSLNHLTLAVSDLQKSITFW
HELLGLTLHARWNTGAYLTCGDLWVCLSYDEARQYVPPQESDYTHYAFTVAEADFEPFSNKLEQAGVTVWKQN
KSEGASFYFLDPDGHKLELHVGSLAARLAACREKPYAGMVFTSDEA
>GCA_900075565
KAAFRRLSENGENYSVPPLRRQPPAVYGEHGVVYVHLFTVSRPARKTKGHVMLQSLNHLTLAVSDLQKSVTFW
HELLGLALHARWNTGAYLTCGDLWVCLSYDEARQYVPPQESDYTHYAFTVAEEDFEPFSHRLEQAGVTVWKQN
KSEGASFYFLDPDGHKLELHVGSLAARLAACREKPYAGMVFTSDGA
>GCA_900076935
KAAFRRLSENGENYSVPPLRRQPPAVYGEHGVVYVHLFTVPCPARKTKGHVMLQSLNHLTLAVSDLQKSVTFW
HELLGLALHARWNTGAYLTCGDLWVCLSYDEARRYVPPQESDYTHYAFTVAEADFEPFSQRLEQAGVTVWKQN
KSEGASFYFLDPDGHKLELHVGSLAARLAACREKPYTGMVFTSDKA
>GCA_000692235
KAAFRRLSENGKNYSVPPLRRQPPAVYGKHGVVYVHAFTVSRPAQKTKGHVMLQSLNHLTLAVSDLQKSVTFW
HELLGLTLHARWNTGAYLTCGDLWVCLSYDEARRYVPPQESDYTHYAFTVAEEDFEPFSQRLEQAGVTVWKQN
KSEGASFYFLDPDGHKLELHVGSLAARLAACREKPYAGMVFTSDEA
>GCA_900077795
KAAFRRLSENGENYSVPPLRRQPPAVYGEHGVVYVHLFTVPCPARKTKGHVMLQSLNHLTLAVSDLQKSVTFW
HELLGLALHARWNTGAYLTCGDLWVCLSYDEARQYVPPQESDYTHYAFTVAEEDFEPFSQRLEQAGVTVWKQN
KSEGASFYFLDPDGHKLELHVGSLAARLAACREKPYAGMVFTSDEA
>GCA_900078065
KAAFRRLSENGEYYSVPPLRRQPPAVYGEDGVVYVHLFTVPCPARKTKGHVMLQSLNHLTLAVSDLQKSVTFW
HELLGLTLHARWNTGAYLTCGDLWVCLSYDEARQYVPPQESDYTHYAFTVAEADFEPFSQRLEQAGVTVWKQN
KSEGASFYFLDPDGHKLELHVGSLAARLAACREKPYTGMVFTSDKA
>GCA_900077155
KAAFRRLSENGENISEPPLRRQPPAVYGEHGVVYVHLFTVSRPARKTKGLIMLQSLNHLTLAVSDLQKSITFW
HELLGLTLHARWNTGAYLTCGDLWVCLSYDEARQYVPPQESDYTHYAFTVAEADFEPFSNKLEQAGVTVWKQN
KSEGASFYFLDPDGHKLELHVGSLAARLAACREKPYAGMVFTSDEA
>GCA_000025565
KAAFRRLSENGENYSVPPLRRQPPAVYGEHGVVYVHAFTVSRPAQKTKGHVMLQSLNHLTLAVSDLQKSVTFW
HELLGLALHARWNTGAYLTCGDLWVCLSYDEARRNVPPQESDYTHYAFTVAEEDFEPFSHRLEQAGVTVWKQN
KSEGESFYFLDPDGHKLELHVGSLAARLAACREKPYAGMVFTSDGA
>GCA_000534455
KAAFRRLSENGENYSVPPLRRQPPAVYGEHGVVYVHLFTVPRPARKTKGLVMLQSLNHLTLAVSDLQKSVTFW
HELLGLPLHARWNTGAYLTCGDLWVCLSYDEARRYVPPQESDYTHYAFTVTEEDFEPFSHRLEQAGVTVWKQN
KSEGASFYFLDPDGHKLELHVGSLAARLAACREKPYAGMVFTSDGA
>GCA_900076515
KAAFRRLSENGEYYSVPPLRRQPPAVYGEDGVVYVHLFTVPRPARKTKGHVMLQSLNHLALAVSDLQKSVTFW
HELLGLALHARWNTGAYLTCGDLWVCLSYDEARQYVPPQESDYTHYAFTVSEADFEPFSNKLEQAGITVWKQN
KSEGASFYFLDPDGHKLELHVGSLAARLAACREKPYAGMVFTSDEA
>GCA_000286275
KAAFRRLSENGENNSEPPLRRQPPAVYGEHGVVYVHAFTVSRPARKTKGHIMLRSLNHLTLAVSDLQKSITFW
HELLGLTLHARWNTGAYLTCGDLWVCLSYDEARQNVPPQESDYTHYAFTVAEADFEPFSNKLEQAGVTVWKQN
KSEGSSFYFLDPDGHKLELHVGSLAVRLAACREKPYAGMVFTSDEA
>GCA_001525285
KPPFGGYLKNEENYSVPPLRRQPPAVYGEHGVVYVHAFTVSRPARKTKGHVMLQSLNHLTLAVSDLQKSVTFW
HELLGLALHARWNTGAYLTCGDLWVCLSYDEARQYVPPQESDYTHYAFTVGEEDFEPFSQRLEQAGVTVWKQN
KSEGASFYFLDPDGHKLELHVGSLAARLAACREKPYAGMVFTSDGA
>GCA_900077305
```

```
KAAFRRLSENGENYSVPPLRRQPPAVYGEHGVVYVHLFTVPRPARKTKGHVMLQSLNHLTLAVSDLQKSVTFW
HELLGLTLHARWNTGAYLTCGDLWVCLSYDEARRNVPPQESDYTHYAFTVTEEDFVAFSQKLEQAGVTVWKQN
KSEGASFYFLDPDGHKLELHVGSLAARLAACREKPYAGMVFTSDEA
>GCA_001011715
KAAFRRLSENGENYSVPPLRRQPPAVYGEHGVVYVHAFTVSRPAQKTKGHVMLQSLNHLTLAVSDLQKSVTFW
HELLGLALHARWNTGAYLTCGDLWVCLSYDEARRNVPPQESDYTHYAFTVAEEDFEPFSHRLEQAGVTVWKQN
KSEGASFYFLDPDGHKLELHVGSLAARLAACREKPYAGMVFTSDGA
>GCA_001472555
KPPFGGYLKNEENYSVPPLRRQPPAVYGEHVVVYVHAFTVSRPARKTKGHVMLQSLNHLTLAVSDLQKSVTFW
HELLGLALHARWNTGAYLTCGDLWVCLSYDEARQYVPPQESDYTHYAFTVAEEDFEPFSHRLEQAGVTVWKQN
KSEGASFYFLDPDGHKLELHVGSLAARLAACREKPYAGMVFTSDGA
>GCA_001187305
KAAFRRLSENGENYSVPPLRRQPPAVYGEHGVVYVHLFTVPRPARKTKGHVMLQSLNHLTLAVSDLQKSISFW
HELLGLTLHARWNTGAYLTCGDLWVCLSYDEARRYVPPQESDYTHYAFTVAEADFEPFSHRLEQAGVTVWKQN
KSEGASFYFLDPDGHKLELHVGSLAARLAACREKPYAGMVFTSDEA
>GCA_000952315
KAAFRRLSENGKNYSVPPLRRQPPAVYGEHGVVYVHLFTVPRPARKTKGHVMLQSLNHLTLAVSDLQKSVTFW
HELLGLTLHARWNTGAYLTCGDLWVCLSYDEARRYVPPQESDYTHYAFTVAEADFEPFSQRFEQAGVTVWKQN
KSEGASFYFLDPDGHKLELHVGSLAARLAACREKPYAGMVFTSDEA
>GCA_900077215
KAAFRRLSENGENYSVPPLRRQPPAVYGEHGVVYVHLFTVSRPARKTKGHVMLQSLNHLTLAVSDLQKSVTFW
HELLGLALHARWNTGAYLTCGDLWVCLSYDEARQYVPPQESDYTHYAFTVAEADFESFSHRLEQAGVTVWKQN
KSEGASFYFLDPDGHKLELHVGSLAARLAACREKPYAGMVFTSDEA
>GCA_900050915
KAAFRRLSENVENYSVPPLRRQPPAVYGEHGVVYVHAFTVSRPAQKTKGHVMLQSLNHLTLAVSDLQKSVTFW
HELLGLALHARWNTGAYLTCGDLWVCLSYDEARRNVPPQESDYTHYAFTVAEEDFEPFSHRLEQAGVTVWKQN
KSEGASFYFLDPDGHKLELHVGSLAARLAACREKPYAGMVFTSDGA
>GCA_900075125
KAAFRRLSENGENNSVPPLRRQPPAVYGEHGVVYVHLFTVPCPARKKKGHVMLQSLNHLTLAVSDLQKSVTFW
HELLGLALHARWNTGAYLTCGDLWVCLSYDEARQYVPPQESDYTHYAFTVAEEDFEPFSQRLEQAGVTVWKQN
KSEGASFYFLDPDGHKLELHVGSLAARLAACREKPYAGMVFTSDEA
>GCA_000692315
KAAFRRLSENGENYSVPPLRRQPPAVYGEHGVVYVHLFTVPCPARKTKGHVMLQSLNHLTLAVSDLQKSVTFW
HELLGLALHARWNTGAYLTCGDLWVCLSYDEARQYVPPQESDYTHYAFTVAEEDFEPFSQRLEQAGVTVWKQN
KSEGASFYFLDPDGHKLELQVGSLAARLAACREKPYAGMVFTSDEA
>GCA_900077005
KAAFRRLSENGENYSVPPLRRQPPAVYGEDGVVYVHLFTVSRPARKTKGHVMLQSLNHLTLAVSNLQKSVTFW
HELLGLALHARWNTGAYLTCGDLWVCLSYDEARQYVPPQESDYTHYAFTVAEADFEPFSQKLEQAGVTVWKQN
KSEGASFYFLDPDGHKLELHVGSLAARLAACREKPYAGMVFTSDEA
>GCA_900075195
KAAFRRLSENGKNYSAPPLRRQPPAVYGEHGVVYVHLFTVSRPARKTKGHVMLQSLNHLTLAVSDLQKSVTFW
HELLGLALHARWNTGAYLTCGDLWVCLSYDEARQYVPPQESDYTHYAFTVAEEDFEPFSHRLEQAGVTVWKQN
KSEGASFYFLDPDGHKLELHVGSLAARLAACREKPYAGMVFTSDGA
>GCA_900076275
KAAFRRLSENGEYYSVPPLRRQPPAVYGEDGVVYVHLFTVPRPARKTKGHVMLQSLNHLTLAVSDLQKSVTFW
HELLGLALHARWNTGAYLTCGDLWVCLSYDEARQYVPPQESDYTHYAFTVSEADFEPFSNKLEQAGITVWKQN
KSEGASFYFLDPDGHKLELHVGSLAARLAACREKTYAGMVFTSDEA
>GCA_000958205
KAAFRRLSENGENYSVPPLRRQPPAVYGEHGVVYVHAFTVSRPARKTKGHVMLQSLNHLTLAVSDLQKSVTFW
HELLGLALHARWNTGAYLTCGDLWVCLSYDEARRNVPPQESDYTHYAFTVAEEDFEPFSQRLEQAGVTVWKQN
KSEGASFYFLDPDGHKLELHVGSLAARLAACREKPYAGMVFTSDEA
>GCA_001472895
```

KAAFRRLSENGENYSVPPLRRQPPAVYGEHGVVYVHAFTVSRPAQKTKGHVMLQSLNHLTLAVSDLQKSVTFW
HELLGLALHARWNTGAYLTCGDLWVCLSYDEARQYVPPQESDYTHYAFTVAEEDFEPFSHRLEQAGVTVWKQN
KSEGASFYFLDPDGHKLELHVGSLAARLAACREKPYAGMVFTSDGA
>GCA_000966095
KAAFRRLSENGKNYSVPPLRRQPPAVYGEHGVVYVHLFTVPRPARKTKGHVMLQSLNHLTLAVSDLQKSVTFW
HELLGLALHARWNTGAYLTCGDLWVCLSYDEARRNVPPQESDYTHYAFTVAEDDFEPFSHRLEQAGVTVWKQN
KSEGASFYFLDPDGHKLELHVGSLAARLAACREKPYAGMVFTSDGA
>GCA_001190105
KAAFRRLSENGENNSEPPLRRQPPAVYGEHGVVYGHLFTVSRPARKTKGHVMLQSLNHLTLAVSDLQKSITFW
HELLGLTLHARWNTGAYLTCGDLWVCLSYDEARQYVPPQESDYTHYAFTVAEADFEPFSHRLEQAGVTVWKQN
KSEGASFYFLDPDGHKLELHVGSLAARLAACREKPYAGMVFTSDEA
>GCA_000492715
KAAFRRLSENGENYSVPPLRRQPPAVYGEDGVVYVHAFTVSRPARKTKGHVMLQSLNHLTLAVSDLQKSVTFW
HELLGLALHARWNTGAYLTCGDLWVCLSYDEARQYVPPQESDYTHYAFTVTEEDFEPFSHRLEQAGVTVWKQN
KSEGASFYFLDPDGHKLELHVGCLAARLAACREKPYAGMVFTSDEA
>GCA_000512375
KAAFRRLSENGENYSMPPLRRQPPAVYGEHGVVYVHEVTVPRLAQKTKGHVMLQSLNHLTLAVSDLQKSVTFW
HELLGLTLHARWNTGAYLTCGDLWVCLSYDEARGYVPPQESDYTHYAFTVAAEDFEPFSHKLEQAGVTVWKQN
KSEGASFYFLDPDGHKLELHVGSLAARLAACREKPYAGMVFTSDEA
>GCA_001487035
KAAFRRLSENGENYSVPPLRRQPPAVYGEHGVVYVHLFTVPRPARKTKGLVMLQSLNHLTLAVSDLQKSVTFW
HELLGLPLHARWNTGAYLTCGDLWVCLSFDEARQYVPPQESDYTHYAFTVTEEDFEPFSHRLEQAGVTVWKQN
KSEGASFYFLDPDGHKLELHVGSLAARLAACREKPYSGMVFTSDEA
>GCA_001631195
KAAFRRLSENGENYSVPPLRRQPPAVYGEHGVVYVHFFTVSRPARKSKGHVMLQSLNHLTLAVSDLQKSVTFW
HELLGLALHARWNTGAYLTCGDLWVCLSYDEARRVVPPQESDYTHYAFTVSEADFEPFSQRLEQAGVTVWKQN
KSEGASFYFLDPDGHKLELHVGSLAARLAACREKPYAGMVFTSDEA
>GCA_000958805
KAAFRRLSENGEYYSVPPLRRQPPAVYGEDGVVYVHLFTVPRPARKTKGHVMLQSLNHLTLAVSDLQKSVTFW
HELLGLALHARWNTGAYLTCGDLWVCLSYDEARQYVPPQESDYTHYAFTVSEADFEPFSNKLEQAGITVWKQN
KSEGASFYFLDPDGHKLELHVGSLAARLAACREKPYAGMVFTSDEA
>GCA_000315775
KAAFRRLSENGKNYSVPPLRRQPPAVYGEDGVVYVHLFTVPRPARKTKGHVMLQSLNHLTLAVSDLQKSVTFW
HELLGLTLHARWNTGAYLTCGDLWVCLSYDEARRYVPPQESDYTHYAFTVAEEDFEPFSQRLKQAGVTVWKQN
KSEGASFYFLDPDGHKLELHVGSLAARLAACREKPYAGMLFTSDEA
>GCA_001055365
KAAFRRLSENGENYSVPPLRRQPPAVYGEHGVVYVHAFTVSRPARKTKGHVMLQSLNHLTLAVSDLQKSVTFW
HELLGLALHARWNTGAYLTCGDLWVCLSYDEARQYVPPQESDYTHYAFTVAEEDFEPFSHRLEQAGVTVWKQN
KSEGASFYFLDPDGHKLELHVGSLAARLAACREKPYAGMVFTSDGA
>GCA_000784905
KAAFRRLSENGKNYSAPPLRRQPPAVYGEHGVVYVHAFTVSRPARKIKGHVMLQSLNHLTLAVSDLQKSVTFW
HELLGLTLHARWNTGAYLTCGDLWVCLSYDEARQYVPPQESDYTHYAFTVAEEDFEPLSQRLEQAGVTIWKQN
KSEGASFYFLDPDGHKLELHVGSLAARLAACREKPYAGMVFTSDEA
>GCA_001022695
KAAFRRLSENGENYSVPPLRRQPPAVYGEHGVVYVHAFTVSRPARKTKGHVMLQSLNHLTLAVSDLQKSVTFW
HELLGLALHARWNTGAYLTCGDLWVCLSYDEARQYVPPQESDYTHYAFTVAEEDFEPFSHRLEQAGIIVWKQN
KSEGASFYFLDPDGHKLELHVGSLAARLAACREKPYAGMVFTSDEA
>GCA_000952605
KAAFRRLSENGENYSVPPLRRQPPAVYGEHGVVYVHAFTVSRPARKTKGHVMLQSLNHLTLAVSDLQKSVTFW
HELLGLTLHARWNTGAYLTCGDLWVCLSYDEAREYVPPQESDYTHYAFTVAEADFEPFSQRLEQAGVTVWKQN
KSEGASFYFLDPDGHKLELHVGSLAARLAACREKPYAGMVFTSDEA
>GCA_000530005

VKAAFRRLSENGENYSVPPLRRQPPAVYGEDGVVYVHAFTVPRPARKTKGHVMLQSLNHLTIAVSDLQKSVTF
WHALLGLTLHARWNTGAYLTCGELWVCLSYDEARRYVPPQESDYTHYAFTVAEEDFEPFSQRLEQAGVTVWKQ
NKSEGASFYFLDPDGHKLELHVGSLAARLAACREKPYA
>GCA_001006645
ENYSVPPLRRQPPAVYGEHGVVYVHLFTVSRPARKTKGHVMLQSLNHLTLAVSDLQKSVTFWHELLGLALHAR
WNTGAYLTCGDLWVCLSYDEARRVVPPQESDYTHYAFTVSEADFEPFSQRLEQAGVTVWKQNKSEGASFYLLD
PDGHKLELHVGSLAARLAACREKPYAGMVFTSDEA
>GCA_000264705
NYSMPPLRRQPPAVYGEHGVVYVHLFTVPRPARKTKGHVMLQSLNHLTLAVSDLQKSVTFWHELLGLTLHARW
NTGAYLTCGELWVCLSYDEARQYVPPQESDYTHYAFTVAEEDFEPFSQRLKQTGVTVWKQNKSEGASFYFLDP
DGHKLELHVGSLAARLAACREKPYAGMVFTSDEA
>GCA_900075785
LRRQPPAVYGEHGVVYVHLITVSRPARKTKGHVMLQSLNHLTLAVSDLQKSVTFWHELLGLTLHARWNTGAYL
TCGDLWVCLSYDEARQYVPPQESDYTHYAFTVAEADFEAFSQRLEQAGVIVWKQNKSEGASFYFLDPDGHKLE
LHVGSLAARLAACREKPYAGMVFTSDGA
>GCA_900075885
LRRQPPAVYGEHGVVYVHLFTVPRPARKTKGHVMLQSLNHLTLAVSDLQKSVTFWHELLGLTLHARWNTGAYL
TCGELWVCLSYDEARQYVPPQESDYTHYAFTVAEEDFEPLSQRLEQAGVTVWKQNKSEGASFYFLDPDGHKLE
LHVGSLAARLAACREKPYAGMVFTSDGA
>GCA_900076925
LRRQPPAVYGEHGVVYVHLFTVPRPARKTKGHVMLQSLNHLTLAVSDLQKSVTFWHELLGLALHARWNTGAYL
TCGDLWVCLSYDEARQYVPPQESDYTHYAFTVAEEDFEPLSQRLEQAGVTVWKQNKSEGASFYFLDPDGHKLE
LHVGSLAARLAACREKPYAGMVFTSDEA
>GCA_900077575
LRRQPPAVYGEHGVVYVHLITVSRPARKTKGHVMLQSLNHLTLAVSDLQKSVTFWHELLGLALHARWNTGAYL
TCGDLWVCLSYDEARQYVPPQESDYTHYAFTVAEADFEPLSQRLEQAGVTVWKQNKSEGASFYFLDPDGHKLE
LHVGSLAARLAACREKPYAGMVFTSDGA
>GCA_001052795
IMRTYKNKEELKKEINRTYTKYILEFETIPEELKDKRVEEVDRTPAENLAYQVGWTNLILKWENDERNGLSVK
TPSDQFKWNQLGELYKWFTNTYAHLPLKELEEILNRNIDDINMMIDSMSDEDLFTAHKRKWADEATKTAVWEV
YKFIHVNTVAPFGTFRTKIRKWKRLAL
>GCA_900143315
MRTYESKEALIEAIQIASQKYLAEFAEIPETLKDHRIETVAKTPSENLAYQLGWLNLLLSWEEQEQRGLTVQT
PAEGYKWNQLGALYQSFYQAYGQMSLESQLIALQDTLEKLLHWIDSLSEDELFLPQQRAWATTKAQWPLWKWI
HINSVAPFTSFRTQIRKWKKACL
>GCA_000686165
VKAAFRRLSENGKNYSVPPLGRQPPAVYGEHGVVYVHLFTVPRPARKTKGHVMLQSLNHLTLAVSDLQKSVSF
WHELLGLALHARWNTGAYLTCGDLWVCLSYDEARQYVPPQESDYTHYAFTVAEEDYEVFSQRLEQAGVTVWKQ
NKSEGRRSIFSTRTG
>GCA_000948705
YVHEVTVPRLAQKTKGHVMLQSLNHLTLAVSDLQKSVTFWHELLGLTLHARWNTGAYLTCGDLWVCLSYDEAR
GYVPPQESDYTHYAFTVAAEDFEPFSHKLEQAGVTVWKQNKSEGASFYFLDPDGHKLELHVGSLAARLAACRE
KPYAGMVFTSDEA
>GCA_900076285
LYRITSGAKNKRHVMLQSLNHLTLAVSDLQKSVTFWHELLGLTLHARWNTGAYLTCGDLWVCLSYDEARQYVP
PQESDYTHYAFTVTEEDFEPFSQRLEQAGVTVWKQNKSEGASFYFLDPDGHKLELHVGSLAARLAACREKPYA
GMVFTSDEA
>GCA_000389255
MLQSLNHLTLAVSNLQTSLTFWRDLLGLQLHAEWDTGASLPCGALWVCLSYDVSHLTCGDLWVCLSYDVSRNY
VAPQESDYTHYAFSIAPEDFEPFSCKLKQAGVTVWKDNKSEGQSFYFLDPDGHKLELHVGDLASRLTQCREKP
YSGMRF
>GCA_001078175

```
MLSGLNHLTLAVSQLAPSVAFYQQLPGMTLHARWDNGAYFSCGDLWLCLSLDPQRRVTPPEESDYTHYAFSIA
EEDFALFVGRLNAAGVPVWKTNKSEGASHYFLDPDGHKLELHVGNLAQRLAACRAKPYKGMVFFGEDEQAAGN
P
>GCA_001030775
MLSGLNHLTLAVSQLAPSVAFYQQLPGMTLRARWDNGAYFSCGDLWLCLSLDPQRRVTPPEESDYTHYAFSIA
EADFALFAGRLNAAGVPVWKTNKSEGASHYFLDPDGHKLELHVGNLAQRLAACRAKPYKGMVFFGEDEQAAGN
P
>GCA_900083885
MLSGLNHLTLAVSQLAPSVAFYQQLPGMTLRARWDNGAYFSCGDLWLCLSLDPQRRVISPEESDYTHYAFSIA
EADFALFAGRLNAAGVPVWKTNKSEGASHYFLDPDGHKLELHVGNLAQRLAACRAKPYKGMVFFGEDEQAAWN
P
>GCA_900083605
MLSGLNHLTLAVSQLAPSVAFYQQLPGMTLHARWDNGAYFSCGDLWLCLSLDPQRRVTPPEESDYTHYAFSIA
EEDFALFAGRLNAAGVPVWKTNKSEGASHYFLDPDGHKLELHVGNLARRLAACREKPYKGMVFFGEDEQTVGN
P
>GCA_900083695
MLSGLNHLTLAVSQLAPSVAFYQQLPGMTLRARWDNGAYFSCGDLWLCLSLDPQRRVTPPEESDYTHYAFSIA
EEDFAVFAGRLNAAGVPVWKTNKSEGASHYFLDPDGHKLELHVGSLAQRLAACRAKPYKGMVFFGEDEQTAGN
P
>GCA_000507385
MLSGLNHLTLAVSQLAPSVAFYQQLPGMTLRARWDNGAYFSCGDLWLCLSLDPQRRVISPEESDYTHYAFSIA
EADFALFAGRLNAAGVPVWKTNKSEGASHYFLDPDGHKLELHVGNLAQRLAACRKQPYKGMVFFGEDEQAAGN
P
>GCA_900075735
KAKGHVMLQSLNHLTLAVSDLQKSVTFWHELLGLTLHARWNTGAYLTCGDLWVCLSYDEARQYVPPQESDYTH
YAFTVAEADFAPFSHRLEQAGVTVWKQNKSAGASFYFLDPDGHKLELHVGSLAARLAACREKPYAGMVFTSDE
A
>GCA_900083635
MLSGLNHLTLAVSQLAPSVAFYQQLPGMTLRARWDKGAYFSCGDLWLCLSLDPQRRVISPEESDYTHYAFSIA
EADFALFAGRLNAAGVPVWKTNKSEGASHYFLDPDGHKLELHVGNLAQRLAACRKQPYKGMVFFGEDEQAAGN
P
>GCA_001548355
MLSGLNHLTLAVSQLAPSVAFYQQLPGMTLHARWDNGAYFSCGDLWLCLSLDPQRRVTPPEESDYTHYAFSIA
EEDFALFAGRLNAAGVPVWKTNKSEGASHYFLDPDGHKLELHVGNLARRLAACREKPYKGMVFFGEDEQTAGN
P
>GCA_900083565
MLSGLNHLTLAVSQLAPSVAFYQQLPGMTLRARWDNGAYFSCGDLWLCLSLDPQRRVISPEESDYTHYAFSIA
EADFALVVGRLNAAGVPVWKTNKSEGASHYFLDPDGHKLELHVGNLAQRLAACRAKPYKGMVFFGEDEQAAGN
P
>GCA_900083575
MLSGLNHLTLAVSQLAPSVAFYQQLPGMTLRARWDNGAYFSCGDLWLCLSLDPQRRVISPEESDYTHYAFSIA
EADFALFAGRLNAAGVPVWKTNKSEGASHYFLDPDGHKLELHVGNLAQRLAACRAKPYKGMVFFGEDEQAAGN
P
>GCA_900083625
MLSGLNHLTLAVSQLAPSVAFYQQLPGMTLRARWDNGAYFSCGDLWLCLSLDPQRRVTPPEESDYTHYAFSIA
EEDFALFVGRLNAAGVPVWKTNKSEGASHYFLDPDGHKLELHVGNLAQRLAACRAKPYKGMVFFGKDEQAAGN
P
>GCA_900083825
MLSGLNHLTLAVSQLAPSVAFYQQLPGMTLRARWDNGAYFSCGDLWLCLSLDPQRRVISPEESDYTHYAFSIA
EADFALVVGRLNAAGVPVWKTNKSEGASHYFLDPDGHKLELHVGNLAQRLAACRKQPYKGMVFFGEDEQAAGN
P
>GCA_001065715
```

MLSGLNHLTLAVSQLAPSVAFYQQLPGMTLRARWDKGAYFSCGDLWLCLSLDPQRRVTPPEESDYTHYAFSIA
EADFALFAGRLNAAGVPVWKTNKSEGASHYFLDPDGHKLELHVGNLAQRLAACRAKPYKGMVFFGEDEQTAGN
P
>GCA_001594375
MLSGLNHLTLAVSQLAPSVAFYQQLPGMTLRARWDNGAYFSCGDLWLCLSLDPQRRVTPPEESDYTHYAFSIA
EADFALFAGRLNAAGVPVWKTNKSEGASHYFLDPDGHKLELHVGNLAQRLAACRAKPYKGMVFFGEDEQAAGD
P
>GCA_000247855
MLSGLNHLTLAVSQLAPSVAFYQQLPGMTLRARWDKGAYFSCGDLWLCLSLDPQRRVTPPEESDYTHYAFSIA
EADFALFAGRLNAAGVPVWKTNKSEGASHYFLDPDGHKLELHVGNLAQRLAACRAKPYKGMVFFGEDEQAAGN
P
>GCA_900084875
MLSGLNHLTLAVSQLAPSVAFYQQLLGMTLHARWDSGAYLSCGDLWLCLSLDPQRRVTPPEESDYTHYAFSIS
EADFARFAARLEVVGVAVWKLNRSEGASHYFLDPDGHKLELHVGSLAQRLAACREQPYKGMVFFDDEVITGN
>GCA_001631845
MLSGLNHLTLAVSQLAPSVAFYHQLLGMTLHARWDSGAYLSCGDLWLCLSLDPQRRVTPPEESDYTHYAFSIS
EADFASFAARLEAAGVAVWKLNRSEGESHYFLDPDGHKLELHVGSLAQRLAACREQPYKGMVFFEDEVVTGN
>GCA_000281755
MLSGLNHLTLAVSQLAPSVAFYQQLLGMTLHARWDSGAYLSCGDLWLCLSLDPQRRVTPPEESDYTHYAFSIS
EADFASFAARLEAAGVAIWKLNRSEGASHYFLDPDGHKLELHVGSLAQRLAACREQPYKGMVFFEDEVVTGN
>GCA_001052235
MLSGLNHLTLAVSQLAPSVAFYQQLPGMTLHARWDNGAYFSCGDLWLCLSLDPQRRVTPPEESDYTHYAFSIA
EEDFALFAGRLNAAGVPVWKTNKSEGASHYFLDPDGHKLELHVGNLARRLAACREKPYKGMVFLARTNRRPGT
>GCA_000492795
MLSGLNHLTLAVSQLAPSVAFYQQLLGMTLHARWDSGAYLSCGDLWLCLSLDPQRRVTPPEESDYTHYAFSIS
EADFARFAARLEVAGVAVWKLNRSEGASHYFLDPDGHKLELHVGSLAQRLAACREQPYKGMVFFDDEVVTGN
>GCA_000567605
MLSGLNHLTLAVSQLAPSVAFYQQLLGMTLHARWDSGAYLSCGDLWLCLSLDPQRRITPPEESDYTHYAFSIS
EADFASFAARLEVAGVAVWKLNRSEGASHYFLDPDGHKLELHVGSLAQRLAACREQPYKGMVFFEDEVVTGN
>GCA_000693035
MLSGLNHLTLAVSQLAPSVAFYQQQLGMTLHARWDSGAYLSCGDLWLCLSLDPQRRVTPPEESDYTHYAFSIS
EADFARFAARLEVAGVAVWKLNRSEGASHYFLDPDGHKLELHVGSLAQRLAACREQPYKGMVFFDDEVVTGN
>GCA_000493135
MLSGLNHLTLAVSQLAPSVAFYQQLLGMTLHARWDSGAYLSCGDLWLCLSLDPQRRVTPPEESDYTHYAFSIS
EADFASFAARLEAAGVAIWKLNRSEGASHYFLDPDGHKLELHVGSLAQRLAACREQPYKGMVFFAEQGALG
>GCA_000822465
MLSGLNHLTLAVSQLAPSVAFYQQQLGMTLHARWDSGAYLSCGDLWLCLSLDPQRRVTPPEESDYTHYAFSIS
EADFASFAARLEAAGVAVWKLNRSEGASHYFLDPDGHKLELHVGSLAQRLAACREQPYKGMVFFAEQGALG
>GCA_001457255
MLSGLNHLTLAVSQLAQSVAFYQQLLGMTLHARWDSGAYLSCGDLWLCLSLDPQRRVTPPEESDYTHYAFSIS
EADFASFAARLEAAGVAVWKLNRSEGASHYFLDPDGHKLELHVGSLAQRLAACREQPYKGMVFFAEQGALG
>GCA_000821705
MLSGLNHLTLAVSQLAPSVAFYQQLLGMTLHARWDSGAYLSCGDLWLCLSLDPQRRVTPPEESDYTHYAFSIS
EADFASFAARLEAAGVAIWKLNRSEGASHYFLDPDGHKLELHVGSLAQRLAACREQPYKGMVFFDEQGALG
>GCA_000565135
MLSGLNHLTLAVSQLAPSVAFYQQLLGMTLHARWDSGAYLSCGDLWLCLSLDPQRRVTPPEESDYTHYAFSIS
EADFASFAARLEVAGVAVWKLNRSEGASHYFLDPDGHKLELHVGSLAQRLAACREQPYKGMVFFDEQGALG
>GCA_000950145
MLSGLNHLTLAVSQLAPSVAFIISYSARCTRAGIAARIFPAAICGCACRWIPQRRVTPPEESDYTHYAFSISE
ADFASFAARLEVAGVAVWKLNRSEGESHYFLDPDGHKLELHVGNLAQRLAACREQPYKGMVFFGE
>GCA_000492435
GHVMLQSLNHLTLAVSDLQKSVTFWHELLGLTLHARWNTGAYLTCGDLWVCLSYDEARRYVPPQESDYTHYAF
TVTEEDFEPFSQRLEQAGVTVWKQNKSEGASFYFLDPDGHKLELHVGSLAARLAACREKPYAGMVFTSDGA
>GCA_900076565

GHVMLQSLNHLTLAVSDLQKSVTFWHELLGLTLHARWNTGAYLTCGDLWVCLSYDEARRYVPPQESDYTHYAF
TVAEADFEPFSQRLEQAGVTVWKQNKSEGASFYFLDPDGHKLELHVGSLAARLAACREKPYAGMVFTSDKA
>GCA_001270135
GHVMLQSLNHLTLAVSDLQKSVTFWHELLGLTLHARWNTGAYLTCGDLWVCLSYDEARRNVPPQESDYTHYAF
TAAEEDFEPFSQRLKQAGVTVWKQNKSEGASFYFLDPDGHKLELHVGSLAARLAACREKPYAGMVFTSNEA
>GCA_900078165
GHVMLQSLNHLTLAVSDLQKSVTFWHELLGLTLHARWNTGAYLTCGDLWICLSYDEARRYVPPQESDYTHYAF
TVAEADFEPFSQMLEQAGVTVWKQNKSEGASFYFLDPDGHKLELHVGSLAARLAACREKPYAGMVFTSDEA
>GCA_900075235
GHVMLQSLNHLTLAVSDLQKSVTFWHELLGLALHARWNTGAYLTCGDLWVCLSYDEAHRYVPPQESDYTHYAF
TVAEEDFEPFSQRLEQAGVTVWKQNKSEGASFYFLDPDGHKLELHVGSLAARLAACREKPYAGMVFTSDEA
>GCA_001631365
GHVMLQSLNHLTLAVSDLQKSVTFWHELLGLALHARWNTGAYLTCGDLWVCLSYDEARQYVPPQESDYTHYAF
TVAEEDFEPFSQRLEQAGVTVWKQNKSEGASFYFLDPDGHKLELHMGSLAARLAACREKPYAGMVFTSDEA
>GCA_900077405
GHVMLQSLNHLTLAVSDLQKSVTFWHELLGLTLHARWNTGAYLTCGDLWVCLSYDEARQYVPPQESDYTHYAF
TVAEEDFEPFSQRLEQAGVTVWKQNKSEGASFYFLDPDGHKLELHVGSLAARLAACREKPYAGMVFTSDEA
>GCA_001055165
GHVMLQSLNHLTLAVSDLQKSVTFWHELLGLTLHARWNTGAYLTCGDLWVCLSYDEARQYVPPQESDYTHYAF
TVAEAEFEPFSNKLEQAGVIVWKQNKSEGASFYFLDPDGHKLELHVGSLAARLAACREKPYAGMVFTSDEA
>GCA_000492675
GHVMLQSLNHLTLAVSDLQKSVTFWHELLGLALHAHWNTGAYLTCGDLWVCLSYDEARQFVPPQESDYTHYAF
TVAEADFEPFSQRLEQAGVTVWKQNKSEGASFYFLDPDGHKLELHVGSLAARLAACREKPYAGMVFTSDEA
>GCA_000783855
GHVMLQSLNHLTLAVSDLQKSVTFWHELLGLALHARWNTGAYLTCGDLWVCLSYDEARRYVPPQESDYTHYAF
TVAEADFEPFSQRLEQAGVTVWKQNKSEGASFYFLDPDGHKLELHVGSLAARLAACREKPYAGMVFTSDEA
>GCA_900075915
GHVMLQSLNHLTLAVSDLQKSVTFWHELLGLTLHARWNTGAYLTCGDLWVCLSYDEARQYVPPQESDYTHYAF
TVAEADFEPFSQRLEQADVTVWKQNKSEGASFYFLDPDGHKLELHVGSLAARLAACREKPYAGMVFTSDKA
>GCA_900077895
GHVMLQSLNHLTLAVSDLQKSVTFWHELLGLALHARWNTGAYLTCGDLWVCLSYDEARQYVPPQESDYTHYAF
TVAEEDFEPFSQRLEQAGVTVWKQNKSEGASFYFLDPDGHKLELHVGSLAARLAACHEKPYAGMVFTSDEA
>GCA_900076035
GHVMLQSLNHLTLAVSDLQKSVTFWHELLGLALHARWNTGAYLTCGDLWVCLSYDEARRYVPPQESDYTHYAF
TVAEADFEPFSQRLEQAGVTVWKQNKSEGASFYFLDPDGHKLELHVGSLAARLAACREKPYAGMVFTSDGA
>GCA_000492575
GHVMLQSLNHLTLAVSDLQKSVTFWHELLGLALHARWNTGAYLTCGDLWVCLSYDEARQYVPPQESDYTHYAF
TVAEEDFEPFSQRLEQAGVTVWKQNKSEGASFYFLDPDGHKLELHVGSLAARLAACREKPYAGMVFTSDEA
>GCA_000770295
MLTGMNHLTLAVTDLDRSLHFYRDILKMTLHTRWKYGAYLTCGELWICLSADPEIIHRPIHQGYTHYAFTLPP
EQFPAFRSLLAAHQITLWKRNRSEGDSVYFLDPDGHQLEAHSGGIQQRLDACREAPYEEMIFPAPGQINV
>GCA_000966695
MLTGMNHLTLAVADLDRSLHFYRDILKMTLHTRWKYGAYLTCGELWICLSADPEIIHRPIHQGYTHYAFTLPP
EQFPAFRSLLAAHQITLWKRNRSEGDSIYFLDPDGHQLEAHSGGIQQRLDACREAPYEEMIFPAPGQINV
>GCA_900142745
MLTGMNHLTLAVADLDRSLHFYHDILKMTLHTRWKYGAYLTCGELWICLSADPEIIHRPIHQGYTHYAFTLPP
EQFPAFRSLLAAHQITLWKRNRSEGDSVYFLDPDGHQLEAHSGGIQQRLDACREAPYEEMIFPAPGQINV
>GCA_001006565
MLTGINHLTLAVADLDRSLHFYHGILEMTLHARWKYGAYLTCGDLWICLSADPEIIHRPVHQGYTHYAFTLPP
ERFPAFRALLASHQITLWKHNRSEGDSVYFLDPDGHQLEAHSGGIQQRLNACREAPYEEMIFPPTGQINV
>GCA_001462885
MLSGLNHLTLAVSQLAPSVAFYQQLLGMTLHARWDSGAYLSCGDLWLCLSLDPQRRITPPEESDYTHYAFSIS
EADFASFAARLEAAGVAIWKLNRSEGASHYFLDPDGHKLELHVGSLAQRLAACREQPYKGMVFFDEQGA
>GCA_001870615

MLSGLNHLTLAVSQLAPSVAFYQQLLGMTLHARWDSGAYLSCGDLWLCLSLDPQRRVTPPEESDYTHYAFSIS
EADFASFAARLEVAGVAVWKLNRSEGASHYFLDPDGHKLELHVAVLPSGWPPAANSRIRGWCFLISEAAS
>GCA_000694455
MLSGLNHLTLAVSQLAQSVAFYQQLLGMTLHARWDSGAYLSCGDLWLCLSLDPQRRVTPPEESDYTHYAFSIS
EADFASFAARLEAAGVAVWKLNRSEGASHYFLDPDGHKLELHVGSLAQRLAACREQPYKGMVFFDEQGA
>GCA_001274995
MLTGINHLTLAVADLDRSLHFYHGILEMTLHARWKYGAYLTCGELWICLSADPEIIHRPVHQGYTHYAFTLPP
ERFPAFRALLASHQITLWKHNRSEGDSVYFLDPDGHQLEAHSGGITQRLNACREAPYEEMIFPPTGQINV
>GCA_000223435
MLSGLNHLTLAVSQLAPSVAFYQQLLGMTLHARWDSGAYLSCGDLWLCLSLDPQRRVTPPEESDYTHYAFSIS
EADFASFAARLEAAGVAIWKLNRSEGASHYFLDPDGHKLELHVGSLAQRLAACREQPYKGMVFFDEQGA
>GCA_001879345
MLSGLNHLTLAVSQLAPSVAFYQQLLGMTLHARWDSGAYLSCGDLWLCLSLDPQRRVTPPEESDYTHYAFSIS
EADFASFAARLEAAGVAVWKLNRSEGASHYFLDPDGHKLELHVAVSPSGWPPAASSRIRGWCFLNSEAAT
>GCA_900083615
MLSGLNHLTLAVSQLAPSVAFYQQLPGMTLRARWDKGAYFSCGDLWLCLSLDPQRRVTPPEESDYTHYAFSIA
EADFALFAGRLNAAGVPVWKTNKSEGASHYFLDPDGHKLELHVGNLAQRLAACRAKPYKGMVFFGEDEQ
>GCA_000269585
MLSGLNHLTLAVSQLAPSVAFYQQLPGMTLRARWDNGAYFSCGDLWLCLSLDPQRRVTPPEESDYTHYAFSIA
EADFALFAGRLNAAGVPVWKTNKSEGASHYFLDPDGHKLELHVGNLAQRLAACRAKPYKGMVFFGEDEQ
>GCA_001879415
MLSGLNHLTLAVSQLAPSVAFYQQLLGMTLHARWDSGAYLSCGDLWLCLSLDPQRRVTPPEESDYTHYAFSIS
EADFASFAARLEAAGVAVWKLNRSEGASHYFLDPDGHKLELHVAVSPSGWPPAASSRIRGWCFLNSEAA
>GCA_001674985
MLSGLNHLTLAVSQLAPSVAFYQQLLGMTLHARWDSGAYLSCGDLWLCLSLDPQRRVTPPEESDYTHYAFSIS
EADFASFAARLEAAGVAIWKLNRSEGASHYFLDPDGHKLELHVAVSPSVWPPAANSRIRGWCFLISEAA
>GCA_001621665
MLNALNHLTLAVSNLPASITFWRDLLGLRLHAEWHTGAYLTCGDLWLCLSYDETRTFIPPQNSDYTHYAFSVE
PEHFDAVAQKLKDAGVTVWKENKSEGASFYFLDPDGHKLELHVGDLAARLAACREKPYAGMVFTSDEA
>GCA_000633515
MLTGMNHLTLAVADVDRSLHFYRDILEMTLHARWKYGAYLTCGELWICLSADPEIIHRPVHQGYTHYAFTLPP
EQFPAFRSLLAAHQITLWKRNRSEGNSVYFLDPDGHQLEVHSGGIQQRLDACREAPYEEMIFPDNTGE
>GCA_000950365
MLTGMNHLTLAVADVDRSLHFYRDILEMTLHARWKYGAYLTCGELWICLSADPEIIHRPVHQGYTHYAFTLPP
EQFPAFRSLLATHQITLWKRNRSEGNSVYFLDPDGHQLEVHSGGIQQRLDACREAPYEEMIFPDNTGE
>GCA_001879425
MLSGLNHLTLAVSQLAPSVAFYQQLLGMTLHARWDSGAYLSCGDLWLCLSLDPQRRVTPPEESDYTHYAFSIS
EADFASFAARLEAAGVAIWKLNRSEGASHYFLDPDGHKLELHVAVSPSVWPPAANSRIRGWCFLISE
>GCA_001006635
MLHALNHLTLAVSHLPTSIAFWRDLLGLRLHAEWDTGAYLTCGDLWLCLSYDDARQYVPPQNSDYTHYAFSVA
PEHFDTLVQILTAANVTVWKDNKSEGASFYFLDPDGHKLELHVGDLAARLAACREKPYAGMVFTADD
>GCA_900085185
MLSGLNHLTLAVSQLAPSVAFYQQLLGMTLHARWDSGAYLSCGDLWLCLSLDPQRRITPPEESDYTHYAFSIS
EADFASFAARLEVAGVAVWKLNRSEGASHYFLDSDGHKLELHVGSLAQRLAACREQPYKGMVFFDQ
>GCA_000692995
MLSGLNHLTLAVSQLAPSVAFYQQLLGMTLHARWDSGAYLSCGDLWLCLSLDPQRRITPSEESDYTHYAFSIS
EADFASFAARLEVAGVAVWKLNRSEGASHYFLDPDGHKLELHVGSLDQRLAACREQPYKGMVFFDQ
>GCA_000566845
MLSGLNHLTLAVSQLAPSVAFYQQLLGMMLHARWDSGAYLSCGDLWLCLLDPQRRVTPPEESDYTHYAFSISE
ADFASFAARLEAAGVAVWKLNRSEGASHYFLDPDGHKLELHVGSLAQRLAACREQPYKGMVFFDE
>GCA_001970155
MLSGLNHLTLAVSQLAPSVAFYQQLLGMTLHARWDSGAYLSCGDLWLCLSLDPQRRVTPPEESDYTHYAFSIS
EADFASFAARLEAAGVAIWKLNRSEGASHYFLDPDGHKLELHVGSLAQRLAACREQPYKGMVFFEE
>GCA_001033665

MLSGLNHLTLAVSQLAPSVAFYQQLLGMTLHARWESGAYLSCGDLWLCLSLDPQRRITPPEESDYTHYAFSIS
EADFACFAARLEVAGVAVWKLNRSEGASHYFLDPDGHKLELHVGSLAQRLAACREQPYKGMVFFDG
>GCA_001033805
MLSGLNHLTLAVSQLAPSVAFYQQLLGMTLHARWESGAYLSCGDLWLCLSLDPQRRITPPEESDYTHYAFSIS
EADFACFAARLEVAGVAVWKLNRSEGASHYFLDPDGHKLELHVGNLAQRLAACREQPYKGMVFFDQ
>GCA_001062535
MLSGLNHLTLAVSQLAPSVAFYQQQLGMTLHARWDSGAYLSCGDLWLCLSLDPQRRVTPPEESDYTHYAFSIS
EADFASFAARLEAAGVAVWKLNRSEGASHYFLGPGGHKLELHVGSLAQRLAACREQPYKGMVFFDQ
>GCA_001936035
MLSGLNHLTLAVSQLAPSVAFYQQLLGMTLHARWDSGAYLSCGDLWLCLSLDPQRRVTPPEESDYTHYAFSIS
EADFASFAARLEAAGVAVWKLNRSEGASHYFLDPDGHKLELHVGSLAQRLAACREQPYKGMVFFDE
>GCA_000805735
MLSGLNHLTLAVSQLAPSVAFYQQQLGMTLHARWDSGAYLSCGDLWLCLSLDPQRRVTPPEESDYTHYAFSIS
EADFASFAARLEAAGVAIWKLNRSEGASHYFLDPDGHKLELHVGSLAQRLAACREQPYKGMVFFDQ
>GCA_900093305
MLSGLNHLTLAVSQLAPSVAFYQQLLGMTLHARWESGAYLSCGDLWLCLSLDPQRRITPPEESDYTHYAFSIS
EADFACFTARLEVVGVAVWKLNRSEGASHYFLDPDGHKLELHVGSLAQRLAACREQPYRGMVFFDQ
>GCA_000383335
MLSGLNHLTLAVSQLAPSVAFYRRLLGMTLHARWDSGAYLSCGDLWLCLSLDPQRRVTPPEESDYTHYAFSIS
EADFASFAARLEVAGVAVWKLNRSEGESHYFLDPDGHKLELHVGSLAQRLAACREQPYKGMVFFEE
>GCA_900093015
MLSGLNHLTLAVSQLAPSVAFYQQQLGMTLHARWDSGAYLSCGDLWLCLSLDPQRRVTPPEESDYTHYAFSIS
EADFASFAARLEAAGAAIWKLNRSEGASHYFLDPDGHKLELHVGSLAQRLAACREQPYKGMVFFDQ
>GCA_001011295
MLSGLNHLTLAVSQLAPSVAFYHQLLGMTLHARWDSGAYLSCGDLWLCLSLDPQRRVTPPEESDYTHYAFSIS
EADFASFAARLEVAGVAVWKLNRSEGESHYFLDPDGHKLELHVGNLAQRLAACREQPYKGMAFFGE
>GCA_900085435
MLSGLNHLTLAVSQLAPSVAFYQQLLGMTLHARWESGAYLSCGDLWLCLSLDPQRRITPPEESDYTHYAFSIS
EADFACFAARLEVAGVAVWKLNRSEGASHYFLDPDGHKLELHVGSLAQRLAACREQPYRGMVFFDQ
>GCA_001011385
MLSGLNHLTLAVSQLAPSVAFYHQLLGMTLHARWDSGAYLSCGDLWLCLSLDPQRRVTPPEESDYTHYAFSIS
EADFASFAARLEVAGVALWKLNRSEGESHYFLDPDGHKLELHVGNLAQRLAACREQPYKGMVFFGE
>GCA_000754345
MLIGINHLTLAVSDIQKSISFYQTVLGMQLHASWERGAYLTCGALWVCLSYDPMRQEVAAAKRRDYTHYAFTV
SAEDFSDIVEKLHQAGVTVWKDNRSEGDSFYFLDPDGHQLEIHVGSLIDRLKSCRKKPYDGMVFYS
>GCA_900084795
MLSGLNHLTLAVSQLAPSVAFYQQLLGMTLHARWDSGAYLSCGDLWLCLSLDPQRRIIPPEESDYTHYAFSIS
EADFASFAARLEVAGVAVWKLNRSEGASHYFLDPDGHKLELHVGSLAQRLAACREQPYKGMVFFDQ
>GCA_000822705
MLSGLNHLTLAVSQLAPSVAFYQQQLGMTLHARWDSGAYLSCGDLWLCLSLDPQRRVTPPEESDYTHYAFSIS
EADFASFAARLEAAGVAVWKLNRSEGASHYFLDPDGHKLELHVGSLAQRLAACREQPYKGMVFFAE
>GCA_001914065
MLSGLNHLTLAVSQLAPSVAFYQQLLGMTLHARWDRGAYLSCGDLWLCLSLDPQRRVTPPEESDYTHYAFSIS
EADFASFAARLEAAGVAIWKLNRSEGASHYFLDPDGHKLELHVGSLAQRLAACREQPYKGMVFFDQ
>GCA_000220485
MLSGLNHLTLAVSQLAPSVAFYQQLLGMTLHARWDSGAYLSCGDLWLCLSLDPQRRVTPPEESDYTHYAFSIS
EADFASFAARLEAAGVAIWKLNRSEGASHYFLDPDGHKLELHVGSLAQRLAACREQPYKGMVFFEQ
>GCA_000334515
MLSGLNHLTLAVSQLAPSVAFYHQLLGMTLHARWDSGAYLSCGDLWLCLSLDPQRRVTPPEESDYTHYAFSIS
EADFTSFAARLEVAGVAVWKLNRSEGESHYFLDPDGHKLELHVGNLAQRLAACREQPYKGMAFFGE
>GCA_000957705
MLSGLNHLTLAVSQLAPSVAFYHQLLGMTLHARWDSGAYLSCGDLWLCLSLDPQRRVTPPEESDYTHYAFSIS
EADFAGFAARLEVAGVAVWKLNRSEGESHYFLDPDGHKLELHVGSLAKRLAACREQPYKGMVFFGE
>GCA_001631345

MLSGLNHLTLAVSQLAPSVAFYHQLLGMTLHARWDSGAYLSCGDLWLCLSLDPQRRVTPPEESDYTHYAFSIS
EADFASFAARLEVAGVAVWKLNRSEGESHYFLDPDGHKLELHVSNLAQRLAACREQPYKGMVFFEE
>GCA_000822225
MLSGLNHLTLAVSQLAPSVAFYQQLLGMTLHARWDCGAYLSCGDLWLCLSLDPQRRVTPPEESDYTHYAFSIS
EADFASFAARLEVAGVAVWKLNRSEGASHYFLDPDGHKLELHVGSLAQRLAACREQPYKGMVFFAE
>GCA_000936205
MLSGLNHLTLAVSQLAPSVAFYQQLLGMTLHARWDSGAYLSCGDLWLCLSLDPQRRITPPEESDYTHYAFSIS
EADFASFAARLEVAGVAVWKLNRSEGASHYFLDPDGHKLELHVGSLDLRLAACREQPYKGMVFFDQ
>GCA_001530015
MLSGLNHLTLAVSQLAPSVAFYQQLLGMTLHARWDSGAYLSCGDLWLCLSPDPQRRVTPPEESDYTHYAFSIS
EADFASFAARLEAAGVAIWKLNRSEGASHYFLDPDGHKLELHVGSLAQRLAACREQPYKGMVFFDQ
>GCA_001071835
MLSGLNHLTLAVSQLAPSVAFYHQLLGMTLHARWDSGAYLSCGDLWLCLSLDPQRRVTPPEESDYTHYAFSIS
EADFASFAARLEAAGVAVWKLNRSEGESHYFLDPDGHKLELHVGSLAQRLAACREQPYKGMVFFDE
>GCA_001463185
MLSGLNHLTLAVSQLAPSVAFYQQLLGMTLHARWDSGAYLSCGDLWLCLSLDPQRRITPPEESDYTHYAFSIS
EADFACFAARLEVAGVAVWKLNRSEGASHYFLDPDGHKLELHVGNLAQRLAACREQPYKGMVFFDQ
>GCA_000412575
MLSGLNHLTLAVSQLAPSVAFYQQLLGMTLHARWDSGAYLSCGDLWLCLSLDPQRRITPPEESDYTHYAFSIS
EADFASFAARLEAAGVAIWKLNRSEGASHYFLDPDGHKLELHVGSLAQRLAACREQPYKGMVFFDQ
>GCA_001011515
MLSGLNHLTLAVSQLAPSVAFYHQLLGMTLHARWDSGAYLSCGDLWLCLSLDPQRRVTPPEESDYTHYAFSIS
EADFASFAARLEVAGVAVWKLNRSEGESHYFLDPDGHKLELHVGSLAQRLAACREQPYKGMVFFDE
>GCA_000692155
MLSGLNHLTLAVSQLAPSVAFYHQLLGMTLHARWDSGAYLSCGDLWLCLSLDPQRRVTPPEESDYTHYAFSIS
EADFASFAARLEVAGVAVWKLNRSEGESHYFLDPDGHKLELHVGNLAQRLAACREQPYKGMVFFDE
>GCA_000827665
MLSGLNHLTLAVSQLAPSVAFYQQLLGMTLHARWDSGAYLSCGDLWLCLSLDPQRRVTPPEESDYTHYAFSIS
EADFARFAARLEVVGVAVWKLNRSEGASHYFLDPDGHKLELHVGSLAQRLAACREQPYKGMVFFDE
>GCA_001011595
MLSGLNHLTLAVSQLAPSVAFYHQLLGMTLHARWDSGAYLSCGDLWLCLSLDPQRRVTPPEESDYTHYAFSIS
EADFASFAARLEVAGVAVWKLNRSEGESHYFLDPDGHKLELHVGSLAKRLAACREQPYKGMVFFGE
>GCA_000492835
MLSGLNHLTLAVSQLAPSVAFYQQLLGMTLHARWDSGAYLSCGDLWLCLSLDPQRRITPPEESDYTHYAFTIS
EADFARFAARLEVAGVAVWKLNRSEGASHYFLDPDGHKLELHVGSLAQRLAACREQPYKGMVFFDE
>GCA_001066095
MLSGLNHLTLAVSQLAPSVAFYQQQLGMTLHARWDSGAYLSCGDLWLCLSLDPQRRITPPEESDYTHYAFSIS
EADFASFAARLEAAGVAIWKLNRSEGASHYFLDPDGHKLELHVGSLAQRLAACREQPYKGMVFFDE
>GCA_900085315
MLSGLNHLTLAVSQLAPSVAFYQQLLGMTLHARWDSGAYLSCGDLWLCLSLDPQRRVTPPEESDYTHYAFSIS
EADFASFAARLEAAGVAVWKLNRSEGASHYFLDPDGHKLELHVGSLAQRLAACREQPYKGMVFFAE
>GCA_001631815
MLSGLNHLTLAVSQLAPSVAFYHQLLGITLHARWDSGAYLSCGDLWLCLSLDPQRRVTPPEESDYTHYAFSIS
EADFASFTARLEVAGVAVWKLNRSEGESHYFLDPDGHKLELHVGSLAQRLAACREQPYKGMVFFGE
>GCA_001011795
MLSGLNHLTLAVGQLAPSVAFYHQLLGMTLHARWDSGAYLSCGDLWLCLSLDPQRRVTPPEESDYTHYAFSIS
EADFASFAARLESAGVAVWKLNRSEGESYYFLDPDGHKLELHVGSLAQRLAACREQPYKGMVFFDE
>GCA_001011355
MLSGLNHLTLAVSQLAPSAAFYHQLLGMTLHARWDSGAYLSCGDLWLCLSLDPQRRVTPPEESDYTHYAFSIS
EADFASFAARLEVAGVAVWKLNRSEGESHYFLDPDGHKLELHVGNLAQRLAACREQPYKGMVFFDE
>GCA_001034045
MLSGLNHLTLAVSQLAPSVAFYQQLLGMTLHARWDSGAYLSCGDLWLCLSLDPQRRITPPEESDYTHYAFSIS
EADFASFAARLEVAGVAVWKLNRSEGASHYFLDPDGHKLELHVGSLAQRLAACREQPYKGMVFFDE
>GCA_001597035

```
MLSGLNHLTLAVSQLAPSVAFYQQLLGMTLHARWDSGAYLSCGDLWLCLSLDPQRRVTPPEESDYTHYAFSIS
EADFARFAARLEAAGVAIWKLNRSEGASHYFLDPDGHKLELHVGSLAQRLAACREQPYKGMVFFAE
>GCA_900084815
MLSGLNHLTLAVSQLAPSVAFYQQLLGMTLHARWDSGAYLSCGDLWLCLSLDPQRRITPSEESDYTHYAFSIS
EADFASFAARLEVSGVAVWKLNRSEGASHYFLDPDGHKLELHVGSLAQRLAACREQPYKGMVFFDQ
>GCA_001720645
MLSGLNHLTLAVSQLAPSVAFYQQLLGMTLHARWDSGAYLSCGDLWLCLSLDPQRRVTPPEESDYPHYAFSIS
EADFASSAARLEAAGVAIWKLNRSEGASHYFLDPDGHKLELHVGSLAQRLAACREQPYKGMVFFDQ
>GCA_000019565
MLSGLNHLTLAVSQLAPSVAFYQQLLGMTLHARWDSGAYLSCGDLWLCLSLDPQRRITPPEESDYTHYAFSIS
EADFASFAARLEVAGVAVWKLNRSEGASHYFLDPDGHKLELHVGSLAQRLAACREQPYKGMVFFDQ
>GCA_001006555
MLSGLNHLTLAVSQLAPSVAFYHQLLGMTLHARWDSGAYLSCGDLWLCLSLDPQRRVTPPEESDYTHYAFSIS
EADFASFAARLEVAGVAVWKLNRSEGESHYFLDPDGHKLELHVGNLAQRLAACREQPYKGMVFFGD
>GCA_000723925
MLSGLNHLTLAVSQLAPSVAFYQQLLGMTLHARWDSGAYLSCGDLWLCLSLDPQRRVTPPEESNYTHYAFSIS
EADFASFAARLEAAGVAVWKLNRSEGASHYFLDPDGHKLELHVGSLAQRLAACREQPYKGMVFFDE
>GCA_001312905
MLSGLNHLTLAVSSLAPSVAFYLQLLGMTLHARWDGGAYLSCGDLWLCLSLDPQRRVTPPEESDYTHYAFSVA
EADFAGFSARLETAGVAVWKVNRSEGASYYFLDPDGHKLELHVGSLALRLAACREQPYKGMVFYDE
>GCA_900083685
MLSGLNHLTLAVSSLAPSVAFYHQLLGMTLHARWDGGAYLSCGDLWLCLSLDPQRRITPPEESDYTHYAFSVA
EADFAGFSARLETAGVAVWKVNRSEGASYYFLDPDGHKLELHVGSLALRLAACREQPYKGMVFYDE
>GCA_000534135
MLSGLNHLTLAVSQLAPSVAFYHQLLGMTLHARWDSGAYLSCGDLWLCLSLDPQRRVTPPEESDYTHYAFSIS
EADFASFAARLEVAGVAVWKLNRSEGESHYFLDPDGHKLELHVGNLPQRLAACREQPYKGMVFFGE
>GCA_000263095
MLSGLNHLTLAVSQLAPSVAFYQQLLGMTLHARWDSGAYLSCGDLWLCLSLDPQRRVTPPEESDYTHYAFSIS
EADFASFAARLEAAGVAIWKLNRSEGASHYFLDPDGHKLELHVGSLAQRLAACREQPYKGMVFFDE
>GCA_001011915
MLSGLNHLTLAVSQLAPSVAFYHQLLGMTLHARWDSGAYLSCGDLWLCLSLDPQRRVTPPEESDYTHYAFSIS
EADFASFAARLEVAGVAVWKLNRSEGESHYFLDPDGHKLELHVGNLAQRLAACREQPYKGMVFFEE
>GCA_000255975
MLSGLNHLTLAVSQLAPSVAFYQQLLGMTLHARWDSGAYLSCGDLWLCLSLDPQRRVTPPEESDYTHYAFSIS
EADFASFAARLEVAGVAVWKLNRSEGASHYFLDPDGHKLELHVGSLAQRLAACREQPYKGMVFFDQ
>GCA_000783515
MLSGLNHLTLAVSQLAPSVAFYQQLLGMTLHARWDSGAYLSCGDLWLCLSLDPQRRITPPEESDYTHYAFSIS
EADFASFAARLEAAGVAIWKLNRSEGASHYFLDPDGHKLELHVGSLAQRLAACREQPYKGMVFFEQ
>GCA_000492215
MLSGLNHLTLAVSQLAPSVAFYQQLLGMMLHARWDSGAYLSCGDLWLCLSLDPQRRVTPPEESDYTHYAFSIS
EADFASFAARLEAAGVAVWKLNRSEGASHYFLDPDGHKLELHVGSLAQRLAACREQPYKGMVFFDE
>GCA_001031245
MLSGLNHLTLAVSQLAPSVAFYQQLLGMTLHARWDSGAYLSCGDLWLCLSLDPQRRVTPPEESDYTHYAFSIS
EADFASFAARLEVAGVAVWKLNRSEGASHYFLDPDGHKLELHVGSLAQRLAACREQPYKGMVFFAE
>GCA_000492475
MLSGLNHLTLAVSQLAPSVAFYQQLLGMTLHARWDSGAYLSCGDLWLCLSLDPQRRITPSEESDYTHYAFSIS
EADFASFAARLEVAGVAVWKLNRSEGASHYFLDPDGHKLELHVGSLAQRLAACREQPYKGMVFFDE
>GCA_000474015
MLSGLNHLTLAVSQLAPSVAFYQQLLGMTLHARWDSGAYLSCGDLWLCLSLDPQRRVTPPEESDYTHYAFSIS
EADFASFAARLEAAGVAIWKLNRSEGASHYFHPDGHKLELHVGSLAQRLAACREQPYKGMVFFAE
>GCA_001888205
MLIGINHLTLAVSDIQKSISFYQTVLGMQLHASWERGAYLTCGALWVCLSYDPMRQEVAAAKCRDYTHYAFTV
SAEDFSDIVEKLHQAGVTVWKDNRSEGDSFYFLDPDGHQLEIHVGSLIDRLKSCRKKPYDGMVFYS
>GCA_001006575
```

MLSGLNHLTLAVSQLAPSVAFYQHLLGMTLHARWDSGAYLSCGDLWLCLSLDPQRRITPPEESDYTHYAFSIS
EADFASFAARLEVAGVAVWKLNRSEGASHYFLDPDGHKLELHVGSLDQRLAACREQPYKGMVFFDQ
>GCA_000409715
MLSGLNHLTLAVSQLAPSVAFYQQLLGMTLHARWDSGAYLSCGDLWLCLSLDPQRRVTPPEESDYTHYAFSIS
EADFASFAARLEAAGVAIWKLNRSEGASHYFLDPDGHKLELHVGSLAQRLAACREQPYKGMEFFDQ
>GCA_000755545
MLSGLNHLTLAVSQLAPSVAFYRRLLGMTLHARWDSGAYLSCGDLWLCLSLDPQRRVTPPEESDYTHYAFSIS
EADFASFAARLEVAGVAVWKLNRSEGESHYFLDPDGHKLELHVGNLAQRLAACREQPYKGMVFFEE
>GCA_000367165
MLSGLNHLTLAVSQLAPSVAFYQQLLGMTLHARWDSGAYLSCGDLWLCLSLDPQRRVTPPEESDYTHYAFSIS
EADFARFAARLEVAGVAVWKLNRSEGASHYFLDPDGHKLELHVGSLAQRLAACREQPYKGMVFFDQ
>GCA_000694735
MLSGLNHLTLAVSQLAPSVAFYQQLLGMTLHARWDSGAYLSCGDLWLCLSLDPQRRITPPEESDYTHYAFSIS
EADFASFAARLEVAGVAVWKLNRSEGASHYFLDPDGHKLELHVGSLDQRLAACREQPYKGMVFFDQ
>GCA_001666015
MLSGLNHLTLAVSQLAPSVAFYQQLLGMTLHARWDSGAYLSCGDLWLCLSLDPQRRVTPPEESDYTHYAFSIS
EADFASFAARLGAAGVAVWKLNRSEGASHYFLDPDGHKLELHVGSLAQRLAACREQPYKGMVFFEQ
>GCA_900084115
MLSGLNHLTLAVSQLAPSVAFYQQLLGMTLHARWDSGAYLSCGDLWLCLSLDPQRRVTPPEESDYIHYAFSIS
EADFASFAARLEAAGVAIWKLNRSEGASHYFLDPDGHKLELHVGSLAQRLAACREQPYKGMVFFDE
>GCA_001033975
MLSGLNHLTLAVSQLAPSVAFYQQLLGMTLHARWDSGTYLSCGDLWLCLSLDPQRRVTPPEESDYTHYAFSIS
EADFASFAARLEAAGVAIWKLNRSEGASHYFLDPDGHKLELHVGSLAQRLAACREQPYKGMVFFDQ
>GCA_000821545
MLSGLNHLTLAVSQLAPGVAFYQQLLGMTLHARWDSGAYLSCGDLWLCLSLDPQRRVTPPEESDYTHYAFSIS
EADFASFAARLEVAGVAVWKLNRSEGASHYFLDPDGHKLELHVGSLAQRLAACREQPYKGMVFFDQ
>GCA_000956775
MLSGLNHLTLAVSQLAPSVAFYHQLLGMTLHARWDSGAYLSCGDLWLCLSLDPQRRVTPPEESDYTHYAFSIS
EADFAGFAARLEVAGVAVWKLNRSEGESHYFLDPDGHKLELHVGSLAQRLAACREQPYKGMVFFGE
>GCA_001280925
MLSGLNHLTLAVSQLAPSVAFYQQLLGMTLHARWDSGAYLSCGDLWLCLSLDPQRRVTPPEESDYTHYAFSIS
EADFARFAARLEVAGVAVWKLNRSEGASHYFLDPDGHKLELHVGSLVQRLAACREQPYKGMVFFDQ
>GCA_000219945
MLSGLNHLTLAVSQLAPSVAFYQQLLGMTLHARWDSGAYLSCGDLWLCLSLDPQRRVTPPEESDYTHYAFSIS
EADFASFAARLEAAGVAVWKLNRSEGASHYFLDPDGHKLELHVGSLAQRLAACREQPYKGMVFFDQ
>GCA_000498775
MLSGLNHLTLAVSQLAPSVAFYQQQLGMTLHARWDSGAYLSCGDLWLCLSLDPQRRVTPPEESDYTHYAFSIS
EADFASFAARLEAAGVAIWKLNRSEGASHYFLDPDGHKLELHVGSLAQRLAACREQPYKGMEFFDQ
>GCA_001030055
MLSGLNHLTLAVSQLAPSVAFYHQLIGMTLHARWDSGAYLSCGDLWLCLSLDPQRRVTPPEESDYTHYAFSIS
EADFASFAARLEVAGVAVWKLNRSEGESHYFLDPDGHKLELHVGNLAQRLAACREQPYKGMVFFGE
>GCA_000493115
MLSGLNHLTLAVSQLAPSVAFYQQLLGMTLHARWDSGAYLSCGDLWLCLSLDPQRRVTPPEESDYTHYAFSIS
EVDFASFAARLEVAGVAVWKLNRSEGASHYFLDPDGHKLELHVGSLAQRLAACREQPYKGMVFFDQ
>GCA_000693815
MLSGLNHLTLAVSQLAPSVAFYQQLLGMTLHARWDSGAYLSCGDLWLCLSLDTQRRVTPPEESDYTHYAFSIS
EADFASFAARLEAAGVAIWKLNRSEGASHYFLDPDGHKLELHVGSLAQRLAACREQPYKGMVFFEQ
>GCA_001974865
MLSGLNHLTLAVSQLAPSVAFYHQLLGMTLHARWDSGAYLSCGDLWLCLSLDPQRRVTPPEESDYTHYAFSIS
EADFAGFAARLEVAGVAVWKLNRSEGESHYFLDPDGHKLELHVGNLAQRLAACREQPYKGMVFFGE
>GCA_001373075
MLSGLNHLTLAVSQLAPSVAFYQQLLGMTLHARWDSGAYLSCGDLWLCLSLDPQRRVTPPEESDYTHYAFSIS
EADFASFAARLEVAGVAVWKLNRSEGASHYFLDPDGHKLELHVGSLAQRLAACREQPYKGMVFFDE
>GCA_000822485

MLSGLNHLTLAVSQLAPSVAFYQQQLGMTLHARWDSGAYLSCGDLWLCLSLDPQRRVTPPEESDYTHYAFSIS
EADFASFAARLEAAGVAVWKLNRSEGASHYFLDPDGHKLELHVGSLAQRLAACREQPYKGMVFFDQ
>GCA_001968995
MLIGINHLTLAVSDIQKSISFYQTVLGMQLHASWERGAYLTCGALWVCLSYDPMRQEVAAAKRRDYTHYAFTV
SAEDFSDIVEKLHQAGVTVWKDNCSEGDSFYFLDPDGHQLEIHVGLLIDRLKSCRKKPYDGMVFYS
>GCA_000714635
MLSGLNHLTLAVSQLAPSVAFYQQLLGMTLHARWDSGAYLSCGDLWLCLSLDPQRRITPSEESDYTHYAFSIS
EADFASFAARLEVAGVAVWKLNRSEGASHYFLDPDGHKLELHVGSLAQRLAACREQPYKGMVFFDQ
>GCA_001404095
MLSGLNHLTLAVSQLAPSVAFYQQLLGMTLHARWDSGAYLSCGDLWLCLSLDPQRRVTPPEESDYTHYAFSIS
EADFASFAARLEAAGVAIWKLNRSEGASHYFLDPDGHKLELHVGSLSQRLAACREQPYKGMVFFDE
>GCA_900084085
MLSGLNHLTLAVSQLAPSVAFYQQLLGMTLHARWDSGAYLSCGDLWLCLSLDPQRRITPPEESDYTHYAFSIS
EADFASFAARLEVAGVAVWKLNRSEGASHYFLDPDGHKLELHVGSLAQRLAACCEQPYKGMVFFAE
>GCA_900092875
MLSGLNHLTLAVSQLAPSVAFYQQLLGMMLHARWDSGAYLSCGDLWLCLSLDPQRRVTPPEESDYTHYAFSIS
EADFASFAARLEAAGVAVWKLNRSEGASHYFLDPDGHKLELHVGSLAQRLAACREQPYKGMVFFDQ
>GCA_000692955
MLSGLNHLTLAVSQLAPSVAFYQQLLGMTLHARWDSGAYLSCGDLWLCLSLDPQRRVTPPEESDYTHYAFSIS
EADFASFAARLEAAGVAIWKLNRSEGASHYFLDPDGHKLELHVGSLAQRLAACREQPYKGIVFFDE
>GCA_000349245
LSGLNHLTLAVSQLAPSVAFYQQLLGMTLHARWDSGAYLSCGDLWLCLSLDPQRRVTPPEESDYTHYAFSISE
ADFARFAARLEVADVAVWKLNRSEGASHYFLDPDGHKLELHVGSLAQRLAACREQPYKGMVFFDE
>GCA_900119335
MLSGLNHLTLAVSQLAPSVAFYRRLLGMTLHARWDSGAYLSCGDLWLCLSLDPQRRVTPPEESDYTHYAFSIS
EADFASFAARLEVAGVAVWKLNRSEGESHYFLDPDGHKLELHVGSLAQRLAACREQPYKGMVFIE
>GCA_001894545
MLQGLNHLTLAVSDLASSLAFYQQLPGMRLHASWDSGAYLSCGALWLCLSLDEQRRKTPPQESDYTHYAFSVA
EEEFAGVVALLAQAGAEVWKDNRSEGASYYFLDPDGHKLELHVGNLAQRLAACRERPYKGMVFVD
>GCA_001549975
LSGLNHLTLAVSQLAPSVAFYQQLLGMTLHARWDSGAYLSCGDLWLCLSLDPQRRITPLEESDYTHYAFSISE
ADFASFAARLEVAGVAVWKLNRSEGASHYVLDPDGHKLELHVGSLAQRLAACREQPYKGMVFFDQ
>GCA_900093395
MLSGLNHLTLAVSQLAPSVAFYQQLLGMTLHARWESGAYLSCGDLWLCLSLDPQRRITPPEESDYTHYAFSIS
EADFACFAARLEVAGVAVWKLNRSEGASHYFLDTDGHKLELHVGSLAQRLAACREQPYKGMVFFD
>GCA_900086385
LSGLNHLTLAVSQLAPSVAFYQQLLGMTLHARWDSGAYLSCGDLWLCLSLDPQRRITPSEESDYTHYAFSISE
ADFASFAARLEVAGVAVWKLNRSEGASHYFLDLDGHKLELHVGSLDQRLAACREQPYKGMVFFDQ
>GCA_000986855
LSGLNHLTLAVSQLAPSVAFYQQLLGMTLHARWDSGAYLSCGDLWLCLSLDPQRRITPSEESDYTHYAFSISE
ADFASFAARLEVAGVAVWKLNRSEGASHYFLDLDGHKLELHVGSLAQRLAACREQPYKGMVFFDQ
>GCA_000492415
MLSGLNHLTLAVSQLAPSVAFYQQLLGMTLHARWESGAYLSCGDLWLCLSLDPQRRITPPEESDYTHYAFSIS
EADFACFAARLEVAGVAVWKLNRSEGASHYFLDPDGHKLELHVGNLAQRLAACREQPYRGMVFFD
>GCA_001006625
LSGLNHLTLAVSQLAPSVAFYQQLLGMTLHARWDSGAYLSCGDLWLCLSLDPQRRITPSEESDYTHYAFSISE
ADFASFAARLEVAGVAVWKLNRSEGASHYFLDPDGHKLELHVGSLDQRLAACREQPYKGMVFFDQ
>GCA_001548315
LSGLNHLTLAVSQLAPSVAFYQQLLGMTLHARWDSGAYLSCGDLWLCLSLDPQRRVTPPEESDYTHYAFSISE
ADFARFVARLEVAGVAVWKLNRSEGASHYFLDPDGHKLELHVGSLAQRLAACREQPYKGMVFFDE
>GCA_000804345
MLQGLNHLTLAVSDLASSLAFYQRLPGMRLHARWDSGAYLSCGALWLCLSLDAQRRKTPAQESDYTHYAFSVA
EEHFAEVVAQLAHAGAEVWKDNRSEGASYYFLDPDGHKLELHVGHLAQRLAACRERPYKGMVFFD
>GCA_900084895

```
LSGLNHLTLAVSQLAPSVAFYQQLLGMTLHARWDSGAYLSCGDLWLCLSLDPQRRVTPPEKSDYTHYAFSISE
ADFARFAARLEVAGVAVWKLNRSEGASHYFLDPDGHKLELHVGSLAQRLAACREQPYKGMVFFDQ
>GCA_000496775
MLSTLNHLTLAVSDLARSVDFYHQLLGLKLHARWDNGAYLTCGDIWICLSVDEARRVTPAEQSDYTHYAFSIG
EGELAAFITRLEQAGVVSWKVNKSEGASYYFLDPDGHKLEAHVGDLAQRLAACRAKPYKGMVFF
>GCA_001059375
LKSINHICFSVRNLNDSIHFYRDILLGKLLFTGKKTAYFELAGLWIALNEEKDIPRNEIHFSYTHIAFTIDDS
EFKYWHQRLKDNNVNILEGRVRDIRDRQSIYFTDPDGHKLELHTGTLEDRLNYYKEAKPHMTFY
>GCA_000956895
MLSGLNHLTLAVSQLAPSVAFYHQLLGMTLHARWDSGAYLSCGDLWLCLSLDPQRRVTPPEESDYTHYAFSIS
EADFASFAARLEAAGVAVWKLNRSEGESHYFLDPDGHKLELHVGSLAQRLAACREQPYKGMVFF
>GCA_001631605
MLSGLNHLTLAVSQLAPSVAFYHQLLGMTLHARWDSGAYLSCGDLWLCLSLDPQRRVTPPEESDYTHYAFSIS
EADFASFAARLEVAGVAVWKLNRSEGESHYFLDPDGHKLELHVGSLAQRLAACREQPYKGMVFF
>GCA_000297835
MLTDLNHLTLAVNDVKKSFHFYVEVLGFKPLALWDHGAYLQLNTLWLCLSEDTRNITEISDYTHYAFSLSANN
FETFKQHLLSHGITSWKENKSEGDSFYFYDPDNHKLEIHVGDLESRLKTCRQYPYSGMQFFDNK
>GCA_001853385
MLLGINHLTIAVTNVEKSIYFYHHILGMHLHASWKNGAYLTCGELWVCLSFDPQRHHVDPDTTDYTHYAFSVT
ENDFSLVVEKLQQFDVKVWKDNRSEGASFYFLDPDGHKLEIHVGSLLDRLKSCLVHPYEEMKFY
>GCA_000158055
MLNGINHLTLAVTDLEKSIGFYQSLLGMKLHASWKKGAYISCGDLWLCLSLDITRQFSSPEKTDYTHYAFNVD
AKDFLIVVDRLMQANVIVWKENKSEGDSFYFLDPDGHKLELHVGGLLQRLKSCQEKPYEEMKFY
>GCA_001076695
MLTDLNHLTLAVNDVKKSFHFYVEVLGFKPLALWDHGAYLQLNTLWLCLSKDTRNITEISDYTHYAFSLSANN
FETFKQHLLSHGITSWKENKSEGDSFYFYDPDNHKLEIHVGDLESRLKTCRQYPYSGMQFFDNK
>GCA_000314835
MLNGINHLTLAVTDLDKSISFYQSLLGMKLYASWKKGAYISCGDLWLCLSLDTTRQFLSPEKTDYTHYAFNVD
AKDFLIVVDRLMQANVIVWKENKSEGDSFYFLDPDGHKLELHVGGLLQRLKSCQEKPYEEMKFY
>GCA_001680125
MLNGINHLTLAVTDLDKSISFYQSLLGMKLHASWKKGAYISCGDLWLCLSLDTTRQFLSPEKTDYTHYAFNVD
AKDFLIVVDRLMQANVIVWKENKSEGDSFYFLDPDGHKLELHVGGLLQRLKSCQEKPYEEMKFY
>GCA_001011625
MLSGLNHLTLAVSQLAPSVAFYHQLLGMTLHARWDSGAYLSCGDLWLCLSLDPQRRVTPPEESDYTHYAFSIS
EADFASFAARLEVAGVAVWKLNRSEGESYYFLDPDGHKLELHVGSLAQRLAACREQPYKGMVFF
>GCA_000530175
MLSGLNHLTLAVSQLAPSVAFYQQLLGMMLHARWDSGAYLSCGDLWLCLSLDPQRRVTPPEESDYTHYAFSIS
EADFASFAARLEAAGVAVWKLNRSEGASHYFLDPDGHKLELHVGSLAQRLAACREQPYKGDGVF
>GCA_000805715
MLVGINHLTIAVTDVEKSIFFYQSLLGMKLHASWKNGAYISCGDLWLCLSLDKTRLSFSHTETDYTHYAFTVS
EADFPICVAKLKQANVIVWKENKSEGKSFYFLDPDGHKLELHVGGLLQRLKSCQEAPYEGMKFY
>GCA_000020885
MLQSLNHLTLAVSNLQTSLTFWRDLLGLQLHAEWDTGAYLTCGDLWVCLSYDVSRNYVAPQESDYTHYAFSIA
PEDFEPFSCKLKQAGVTVWKDNKSEGQSFYFLDPDGHKLELHVGDLASRLTQCREKPYSGMRF
>GCA_001474655
MLQSLNHLTLAVSNLQTSLTFWRDLLGLQLHAEWDTGAYLTCGDLWVCLSYDVSRNYVAPQESDHTHYAFSIA
PEDFEPFSCKLKQAGVTVWKDNKSEGQSFYFLDPDGHKLELHVGDLASRLTQCREKPYSGMRF
>GCA_000190795
MLQSLNHLTLAVSNLQSSLTFWRDLLGLQLHAEWGTGAYLTCGDLWLCLSYDVSRSYVAPQKSDYTHYAFSIA
PEDFEPFSYKLKQSGVTVWKDNKSEGQSFYFLDPDGHKLELHVGDLASRLAQCRERPYSGMRF
>GCA_001474845
MLQSLNHLTLAVSKLQTSLTFWRDLLGLQLHAEWDTGAYLTCGDLWVCLSYDVSCNYVAPQECDYTHYAFSIA
PEDFEPFSYKLKQAGVTVWKDNKSEGQSFYFLDPDGHKLELHVGDLASRLAQCREKPYSGMRF
>GCA_000020705
```

MLQSLNHLTLAVSNLQTSLTFWRDLLGLQLHAEWDTGAYLTCGDLWVCLSYDVSCNYVAPQECDYTHYAFSIA
PEDFEPFSYKLKQAGVTVWKDNKSEGQSFYFLDPDGHKLELHVGDLASRLAQCREKPYSGMRF
>GCA_000487235
MLQSLNHLTLAVSNLQTSLTFWRDLLGLQLHVEWDTGAYLTCGDLWVCLSYDVSRNYVAPQESDYTHYAFSIA
PEDFEPFSYKLKQAGVTVWKDNKSEGQSFYFLDPDGHKLELHVGDLASRLTQCRERPYSGMRF
>GCA_001903595
MLSGLNHLTLAVSRLAPSVAFYHQLLGMTLHARWDGGAYLACGDLWLCLSLDPQRRITPPEESDYTHYAFSIA
EADFSGVAARLEVAGVTMWKMNRSEGASYYFLDPDGHKLELHVGSLAQRLAACREQPYKGMVF
>GCA_001309675
VIQSLNHLTLAVSNLQSSLTFWRDLLGLQLHAEWGTGAYLTCGDLWLCLSYDVSRSYVAPQKSDYTHYAFSIA
PEDFEPFSYKLKQSGVTVWKDNKSEGQSFYFLDPDGHKLELHVGDLASRLAQCRERPYSGMRF
>GCA_001880915
MLQSLNHLTLAVSNLQTSLTFWRDLLGLQLHAEWETGAYLTCGDLWICLSYDVSRNYVAPQESDYTHYAFSIT
PEDFESFSYKLKQSGVTVWKENKSEGQSFYFLDPDGHKLELHVGDLASRLAQCREKPYSGMRF
>GCA_001569625
MLQSLNHLTLAVSNLQTSLTFWRDLLGLQLHAEWDTGAYLTCGDLWVCLSYDVSRNYVAPQESDYTHYAFSIA
PEDFEPFSCKLKQAGVTVWKDNKSEGQSFYFLDPDGHKLELHVGDLASRLTQCRERPYSGMRF
>GCA_001034065
LSGLNHLTLAVSQLAPSVAFYQQLLGMTLHARWDSGAYLSCGDLWLCLSLDPQRRITPSEESDYTHYAFSISE
ADFASFAARLEVAGVAVWKLNRSEGASHYFLDPDGHKLELHVGSLDQRLAACREQPYKGMVFL
>GCA_001537405
MLQSLNHLTLAVSNLQSSLAFWRDLLGLQLHAEWDTGAYLTCGDLLICLSYDVSRNYVAPRESDNTHYAFSIA
PEDFEPFSYKLKQSEVMVWKANKSEGQSFYFLDPDGHKLELHVGDLASRLAQCMAKPYSGMLF
>GCA_000486835
MLQSLNHLTLAVSNLQTSLTFWRDLLGLQLHAEWDTGAYLTCGDLWVCLSYDVSRNYVAPQESDYTHYAFSIA
PEDFEPFSCKLKQAGVTVWKDNKSEGQSFYFLDPDGHKLELHVGDLASRLAQCREKPYSGMRF
>GCA_000529745
ILQSLNHLMLAVSNLQSSLTFRDLLGLQLHAEWDTGAYLTCGDLWICLSYDVSRNYVATQEGDYTHYAFSIAP
EDFESFSYKLKQAGVTVWKDNKSEGQSFYFLDPDGHKSELHVGDLASRLVQCRERLYSGMRF
>GCA_000791765
MFTGLNHLTLAVADLPASIAFYRDLLGFRLEARWDQGAYLELGSLWLCLSREPQYGGPVADYTHYAFGIAAAD
FARFAAQLRAHGVREWKQNRSEGDSFYFLDPDGHRLEAHVGDLRSRLAACRQAPYAGMRFAD
>GCA_001902635
MLTGLNHLTLAVSDLDRSFDFYHRLLGFTPHARWQGGGYLSLGALWLCLSLDEARTQQSARDYTHYAFSVAPE
HIERVSERLRQNGVEEWKSNRSEGESLYFLDPDGHQLEIHAGDLASRLAACREKPYQGMVFY
>GCA_000215775
MLTGLNHLTLAVADLPASIAFYRDLLGFRLEARWDQGAYLELGSLWLCLSREPQCGGPAADYTHYAFGIAAAD
FARFAAQLRAHGVREWKQNRSEGDSFYFLDPDGHRLEAHVGDLRSRLAACRQAPYAGMRFAD
>GCA_001554535
MLTGLNHLTLAVADLPASIAFYRDLLGFRLEARWDQGAYLELGSLWLCLSREPQCGGPAADYTHYAFGIAAAD
FARFAAQLRAHGVREWKQNRSEGDSFYFLDPDGHRLEAHVGDLRSRLAARRQAPYAGMRFAD
>GCA_900146525
MLTGLNHLTLAVADLPASIAFYRDLLGFRLEARWDQGAYLELGSLWLCLSREPQYGGPAADYTHYAFGIAAAD
FARFAAQLRAHGVREWKQNRSEGDSFYFLDPDGHRLEAHVGDLRSRLAACRQASYAGMRFAN
>GCA_000342205
MLTGLNHLTLAVSNLDRSFDFYRHLLGFIPHARWQGGAYLSLGPLWLCLSLDEARMQQRERDYPHYAFSVAPE
HIEQVSERLRQAGVEEWKSNRSEGESLYFLDPDGHQLEIHAGDLASRLAACREKPYQGMVFY
>GCA_001432595
MKLSGLNHLTISVANVDRIFNFYKDILGFTPKAKWKKGAYLSLGELWLCLSLDEVSVSSDYTHYCLSISEDDI
NEFRQKIKLLNIREWKNNQSEGESIYFLDPDGHKLEVHVGNLSTRLESCRKNPYEGMVFFDE
>GCA_000783975
MLTGLNHLTLAVSDLDRSFDFYRHLLGFTPHARWQGGAYLSLGTLWLCLSLDEARTQPRARDYTHYAFSVAPE
HIERVSERLRQSGAEEWKSNRSEGESLYFLDPDGHQLEIHAGDLASRLAACREKPYQGMVFY
>GCA_001453145

MLTGLNHLTLAVAELPASIAFYRDLLGFRLEARWDQGAYLELGSLWLCLSREPQYGGPAADYTHYAFGIAAAD
FARFAAHLRAHGVREWKQNRSEGDSFYFLDPDGHRLEAHVGDLRSRLAACRQAPYAGMRFAD
>GCA_000793005
MLTGLNHLTLAVADLPASIAFYRDLLGFRLEARWDQGAYLELGSLWLCLSREPQYGGPAADYTHYAFGIAAAD
FARFAAHLRAHGVREWKQNRSEGDSFYFLDPDGHRLEAHVGDLRSRLAACRQAPYAGMRFAD
>GCA_000783275
MLTGLNHLTLAVADLPVSIAFYRDLLGFRLEARWDQGAYLELGSLWLCLSREPQYGGPAADYTHYAFGIAAAD
FARFAAHLRAHGVREWKQNRSEGDSFYFLDPDGHRLEAHVGDLRSRLAACRQAPYAGMRFAD
>GCA_001536545
MLTGLNHLTLAVSDLDRSFDFYRHLLGFTPHARWQGGAYLSLGPLWLCLSLDERRTQQRERDYTHYAFSVAPE
HIEQASQRLRQAGVEEWKSNRSEGESLYFLDPDGHQLEIHAGDLASRLAACREKPYQGMVFY
>GCA_001537565
MLTGLNHLTLAVSDLDRSFDFYRHLLGFTPHARWQGGAYLSLGPLWLCLSLDERRTQQRERDYTHYAFSIAPE
HIEQASQRLRQAGVEEWKSNRSEGESLYFLDPDGHQLEIHAGDLASRLAACREKPYQGMVFY
>GCA_000297575
MKLSGLNHLTISVANVDRSFNFYKDILGFTPKAKWKKGAYLSLGKLWLCLSLDEVSVSSDYTHYCLSISEDDI
DEFRQKIKLLNIREWKNNQSEGESIYFLDPDGHKLEVHVGNLSTRLESCRKNPYEGMVFFDE
>GCA_001536585
MLTGLNHLTLAVSDLDRSFDFYRHLLGFTPHARWQGGAYLSLGPLWLCLSLDEKRTQQHERDYTHYAFSVAPE
HIEQASQRLRQAGVEEWKSNRSEGESLYFLDPDGYQLEIHAGDLASRLAACREKPYQGMVFY
>GCA_001077565
MKLSGLNHLTISVANVDRSFNFYKDILGFTPKAKWKKGAYLSLGELWLCLSLDEVSVSSDYTHYCLSISEDDI
DEFRQKIKLLNIREWKNNQSEGESIYFLDPDGHKLEVHVGNLSTRLESCRKNPYEGMVFFDE
>GCA_000793665
MLTGLNHLTLAVADLPASIAFYRDLLGFRLEARWDQGAYLELGSLWLRLSREPQYGGPAADYTHYAFGIAAAD
FARFAAQLRAHGVREWKQNRSEGDSFYFLDPDGHRLEAHVGDLRSRLAACRQAPYAGMRFAD
>GCA_001547955
MLTGLNHLTLAVADLPASIAFYRDLLGFRLEARWDQGAYLELGSLWLCLSREPQYGGPAADYTHYAFGIAAAD
FARFAAQLRAHGVREWKQNRSEGDSFYFLDPDGHRLEAHVGDLRSRLAACRQAPSAGMRFAD
>GCA_000629605
MLTGLNHLTLAVADLPASIAFYRDLLGFRLEARWDQGAYLELGSLWLCLSREPQYGGPAADYTHYAFGIAAAD
FARFAAQLRAHGVREWKQNRSEGNSFYFLDPDGHRLEAHVGDLRSRLAACRQAPYAGMRFAD
>GCA_000796065
MLTGLNHLTLAVADLPASIAFYRDLLGFRLEARWDQGAYLELGSLWLCLSREPQYGGPAADYTHYAFGIAAAD
FARFAAQLRAHGVREWKQNRSEGDSFYFLDPDGHRLEAHVGDLRSRLAACRQAPYAGMRFVD
>GCA_001060335
MLTGLNHLTLAVSDLDRSFDFYRHLLGFTPHARWQGGAYLSLGALWLCLSRDEARAQQSARDYTHYAFSVAPD
HIEQVSERLRQSGVKEWKSNRSEGESLYFLDPDGHQLEIHASDLASRLAACREKPYQGMVFY
>GCA_001063125
MLTGLNHLTLAVSNLDRSFDFYRHLLGFIPHARWQGGAYLSLGPLWLCLSLDEARMQQRERDYTHYAFSVAPE
HIEQVSERLRQAGVEEWKSNRSEGESLYFLDPDGHQLEIHAGDLASRLAACREKPYQGMVFY
>GCA_001065405
MLTGLNHLTLAVSNIDRSFDFYRHLLGFIPHARWQGGAYLSLGPLWLCLSLDETRMQQRERDYTHYAFSVAPE
HIEQVSERLRQAGVEEWKSNRSEGESLYFLDPDGHQLEIHAGDLASRLAACREKPYQDMVFY
>GCA_001889685
MLTGLNHLTLAVSDLDRSFDFYRHLLGFTPHARWQGGAYLSLGALWLCLSLDEARTQPRARDYTLYAFSVAPE
HIERVSERLRQSGAEEWKSNRSEGESLYFLDPDGHQLEIHAGDLASRLAACREKPYQGMVFY
>GCA_001454395
MLTGLNHLTLAVADLPASIAFYRDLLGFRLEARWDQGAYLELGSLWLCLSREPQYGGPAADYTHYAFGIAAAD
FARFAAQLRAHGVREWKQNRSEGDSFYFLDPDGHRLEAHVGDLRSRLAACRQAPYVGMRFAD
>GCA_000633715
MLTGLNHLTLAVSDLDRSFDFYRHLLGFTPHARWQGGAYLSLGPLWLCLSLDEKRTQQHERDYTHYAFSVAPE
HIEQASQRLRQAGVEEWKSNRSEGESLYFLDPDGHQLEIHAGDLASRLAACREKPYQGMVFY
>GCA_000468935

MLTGLNHLTLAVADLPASIAFYRDLLGFRLEARWDQGAYLELGSLWLCLSREPQYGGPAADYTHYAFGIAAAD
FARFAAQPRAHGVREWKQNRSEGDSFYFLDPDGHRLEAHVGDLRSRLAACRQAPYAGMRFAD
>GCA_000148745
MLTGLNHLTLAVADLPASIAFYRDLLGFRLEARWDQGAYLELGSLWLCLSREPQYGGPAADYTHYAFGIAAAD
FARFAAQLRAHGVREWKQNRSEGDSFYFLDPDGHRLEAHVGDLRSRLAACRQAPYAGMRFAN
>GCA_000797285
MLTGLNHLTLAIADLPASIAFYRDLLGFRLEARWDQGAYLELGSLWLCLSREPQYGGPAADYTHYAFGIAAAD
FARFAAQLRAHGVREWKQNRSEGDSFYFLDPDGHRLEAHVGDLRSRLAACRQAPYAGMRFAD
>GCA_000581995
MKLSGLNHLTISVANVDRSFNFYKDILDFTPKAKWKKGAYLSLGELWLCLSLDEVSISSDYTHYCFSISEDNI
DEFRQKIKMMNIREWKNNQSEGESIYFLDPDGHKLEVHVGNLSTRLESCRKNPYEGMVFFDE
>GCA_000738535
MLTGLNHLTLAVSDLDRSFDFYRHLLGFTPHARWQGGAYLSLGALWLCLSLDEARMLPRERDYTHYAFSVAPK
HIERVSERLRQAGVEEWKSNRSEGESLYFLDPDGHQLEIHAGDLASRLAACREKPYQGMVFY
>GCA_001007555
MLTGLNHLTLAVSDLDRSFDFYRHLLGFTPHARWQGGAYLSLGALWLCLSLDEARTPPRARDYTHYAFSVAPE
HIERVSERLRQSGAEEWKSNRSEGESLYFLDPDGHQLEIHAGDLASRLAACREKPYQGMVFY
>GCA_000418815
MLTGLNHLTLAVSDLDRSFEFYRHLLGFTPHARWQGGAYLSLGALWLCLSRDEARAQQSARDYTHYAFSVAPE
HIEQVSERLRQNGVKEWKSNRSEGESLYFLDPDGHQLEIHAGDLASRLAACREKPYQGMVFY
>GCA_001672055
MLTGLNHLTLAVSNLDRSFDFYRHLLGFIPHARWQGGAYLSLGPLWLCLSLDETRMQQHERDYTHYAFSVAPE
HIEQVSERLRQAGVEEWKSNRSEGESLYFLDPDGHQLEIHAGDLASRLAAYREKPYQGMVFY
>GCA_000341565
MLTGLNHLTLAVADLPASIAFYRDLLGFRLEARWDQGAYLELGSLWLCLSREPQYGGPAADYTHYAFGIAAAD
FARFAAQLRAHGVREWKQNRSEGDSFYFLDPDGHRLEAHVGDLLSRLAACRQAPYAGMRFAD
>GCA_001444845
MLTGLNHLTLAVADLPASIAFYRDLLGFRLEARWDQGAYLELGSLWLCLSREPQYGGPAADYTHYAFGIAAAD
FARFAAQLRAHGVREWKQNRNEGDSFYFLDPDGHRLEAHVGDLRSRLAACRQAPYAGMRFAD
>GCA_000791705
MLTGLNHLTLAVADLPASIAFYRDLLGFRLEARWDQGAYLELGSLWLCLSREPQYGGPAADYTHYAFGIAAAD
FARFAAHLRAHGVREWKQNRSEGDSFYFLDPDGHRLEAHVGDLRSRLSACRQAPYAGMRFAD
>GCA_000794725
MLTGLNHLTLAVADLPASIAFYRDLLGFRLEARWDQGAYLELGSLWLCLSREPQYGGPAADYTHYAFGIAAAD
FARFAAQLRAHGVREWKQNRSEGDSFYFLDPDGHRLEAHVGDLRSRLAAFRQAPYAGMRFAD
>GCA_001449435
MLTGFNHLTLAVADLPASIAFYRDLLGFRLEARWDQGAYLELGSLWLCLSREPQYDGPAEDYTHYAFGIAAAD
FARFAAHLRGHGVREWKQNRSEGDSFFLDPDGHRLEAHVGDLRSRLAACRQTPYAGMRFAD
>GCA_001294565
MLTGLNHLTLAVSDLDRSFDFYRHLLGFTPHARWQGGAYLSLGSLWLCLSLDERRTQQHERDYTHYAFSVAPE
HIEQASQQLRQAGVEEWKSNRSEGESLYFLDPDGHQLEIHAGDLASRLAACREKPYQGMVFY
>GCA_001537145
MLTGLNHLTLAVSDLDRSFDFYRHLLGFTPHARWQGGAYLSLGPLWLCLSLDEKRTQQHERDYTHYAFSVAPE
HIEQASQRLRQAGVKEWKSNRSEGESLYFLDPDGHQLEIHAGDLASRLAACREKPYQGMVFY
>GCA_000647635
MLTGLNHLTLAVADLPASIAFYRDLLGFRLEARWDQGAYLELGSLWLCLSREPQYGGPAADYTHYAFGIAAAD
FARFAAQLRAHGVREWRQNRSEGDSFYFLDPDGHRLEAHVGDLRSRLAACRQAPYAGMRFAD
>GCA_000506005
MLTGLNHLTLAVADLPASIAFYRDLLGFRLEARWDQGAYLELGSLWLCLSRDPQYGGPAADYTHYAFGIAAAD
FARFAAQLRAHGVREWKQNRSEGDSFYFLDPDGHRLEAHVGDLRSRLAACRQAPYAGMRFAD
>GCA_000481865
MLTGLNHLTLAVADLPASIAFYRDLLGFRLEARWDKGAYLELGSLWLCLSREPQYGGPAADYTHYAFGIAAAD
FARFAAQLRAHGVREWKQNRSEGDSFYFLDPDGHRLEAHVGDLRSRLAACRQAPYAGMRFAD
>GCA_000408865

MLTGLNHLTLAVADLPASIAFYHDLLGFRLEARWDQGAYLELGSLWLCLSREPQYGGPAADYTHYAFGIAAAD
FARFAAQLRAHGVREWKQNRSEGDSFYFLDPDGHRLEAHVGDLRSRLAACRQAPYAGMRFAD
>GCA_000481205
MLTGLNHLTLAVADLPASIAFYRDLLGFRLEARWDQGAYLELGSLWLCLSREPQYGGPAADYTHYAFGIAAAD
FARFAAQLRAHGVREWKQNRSEGDSFYFLDPDGHRLEVHVGDLRSRLAACRQAPYAGMRFAD
>GCA_001067375
MLTGLNHLTLAVSDLDRSFDFYRHLLGFTPHARWQGGAYLSLGSLWLCLSLDERRTQQRERDYTHYAFSIAPE
HIEQASQRLRQAGVKEWKSNRSEGESLYFLDPDGHQLEIHAGDLASRLAACREKPYQGMVFY
>GCA_000521905
MLTGLNHLTLAVSDLDRSFDFYRHLLGFTPHARWQGGAYLSLGALWLCLSLDEARTQPRARDYTHYAFSVAPE
HIERVSERLRQSGAEEWKSNRSEGESLYFLDPDGHQLEIHAGDLASRLAACREKPYQGMVFY
>GCA_000359505
MLTGLNHLTLAVADLPASIAFYRDLLGFRLEARWDQGAYLELGSLWLCLSREPQYGGPAADYTHYAFGIATAD
FARFAAQLRAHGVREWKQNRSEGDSFYFLDPDGHRLEAHVGDLRSRLAACRQAPYAGMRFAD
>GCA_000783915
MLTGLNHLTLAVSDLDRSFDFYRHLLGFTPHARWQGGAYLSLGPLWLCLSLDERRTQQHERDYTHYAFSVAPE
HIEQASQRLRQAGVEEWKSNRSEGESLYFLDPDGHQLEIHAGDLASRLAACREKPYQGMVFY
>GCA_001068085
MLTGLNHLTLAVSDLDRSFDFYRHLLGFTPHARWQGGAYLSLGALWLCLSRDEARAQQSARDYTHYAFSVAPD
HIEQVSERLRQNGVKEWKSNRSEGESLYFLDPDGHQLEIHAGDLASRLAACREKPYQGMVFY
>GCA_001578415
MLTGLNHLTLAVADLPASIAFYRDLLGFRLEARWDQGAYLELGSLWLCLSREPQYGGPAADYTHAFGIAAADF
ARFAAQLRAHGVREWKQNRSEGDSFYFLDPDGHRLEAHVGDLRSRLAACRQAPYAGMRFAD
>GCA_000481065
MLTGLNHLTLAVADLPASIAFYRDLLGFRLKARWDQGAYLELGSLWLCLSREPQYGGPAADYTHYAFGIAAAD
FARFAAQLRAHGVREWKQNRSEGDSFYFLDPDGHRLEAHVGDLRSRLAACRQAPYAGMRFAD
>GCA_001066075
MLTGLNHLTLAVADLPASIAFYRDLLGFRLEARWDQGAYLELGSLWLCLSREPQCGGPAADYTHYAFGIAAAD
FARFAAQLRAHGVREWKQNRSEGDSFYFLDLDGHRLEAHVGDLRSRLAACRQAPYAGMRFAD
>GCA_000292365
MLTGLNHLTLAVHDLDRSVDFYHHLLGFIPHARWQGGAYLSLGALWLCLSVDESRTPQNARDYTHYAFSVAPE
HMEQVSERLRQSGVEEWKSNRSEGESLYFLDPDGHQLEIHAGDLASRLAACREIPYQGMVFY
>GCA_000521925
MLTGLNHLTLAVSDLDRSFDFYRHLLGFTPHARWQGGAYLSLGSLWLCLSLDERRTQQHERDYTHYAFSVAPE
HIEQASQRLRQAGVEEWKSNRSEGESLYFLDPDGHQLEIHAGDLASRLAACREKPYQGMVFY
>GCA_001060585
MLTGLNHLTLAVSDLDRSFDFYRHLLGFTPHARWQGGAYLSLGPLWLCLSLDERRTQQRERDYTHYAFSVAPE
HIEQASQRLRQAGIEEWKSNRSEGESLYFLDPDGHQLEIHAGDLASRLAACREKPYQGMVFY
>GCA_000481405
MLTGLNHLTLAVADLPASIAFYRDLLGFRLEARWDQGAYLELGSLWLCLSREPQYGGPAADYTHYVFGIAAAD
FARFAAQLRAHGVREWKQNRSEGDSFYFLDPDGHRLEAHVGDLRSRLAACRQAPYAGMRFAD
>GCA_001750225
MLSGLNHLTLAVADLPASIAFYRDLLGFRLEARWDQGAYLELGSLWLCLSREPQYGGPAADYTHYAFGIAAAD
FARFAAQLRAHGVREWKQNRSEGDSFYFLDPDGHRLEAHVGDLRSRLAACRQAPYAGMRFAD
>GCA_001643155
MLTGLNHLTLAVSDLDRSVEFYRHLLGFTPHARWQGGAYLSLGALWLCLSLDESRTQQRERDYTHYAFSVAPE
HIERVSERLRQNGVKEWKSNRSEGESLYFLDPDGHQLEIHAGDLASRLAACREKPYQGMVFY
>GCA_000258285
MLTGLNHLTLAVADLPASIAFYRDLLGFRLEARWDQGAYLELGSLWLCLSREPQCGGPAADYTHYAFGIAAAD
FARFAAQLRAHGVREWKQNRSEGDSFYFLDPDGHRLAHVGDLRSRLAACRQAPYAGMRFAD
>GCA_001756295
MLTGLNHLTLAVSNLDRSFDFYRHLLGFIPHARWQGGAYLSLEPLWLCLSLDETRMQQRERDYTHYAFSVAPE
HIEQVSERLRQAGVEEWKSNRSEGESLYFLDPDGHQLEIHAGDLASRLAACRKKPYQGMVFY
>GCA_000783615

MLTGLNHLTLAASDLDRSFEFYRHLLGFTPHARWQGGAYLSLGALWLCLSRDEARAQQSARDYTHYAFSVAPQ
HIEQVSERLRQNGVKEWKSNRSEGESLYFLDPDGHQLEIHAGDLASRLAACREKPYQGMVFY
>GCA_001975745
MLIGLNHLTLAVSNLDRSFDFYRHLLGFIPHARWQGGAYLSLGPLWLCLSLDETRMQQRERDYTHYAFSVAPE
HIEQVSERLRQAGVEEWKSNRSEGESLYFLDPDGHQLEIHAGDLASRLAACREKPYQGMVFY
>GCA_000264275
MLTGLNHLTLAVSNLDRSFDFYRHLLGFIPHARWQGGAYLSLGPLWLCLSLDETRMQQRERDYTHYAFSVAPE
HIEQVSERLRQAGVEEWKSNRSEGESLYFLDPDGHQLEIHAGDLASRLAACREKPYQGMVFY
>GCA_000756575
MLTGLNHLTLAVADLPASIAFYRDLLGFRLEARWDQGAYLELGSLWLCLSREPQCGGPAADYTHYAFGIDAAD
FARFAAQLRAHGVREWKQNRSEGDSFYFLDPDGHRLEAHVGDLRSRLAACRQAPYAGMRFAD
>GCA_001909165
MLTGLNHLTLAVQDLDRSVDFYHHLLGFIPHARWQGGAYLSLGALWLCLSVDESRTPQNARDYTHYAFSVAPE
HMEQVSERLRQSGVEEWKSNRSEGESLYFLDPDGHQLEIHAGDLASRLAACRENPYQGMVFY
>GCA_001065325
MLTGLNHLTLAVSNLDRSFDFYRHLLGFIPHARWQGGAYLSLGPLWLCLSLDETRMQQRERDYTHYAFSVAPE
HIEQVSERLRQAGVEEWKSNRSEGESLYFLDPDGHQLEIHAGDLASRLAACRKKPYQGMVFY
>GCA_000792095
MLTGLNHLTLAVADLPASIAFYRDLLGFRLEARWDQGAYLELGSLWLCLSREPQYGGPAADYTHYAFGIAAAD
FARFTAQLRAHGVREWKQNRSEGDSFYFLDPDGHRLEAHVGDLRSRLAACRQAPYAGMRFAD
>GCA_000791825
MLTGLNHLTLAVADLPASIAFYRDLLGFRLEARWDQGAYLELGSLWLCLSREPQYGGPAADYTHYAFGIAAAD
FARFAAQLRAHGVREWKQNRSEGDSFYFLDSDGHRLEAHVGDLRSRLAACRQAPYAGMRFAD
>GCA_000506285
MLTGLNHLTLAVADLPASIAFYRDLLGFRLEARWDQGAYLELGSLWLCLSREPQCDGPAADYTHYAFGIAAAD
FARFAAQLRAHGVREWKQNRSEGDSFYFLDPDGHRLAHVGDLRSRLAACRQAPYAGMRFAD
>GCA_001061875
MLTGLNHLTLAVADLPARIAFYRDLLGFRLEARWDQGAYLELGSLWLCLSREPQYGGPAADYTHYAFGIAAAD
FARFAAQLRAHGVREWKQNRSEGDSFYFLDPDGHRLEAHVGDLRSRLAACRQAPYAGMRFAD
>GCA_000369025
MKLSGLNHLTISVANVDRSFSFYKDVLGFTPRAKWNKGAYLSLGELWLCLSLDEVSITSDYTHYCFSISEDHI
DAFRQKIKLLNIREWKNNQSEGESIYFLDPDGHKLEVHLGNLSSRLESCKKNPYEGMVFFDE
>GCA_000790655
MLTGLNHLTLAVADLPASIAFYRDLLGFRLEARWDQGAYLELGSLWLCLSREPQYGGPAADYMHYAFGIAAAD
FARFAAQLRAHGVREWKQNRSEGDSFYFLDPDGHRLEAHVGDLRSRLAACRQAPYAGMRFAD
>GCA_000790685
MLTGLNHLTLAVADLPASIAFYRDLLGFRLEARWDQGAYLELGSLWLCLSREPQYGGPAADYTHCAFGIAAAD
FARFAAQLRAHGVREWKQNRSEGDSFYFLDPDGHRLEAHVGDLRSRLAACRQAPYAGMRFAD
>GCA_000186665
MKLSGLNHLTISVANVDRSFNFYKDILGFTPKAKWKKGAYLSLGELWLCLSLDEVSISSDYTHYCFSISEDNI
DEFRQKIKMMNIREWKNNQSEGESIYFLDPDGHKLEVHVGNLSTRLESCRKNPYEGMVFFDE
>GCA_001554585
MLTGLNHLTLAVADLPASIAFYRDLLGFRLEARWDQGAYLELGSLWLCLSREPQYGGPAADYAHYAFGIAAAD
FARFAAQLRAHGVREWKQNRSEGDSFYFLDPDGHRLEAHVGDLRSRLAACRQAPYAGMRFAD
>GCA_001431165
MLTGLNHLTLAVADLPASIAFYRDLLGFRLEARWDQGAYLELGSLWLCLSQEPQYGGPAADYTHYAFGIAAAD
FARFAAHLRAHGVREWKQNRSEGDSFYFLDPDGHRLEAHVGDLRSRLAACRQAPYAGMRFAD
>GCA_000481185
MLTGLNHLTLAVADMPASIAFYRDLLGFRLEARWDQGAYLELGSLWLCLSREPQYGGPAADYTHYAFGIAAAD
FARFAAQLRAHGVREWKQNRSEGDSFYFLDPDGHRLEAHVGDLRSRLAACRQAPYAGMRFAD
>GCA_000226155
MLTGLNHLTLAVAVLPASIAFYRDLLGFRLEARWDQGAYLELGSLWLCLSREPQYGGPAADYTHYAFGIAAAD
FARFAAQLRAHGVREWKQNRSEGDSFYFLDPDGHRLEAHVGDLRSRLAACRQAPYAGMRFAD
>GCA_000796505

MLTGLNHLTLAVADLPASIAFYRDLLGFRLEARWDQGAYLELGSLWLCLSREPQYGRPAADYTHYAFGIAAAD
FARFAAQLRAHGVREWKQNRSEGDSFYFLDPDGHRLEAHVGDLRSRLAACRQAPYAGMRFAD
>GCA_001415145
MKLSGLNHLTISVENVDRSFSFYKDVLGFTPRAKWNKGAYLSLGELWLCLSLDEVRITSDYTHYCFSISEDDI
DEFRQKIKLLNIREWKNNQSEGESIYFLDPNGHKLEIHVGNLSTRLDSCKKNPYEGMVFFDE
>GCA_900083925
MLTGLNHLTLAVSDLDRSFDFYRHLLGFTPHARWQGGAYLSLGALWLCLSRDEARAQQSARDYTHYAFSVAPD
HIEQVSERLRQSGVKEWKSNRSEGESLYFLDPDGHQLEIHAGDLASRLAACREKPYQGMVFY
>GCA_900144765
MLTGLNHLTLAVAELPASIAFYRDLLGFRLEARWDQGAYLELGSLWLCLSREPQYGGPAADYTYYAFGIAAAD
FARFAAHLRAHGVHEWKQNRSEGDSFYFLDPDGHRLEAHVGDLRSRLAACRQAPYAGMRFAD
>GCA_001536365
MLTGLNHLTLAVSDLDRSFDFYRHLLGFTPHARWQGGAYLSLGSLWLCLSLDERRTQQRERDYTHYAFSVAPE
HIEQASQRLRQAGVEEWKSNRSEGESLYFLDPDGHQLEIHAGDLASRLAACREKPYQGMVFY
>GCA_001066015
MLTGLNHLTLAVSDLDRSFDFYRHLLGFTPHARWQGGAYLSLGSLWLCLSLDERRTQQHERDYTHYAFSIAPE
HIEQASQRLRQAGVEEWKSNRSEGESLYFLDPDGHQLEIHAGDLASRLAACREKPYQGMVFY
>GCA_000714515
MLTGLNHLTLAVADLPASIAFYRDLLGFRLEARWDQGAYLELGSLWLCLSREPGGPAADYTHAFGIAAADFAR
FAAQLRAHGVREWKQNRSEGDSFYFLDPDGHRLAHVGDLRSRLAACRQAPYAGMRFAD
>GCA_001539985
MLTGLNHLTLAVSNLDRSFDFYRHLLGFIPHARWQGGAYLSLGPLWLCLSLDETRMQQRERDYTHYAFSVAPE
HIEQVSERLRQAGVEEWKNNRSEGESLYFLDPDGHQLEIHAGDLASRLAACREKPYQGMVFY
>GCA_000568235
MLTGFNHLTLAVADLPASIAFYRDLLGFRLEARWDQGAYLELGSLWLCLSREPQYDGPAEDYTHYAFGIAAAD
FARFAAHLRGHGVREWKQNRSEGDSFYFLDPDGHRLEAHVGDLRSRLAACRQTPYAGMRFAD
>GCA_000399685
MKLSGLNHLTISVENVDRSFSFYKDVLGFTPRAKWNKGAYLSLGELWLCLSLDEVSISSDYTHYCFSISEDDI
DEFRQKIKLMNIHEWKNNQSEGESIYFLDPDGHKLEVHVGTLSSRLQSCKKNPYEGMVFFDE
>GCA_000399705
MKLSGLNHLTISVENVDRSFSFYKDVLGFSPRAKWNKGAYLSLGELWLCLSLDEVSISSDYTHYCFSISEDHI
DEFRQKIKLLNIREWKNNQSEGESIYFLDPDGHKLEVHVGTLSSRLKSCKKNPYEGMVFFN
>GCA_900148255
MLTGLNHLTLAVADLPASIAFYRDLLGFRLEARWDQGAYLELGSLWLCLSREPQYGGPAADYTHYAFGIAAAD
FARFAAQLRAHGVREWKQNRSEGDSFYFLDPDGHRLEAHVGDLRSRLAACRQAPYAGMRFA
>GCA_000588275
MKLSGLNHLTISVENVDRSFSFYKDVLGFTPRAKWNKGAYLSLGELWLCLSLDEVSISSDYTHYCFSISEDDI
DEFRQKIKLLNIREWKNNQSEGESIYFLDPDGHKLEVHVGNLSSRLESCKKNLYEGMIFF
>GCA_900144795
MLTGLNHLTLAVAELPASIAFYRDLLGFRLEARWDQGAYLELGSLWLCLSREPQYGGPAADYTYYAFGIAAAD
FARFAAHLRAHGVHEWKQNRSEGDSFYFLDPDGHRLEAHVGDLRSRLAACRQAPYAGMRF
>GCA_000480725
MLTGLNHLTLAVADLPASIAFYRDLLGFRLEARWDQGAYLELGSLWLCLSREPQYGGPAADYTHYAFGIAAAD
FARFAAQLRAHGVREWKQNRSEGDSFYFLDPDGHRLEAHVGDLRSRLAACRQAPYAGMRF
>GCA_001867255
LTGLNHITIAANDISRSFNFYVEILGFTPKARWATGAYLCLGDLWLCLSAEKVSSRGDYTHYAFSIQSEDFDR
FANHLKSCGITEWKSNKSEGKSIYLLDPDGHKLEIHDGDLVSRLNSCMKHPYEDMIFFN
>GCA_000191145
MKLSGLNHLTISVENVDRSFSFYKDVLGFTPRAKWNKGAYLSLGELWLCLSLDEVRITSDYTHYCFSISEDDI
DEFRQKIKLLNIREWKNNQSEGESIYFLDPNGHKLEVHVGNLSTRLDSCKKNPYEGMV
>GCA_000831565
TTSVSNLQSSLTFWRDLLGLQLHAEWGTGAYLTCGDLWLCLSYDVSRSYVAPQKSDYTHYAFSIAPEDFEPFS
YKLKQSGVTVWKDNKSEGQSFYFLDPDGHKLELHVGDLASRLAQCRERPYSGMRF
>GCA_000633025

MLSGLNHLTLAVSQLAPSVAFYQQLLGMTLHARWDSGAYLSCGDLWLCLSLDPQRRVTPPEESDYTHYAFSIS
EADFASFAARLEAAGVAIWKLNRSEGASHYFLDPDGHKLELHVGSLAQRLAACRE
>GCA_000791025
AYRTSPVGRLRLNVPRDASRLLLGPVLRRFTEAYPDLQLELSVEDRMVDIVADGYDAGIRYGGTVPQDMVAVP
LTGELRWVWKQNRSEGDSFYFLDPDGHRLEAHVGDLRSRLAACRQAPYAGMRF
>GCA_000412735
MLTGLNHLTLAVADLPASIAFYRDLLGFRLEARWDQGAYLELGSLWLCLSREPQYGGPAADYTHYAFGIAAAD
FARFAAQLRAHGVREWKQNRSEGDSFYFLDPDGHRLEAHVGDLRSRLAACRQAP
>GCA_000372625
MLSGLNHLTLAVSQLAPSVAFYQQLLGMTLHARWDSGAYLSCGDLWLCLSLDPQRRVTPPEESDYTHYAFSIS
EADFASFAARLEVAGVAVWKLNRSEGASHYFLDPDGHKLELHVGSLAQRLAAC
>GCA_900144895
MLTGLNHLTLAVAELPASIAFYRDLLGFRLEARWDQGAYLELGSLWLCLSREPQYGGPAADYTYYAFGIAAAD
FARFAAHLRAHGVHEWKQNRSEGDSFYFLDPDGHRLEAHVGDLRSRLAACRQA
>GCA_900144845
MLTGLNHLTLAVAELPASIAFYRDLLGFRLEARWDQGAYLELGSLWLCLSREPQYGGPAADYTYYAFGIAAAD
FARFAAHLRAHGVHEWKQNRSEGDSFYFLDPDGHRLEAHVGDLRSRLAACRQ
>GCA_000498215
VSNLQSSLTFWRDLLGLQLHAEWGTGAYLTCGDLWLCLSYDVSRSYVAPQKSDYTHYAFSIAPEDFEPFSYKL
KQSGVTVWKDNKSEGQSFYFLDPDGHKLELHVGDLASRLAQCRERPYSGMRF
>GCA_001399815
MLSGLNHLTLAVSQLAPSVAFYQQLLGMTLHARWDSGAYLSCGDLWLCLSLDPQRRVTPPEESDYTHYAFSIS
EADFASFAARLEAAGVAVWKLNRSEGASHYFLDPDGHKLELHVGSLAQRLA
>GCA_001067795
LCMCLSRSRRVQPLARWSSITCQNSSLRADSIAFFAGTRSRAIRLTYAFGIAAADFARFAAQLRAHGVREWKQ
NRSEGDSFYFLDPDGHRLEAHVGDLRSRLAACRQAPYAGMRF
>GCA_000465635
MKLSGLNHLTISVANVDRSFNFYKDVLGFTPKAKWKKGAYLSLGELWLCLSLDEVSISSDYTHYCLSISEDNI
DEFRQKIKMMNIPEWKTNQSEGESIYFLDPDGHKLEVHVGNLSSRLESCKK
>GCA_000572265
DLPASIAFYRDLLGFRLEARWDQGAYLELGSLWLCLSREPQYGGPAADYTHYAFGIAAADFARFAAQLRAHGV
REWKQNRSEGDSFYFLDPDGHRLEAHVGDLRSRLAACRQAPYAGMRFAD
>GCA_000414255
MLTGLNHLTLAVADLPASIAFYRDLLGFRLEARWDQGAYLELGSLWLCLSREPQYGGPAADYTHYAFGIAAAD
FARFAAQLRAHGVREWKQNRSEGDSFYFLDPDGHRLEAHVGDLRSRLAA
>GCA_001641855
SLTFWRDLLGLQLHAEWGTGAYLTCGDLWLCLSYDVSRSYVAPQKSDYTHYAFSIAPEDFEPFSYKLKQSGVT
VWKDNKSEGQSFYFLDPDGHKLELHVGDLASRLAQCRERPYSGMRF
>GCA_000350325
MLSGLNHLTLAVSQLAPSVAFYQQLLGMTLHARWDSGAYLSCGDLWLCLSLDPQRRVTPPEESDYTHYAFSIS
EADFASFAARLEAAGVAIWKLNRSEGASHYFLDPDGHKLELHV
>GCA_001268725
FAGVAAHAEDTGAYLTCADLWICLSYDVSRNYVAPQESDYTHYAFSIAAEDFEPFAYKLKQAGVTVWKDNKSE
GQSFYFLDPDGHKLELHVDLASRLEQYREKPYSGMRF
>GCA_001975285
MLQSLNHLTLAVSNLQTSLTFWRDLLGLQLHAEWDTGAYLTCGDLWVCLSYDVSRNYVAPQESDYTHYAFSIA
AEDFEPFSYKLKQSGVTVWKDNKSEGQSFYFVDPDGHKL
>GCA_000615565
MLTGLNHLTLAVADLPASIAFYRDLLGFRLEARWDQGAYLELGSLWLCLSREPQYGGPAADYTHYAFGIAAAD
FARFAAQLRAHGVREWKQNRSEGDSFYFLDPDGHRLE
>GCA_001247585
LTAEWDTGAYLTCGDLWVCLSYDVSCNYVAPQECDYTHYAFSIAPEDFEPFSYKLKQAGVTVWKDNKSEGQSF
YFLDPDGHKLELHVGDLASRLAQCREKPYSGMRF
>GCA_001750425

LEARWDQGAYLELGSLWLCLSREPQYGGPAADYTHYAFGIAAADFARFAAQLRAHGVREWKQNRSEGDSFYFL
DPDGHRLEAHVGDLRSRLAACRQAPYAGMRFAD
>GCA_001605875
EARWDQGAYLELGSLWLCLSREPQYGGPAADYTHYAFGIAAADFARFAAQLRAHGVREWKQNRSEGDSFYFLD
PDGHRLEAHVGDLRSRLAACRQAPYAGMRFAD
>GCA_000603845
AEWDTGAYLTCGDLWVCLSYDVSCNYVAPQECDYTHYAFSIAPEDFEPFSYKLKQAGVTVWKDNKSEGQSFYF
LDPDGHKLELHVGDLASRLAQCREKPYSGMRF
>GCA_001037435
WKHAGTRAPISNWVHCGCACPGSRSTAGRPRTTYAFGIAAADFARFAAQLRAHGVREWKQNRSEGDSFYFLDP
DGHRLEAHVGDLRSRLAACRQAPYAGMRFAD
>GCA_001045515
MLTGLNHLTLAVADLPASIAFYRDLLGFRLEARWDQGAYLELGSLWLCLSREPQYGGPAADYSVHDQPLPLRH
AGTRSRRETRPASAVPYL
>GCA_001061585
GYTNFPDLICHSHPYQTENNTVQYAFGIAAADFARFAAQLRAHGVREWKQNRSEGDSFYFLDPDGHRLEAHVG
DLRSRLAACRQAPYAGMRF
>GCA_000300735
LSYDVSRNYVATQEGDYTHYAFSIAPEDFESFSYKLKQAGVTVWKDNKSEGSFYFLDPDGHMLELHVGDLASR
LVQCRERLYSGMRF
>GCA_000188335
LSYDVSRNYVAPQECDYTHYAFSIAPEDFEPFSYKLKQAGVTVWKDNKSEGQSLYFLDPDGHKLELHVGDLAS
RLAQCREKPYSGMRF
>GCA_001777135
MLSGLNHLTLAVSQLAPSVAFYQQLLGMTLHARWDSGAYLSCGDLWLCLSLDPQRRVTPPEESDYTHYAFSIS
EADFASFAPALRL
>GCA_001766715
MLSGLNHLTLAVSQLAPSVAFYQQLLGMTLHARWDSGAYLSCGDLWLCLSLDPQRRVTPPEESDYTHYAFSIS
EADFASFAPALR
>GCA_900085705
MLSGLNHLTLAVSQLAPSVAFYQQLLGMTLHARWDSGAYLSCGDLWLCLSLDPQRRVTPPEESDYTHYAFSIS
EADFASFAARLE
>GCA_001901735
MLSGLNHLTLAVSQLAPSVAFYQQQLGMTLHARWDSGAYLSCGDLWLCLSLDPQRRVTPPEESDYTHYAFSIS
EADFASFAPALR
>GCA_001061895
MLTGLNHLTLAVADLPARIAFYRDLLGFRLEARWDQGAYLELGSLWLCLSREPQYGGPAADYTHRRADNAIGV
I
>GCA_001893225
PQESDYTHYAFSVAEEEFAGVVALLAQAGAEVWKDNRSEGASYYFLDPDGHKLELHVGNLAQRLAACRERPYK
GMVFFD
>GCA_000617365
AYQKSDYTHYAFSIAPEDFEPFSYKLKQSGVTVWKDNKSEGQSFYFLDPDGHKLELHVGDLASRLAQCRERPY
SGMRF
>GCA_001863405
MKLSGLNHLTISVANVDRSFNFYKDILGFTPKAKWKKGAYLSLGELWLCLSLDEVSVSSDYTHYCLSISEDDI
DEFRQ
>GCA_000962405
MLQSLNHLTLAVSNLQTSLTFWRDLLGLQLHAEWDTGAYLTCGDLWVCLSYDVSCNYVAPQECDYTHYAFSIA
PEDF
>GCA_000705235
MLTGLNHLTLAVADLPASIAFYRDLLGFRLEARWDQGAYLELGSLWLCLSREPQYGGPAADYTHYAFGIAAAD
F
>GCA_000283055

YTHYAFGIAAADFARFAAQLRAHGVREWKQNRSEGDSFYFLDPDGHRLEAHVGDLRSRLAACRQAPYAGMRFA
D
>GCA_001075925
SCGDLWLCLSLDPQRRVTPPEESDYTHYAFSISEADFASFAARLEAAGVAIWKLNRSEGASHYFLDPDGHKLE
L
>GCA_000473745
MLTGLNHLTLAVADLPASIAFYRDLLGFRLEARWDQGAYLELGSLWLCLSREPQYGGPAADYTHYAFGIAA
>GCA_000173695
YCPYRHLSYCVSRNYVAPQESDYTHYAFSIAAEDFEPFSYKLKQSGVTVWKDNKSEGQSFYFVDPDGHKL
>GCA_000747825
YCPYRHLLYYVSRNYVAPQESDYTHYAFSIAAEDFEPFSYKLKQSGVTVWKDNKSEGQSFYFVDPDGHKL
>GCA_001058735
VGNKDWNVLTTIMDSDGQTSFYFLDPDGHKLELHVGSLAARLAACREKPYAGMVFTSDEA
